# Evidence for overlapping genetic architecture between lifestyle factors and severe mental disorders with different patterns across diagnoses

**DOI:** 10.1016/j.ebiom.2026.106304

**Published:** 2026-05-27

**Authors:** Linn Rødevand, Zillur Rahman, Piotr Jaholkowski, Nadine Parker, Unnur Anna Valdimarsdóttir, Olav Bjerkehagen Smeland, Markos Tesfaye, Pravesh Parekh, Oleksandr Frei, Srdjan Djurovic, Nils Eiel Steen, Anders Martin Dale, Alexey Shadrin, Ole Andreas Andreassen

**Affiliations:** a Centre for Precision Psychiatry, Division of Mental Health and Addiction, Oslo University Hospital, and Institute of Clinical Medicine, University of Oslo, Oslo, Norway; b Institute of Environmental Medicine, Unit of Integrative Epidemiology, Karolinska Institute, Stockholm, Sweden; c Center of Public Health Sciences, Faculty of Medicine, University of Iceland, Reykjavík, Iceland; d Institute for Genomics in Health & Department of Psychiatry and Behavioral Sciences, SUNY Downstate Health Sciences University, Brooklyn, NY, USA; e Center for Multimodal Imaging and Genetics, J. Craig Venter Institute, La Jolla, CA, USA; f Department of Pharmacy, Section for Pharmacology and Pharmaceutical Biosciences, University of Oslo, Oslo, Norway; g Department of Medical Genetics, Oslo University Hospital and University of Oslo, Oslo, Norway; h Section for Clinical Psychosis Research, Division of Mental Health and Addiction, Oslo University Hospital, Oslo, Norway; i Division of Mental Health and Substance abuse, Diakonhjemmet Hospital, Oslo, Norway; j Oslo University Hospital, Oslo, Norway; k University of California San Diego, La Jolla, CA, USA

**Keywords:** Schizophrenia, Bipolar disorder, Major depression, Lifestyle, Body mass index, GWAS

## Abstract

**Background:**

Severe mental disorders (SMDs) are associated with unhealthy lifestyle, contributing to increased risk of comorbid cardiovascular disease. Genetic factors influence both SMDs and lifestyle behaviours, but their genetic relationships remain unclear. Here, we aimed to unravel the shared genetic architecture of SMDs and lifestyle factors. Additionally, we assessed if genetic propensity to SMDs predicts body mass index (BMI) and lipids through lifestyle factors.

**Methods:**

We analysed genome-wide data on major depression (MD) (N = 480,359), schizophrenia (SCZ) (N = 130,644), bipolar disorder (BIP) (N = 353,889) and self-reported lifestyle factors (N = 266,048–606,820), including food intake, physical activity, sedentary behaviours, and accelerometer-assessed activity from All of Us (N = 30,132) and UK Biobank (N = 91,105) to obtain objective measures for sensitivity analysis. We estimated the shared genetic architecture using bivariate MiXeR. Shared genetic loci were identified using conjunctional false discovery rate and mapped to genes, which were subject to enrichment analyses. We applied structural equation modelling (SEM) to assess if lifestyle mediates the relationship between polygenic risk score for SMDs and BMI and lipids. People with lived experience were involved in the research.

**Findings:**

There was extensive genetic overlap between lifestyle factors and SMDs, with different patterns of effects. MD was genetically correlated with less physical activity and more sedentary behaviour. SCZ and BIP displayed opposite patterns with genetic associations with less sedentary behaviour, more physical activity and healthier food intake. This divergent pattern across SMDs was largely consistent using accelerometer-assessed activity. We identified 551 shared loci, implicating biological processes related to neurodevelopment and synaptic and neuronal properties. Further analyses indicated that lifestyle factors partly mediate the relationship between genetic risk for SMDs and BMI and lipids.

**Interpretation:**

The results show a genetic propensity towards unhealthier lifestyle behaviours in MD, while SCZ and BIP displayed a divergent pattern. The genetic correlations reflecting mixed effect directions may also imply subgroups with different genetic propensity, which can form the basis for risk stratification and more tailored lifestyle interventions and personalised treatment.

**Funding:**

Research Council of Norway (grants, 273291, 273446, 300309, 324252, and 326813), South-East Norway Regional Health Authority (grants 2023-031 and 2022-073), NordForsk (University Cooperation Grant 164218, PreciMENT), European Union's Horizon 2020 Research and Innovation Programme (grant 847776, CoMorMent; grant 964874, RealMent), and the National Institutes of Health (grant R01MH125938).

**Research in context**

**Evidence before this study**

Unhealthy lifestyle factors are prevalent among people with SMDs, contributing to increased risk of cardiovascular disease. Twin and family studies indicate a substantial heritability of severe mental disorders (SMDs) and lifestyle behaviours, including food intake, physical activity and sedentary behaviour. However, it remains unclear how genetic factors may influence the relationship between SMDs and lifestyle, and how this is linked to cardiovascular disease (CVD) risk factors, such as body mass index (BMI) and lipids. We performed a systematic search of literature in PubMed and Google Scholar to assess the genetic relationship between SMDs (i.e., schizophrenia (SCZ), bipolar disorder (BIP), major depression (MD)) and lifestyle factors. The search involved terms related to genetics (“genome wide association study”, “genetic”, “polygenic risk score”, and “mendelian randomisation”), SMDs (“severe mental disorder”, “severe mental illness”, “major psychiatric disorder”, “schizophrenia”, “bipolar disorder”, “major depression” and “major depressive disorder”), lifestyle (“lifestyle factors”, “lifestyle behaviours”, “diet”, “food intake”, “physical activity”, “exercise”, “sedentary behaviour”, “accelerometer”, and “Fitbit”), BMI (“body mass index”, “weight gain”, and “obesity”), and lipids. Prior studies indicate that MD is genetically correlated with higher BMI and triglycerides (TG) levels, alongside lower high-density lipoprotein (HDL), while SCZ and BIP exhibit a negative genetic association with BMI and a minimal genetic correlation with these lipids. However, research on the genetic relationship between SMDs and lifestyle factors is scarce and has provided inconsistent findings. Additionally, there is a lack of studies assessing whether genetic risk for SMDs is associated with BMI and lipids via their relationship to lifestyle behaviours.

**Added value of this study**

Using a comprehensive approach to large GWAS datasets, we aimed to improve the understanding of the genetic relationship between SMDs and lifestyle factors. We analysed self-reported lifestyle factors (food intake, physical activity and sedentary behaviour) and accelerometer-assessed activity. We found that MD is associated with a genetic propensity to less physical activity and more sedentary behaviour, which seems to play a role in the increased BMI and lipid disturbances associated with this disorder. SCZ and BIP displayed a different pattern, including a genetic association with healthier food intake, more physical activity and less sedentary behaviour, which mediated the association with lower BMI and TG and higher HDL levels. These divergent patterns across SMDs were observed both at the genome-wide genetic correlation level and among the specific shared loci, and were largely consistent with results from accelerometer-assessed activity. We discovered over 500 shared loci between SMDs and lifestyle factors, implicating neurobiological mechanisms. Further, the findings of mixed effect directions among shared loci suggest subgroups of patients with increased genetic propensity to unhealthy lifestyle factors.

**Implications of all the available evidence**

The findings suggest a genetic component to reduced physical activity in MD, in line with an increased genetic risk of obesity and lipid disturbances. In SCZ and BIP, unhealthy food intake, physical inactivity, and metabolic disturbances are more likely to be driven by factors other than illness-related loci, including medication side-effects, amotivation and socioeconomic challenges. Still, considerable genetic heterogeneity is expected within the diagnostic groups given their high polygenicity and shared genetic architecture with lifestyle factors involving mixed effect directions. Taken together, while SCZ and BIP on average show a genetic propensity toward healthier lifestyle factors, evidence indicates subgroups of patients within these diagnoses with an increased risk for both SMDs and unhealthy lifestyles. The findings have implications for risk prediction and the development of more targeted lifestyle interventions. Additionally, further identification of at-risk groups may inform design of public health programs for cardiometabolic health, while taking mental health, especially MD, into account.

## Introduction

People with severe mental disorders (SMDs), including schizophrenia (SCZ), bipolar disorder (BIP) and major depression (MD), have 10–20 years shorter life expectancy than the general population, attributed in a large part to higher rates of cardiovascular disease (CVD).^1^^,^^2^ Lifestyle behaviours including smoking, unhealthy diet, and low physical activity levels are common in SMDs, contributing to the CVD comorbidity.^3^^,^^4^ Lifestyle interventions have short-term effects, while long-term changes are difficult to achieve,^5^ and have limited efficacy in decreasing CVD risk in SMDs.^1^^,^^2^ A better understanding of the underlying causes of the relationship between SMDs and lifestyle behaviours is needed to develop more effective interventions, to reduce the CVD comorbidity and increase life expectancy.

The unhealthy lifestyle behaviours in SMDs are related to several factors, including symptoms, adverse effects of psychotropic medications, and socioeconomic challenges.^4^^,^^6^ Genetic factors may also play a significant role. The heritability is moderate-high for SMDs (40–80%)^7^ and lifestyle behaviours, including physical activity (30–70%),^8^^,^^9^ dietary habits (20%–50%)^10^^,^^11^ and smoking (50%).^12^ SMDs are associated with an increased genetic propensity to smoking,^13^, ^14^, ^15^ while their genetic relationship with food intake and physical activity is poorly understood. Some data indicate that MD is negatively genetically correlated with physical activity,^15^^,^^16^ in line with a positive genetic correlation with body mass index (BMI).^17^ In SCZ and BIP, studies on the genetic relationship with diet and physical activity have provided inconsistent results.^16^^,^^18^, ^19^, ^20^, ^21^ Further, SCZ and BIP have been linked to a genetic liability to lower BMI,^13^^,^^17^^,^^22^^,^^23^ although findings are inconclusive for BIP. Moreover, while MD is genetically associated with dyslipidemia,^15^ SCZ and BIP demonstrate no or minimal genetic correlation with lipids (e.g., triglycerides (TG) and high-density lipoprotein cholesterol (HDL)).^13^^,^^22^ These genetic results for SCZ and BIP differ from the clinical findings of higher obesity and dyslipidemia rates in the patient populations,^1^ suggesting the involvement of psychotropic medications and modifiable lifestyle factors, such as diet and physical activity. However, whether there are genetic variants jointly influencing lifestyle behaviours and SMDs is yet to be clarified.

Previous studies have mostly applied measures of genetic correlations or polygenic risk score (PRS), which do not offer a complete depiction of the genetic relationship between complex phenotypes.^24^ In scenarios of a mixture of concordant and discordant effect directions, there may be polygenic overlap despite an absence of genetic correlation or PRS associations.^24^, ^25^, ^26^ Thus, there is a need for complementary tools that can identify shared genetic variants irrespective of the genetic correlation, to uncover joint molecular mechanisms. Furthermore, mediation tools^27^ can offer insights into the involvement of lifestyle in the relationship between genetic liability to SMDs and CVD risk factors. Here, we applied a comprehensive approach to investigate key lifestyle behaviours (food intake^28^ and physical activity^16^) and their genetic relationship with SCZ,^29^ BIP,^14^ and MD,^30^ leveraging increase in the power of genome-wide association studies (GWASs). We also conducted a GWAS to obtain data on accelerometer-assessed activity for more objective measures in sensitivity analyses. We estimated the total number of unique and shared genetic variants with MiXeR,^31^ and identified specific shared loci with conjunctional false discovery rate (conjFDR),^25^ followed by functional analyses (Open Targets, FUMA).^32^^,^^33^ To assess if lifestyle behaviours mediate the association between SMDs and BMI and lipids, we employed a structural equation modelling (SEM) approach.^27^ In addition to these primary analyses, bidirectional mendelian randomisation (MR)^34^ was performed to test potential causal relationships between SMDs and lifestyle factors.

## Methods

### Participant samples

#### GWAS summary statistics

We obtained GWAS summary statistics from samples of European ancestry to avoid bias due to differences in linkage disequilibrium (LD) structure across ancestries (Table 1). Summary statistics on SCZ (N = 130,644),^29^ BIP (N = 353,889),^14^ and MD (N = 480,359)^30^ were acquired from the Psychiatric Genomics Consortium. The SCZ dataset consisted of 53,386 individuals with schizophrenia or schizoaffective disorder and 77,258 controls of European descent.^29^ The BIP dataset comprised 41,917 patients and 371,549 controls.^14^ The MD GWAS included 135,458 patients and 344,901 controls.^30^ The MD data were also retrieved from the PGC and 23andMe. The term MD was used instead of the diagnostic term “major depressive disorder” because around half of the cases were identified by self-report (i.e., diagnosis or treatment for clinical depression by a medical professional). For details about the ascertainment and diagnostic criteria, see the original publications.^14^^,^^29^^,^^30^

**Table 1:** Overview of the GWAS samples.

Phenotype	Sample size (N)	Source
SCZ	53,386 patients; 77,258 controls	Trubetskoy et al.^29^
BIP^a^	40,463 patients; 313,436 controls	Mullins et al.^14^
MD^a^	121,198 patients; 329,421 controls	Wray et al.^30^
Food intake		Pirastu et al.^28^
Healthy Food^b^	386,047	
Meat^c^	266,048	
Physical activity and sedentary behaviour		Wang et al.^16^
PhysAct^d^	606,820	
Screen^e^	526,725	
SedWork^f^	372,605	
SedCom^g^	159,606	

SCZ, schizophrenia; BIP, bipolar disorder; MD, major depression; Healthy Food, healthy food intake; Meat, meat consumption; PhysAct, Moderate-to-vigorous intensity physical activity; Screen, leisure screen time; SedWork, sedentary behaviour at work; SedCom, sedentary commuting.

a The UK Biobank was excluded from the bipolar disorder sample (N = 1454 cases; 58,113 controls) and major depression sample (N = 14,260 cases; 15,480 controls) before conditional/conjunctional false discovery rate, polygenic risk score and mendelian randomisation analyses.

b Healthy Food included vegetables, fruits and fish.

c Meat included beef, pork, processed meat, lamb and poultry.

d PhysAct involves various forms of moderate/vigorous activity, such as jogging, swimming and cycling.

e Screen includes time spent sedentary using screen across devices, such as watching television, playing video games, and sitting at the computer.

f SedWork involves sedentary behaviour at work, such as mostly sitting and no heavy lifting.

g SedCom includes sedentary commuting, such as usually driving a car or bus instead of walking or bicycling to travel.

In addition, we used GWAS data on lifestyle behaviours from the UKB and other European cohorts (Table 1). Food intake (N = 266,048–386,047)^28^ was evaluated through a self-reported questionnaire about consumption frequency over the past year, which was standardised to weekly consumption.^28^ We focused on two food intake categories extracted from principal component (PC) analyses in the original GWAS dataset: 1) higher meat consumption (including beef, pork, processed meat, lamb, and poultry; hereafter *Meat*), and 2) intake of healthier foods (including fish, fruits, and vegetables; hereafter *Healthy Food*). We focused on these dietary components due to their roles in cardiometabolic health^35^ and their genetic relationships with obesity.^28^ While specific nutrients are important, the overall tendency to consume certain foods are more influential on cardiometabolic disturbances.^28^ Data on self-reported physical activity included moderate-to-vigorous intensity physical activity during leisure time (*PhysAct*), and sedentary behaviours, encompassing leisure screen time (*Screen*), sedentary behaviour at work (*SedWork*), and sedentary commuting (*SedCom*).^16^ Sample sizes ranged from 159,606 for SedCom to 606,820 for PhysAct (Table 1). Screen was normally distributed and could be analysed as a continuous outcome in the original GWAS, while the other three phenotypes were analysed as dichotomous outcomes (see details in the original GWAS).^16^ These lifestyle factors were chosen given their relevance to CVD risk.^36^ Moreover, the lifestyle behaviours assessed were influenced by the necessity for well-powered datasets required by the statistical tools.^25^^,^^31^ Summary statistics for tobacco smoking (N = 1,232,091)^37^ were retrieved for Supplementary Analyses: Current smoking, which is high prevalent in SCZ and BIP,^1^ is linked to lower BMI in epidemiological studies,^38^ and SCZ and BIP are genetically associated with lower BMI^13^^,^^23^ and increased propensity to smoking.^13^^,^^14^ Thus, we aimed to elucidate the potential role of smoking in the genetic relationship between SCZ, BIP and BMI.

GWAS data of accelerometer-derived activity, including the phenotypes overall physical activity and sedentary behaviour, were obtained from the UKB (n = 91,105) (see the original publication for detailed sample characteristics).^39^ This dataset was meta-analysed with GWAS results that we generated from accelerometer-assessed physical activity in the All of Us Research Program.^40^ The program aims to collect health-related data from over 1 million adults across the Unites States to facilitate precision medicine. As of February 2025 (v8 data release), 59,020 participants in All of Us have provided Fitbit data, including data from participants' personally owned Fitbits and study-provided Fitbit devices to improve representation among those without a personally owned Fitbit.^40^^,^^41^ The device uses a 3-axis accelerometer to compute daily minutes in each of these activity zones: sedentary, lightly active, fairly active and very active. The activity zones are based on Metabolic equivalent of task (MET) definitions,^42^ and sedentary behaviour has a MET score of ≤ 1.5 and occurs in a sitting, lying, or reclining posture.^42^ The data was used to derive an average of daily physical activity (AccPhysAct) and sedentary behaviour (AccSed). The analysed sample consisted of 30,132 individuals identified as White based on self-report (mean age (SD) = 59.37 (16.24) years; 66.99% female) after applying similar inclusion criteria as described by Doherty et al.^39^^,^^43^ in the UKB GWAS (at least 3 days of data in each 1-h period of the 24-h cycle during a 7-day period). Due to the nature of the data (summarised daily measures per person and not raw accelerometers signals), we could not perform the calibration that was used in the UKB GWAS.

We excluded overlapping samples before the analyses except from MiXeR, which corrects for sample overlap. Accordingly, we used summary statistics excluding UKB from the BIP sample (N = 40,463 patients; N = 313,436 controls) and MD sample (N = 121,198 patients; N = 329,421 controls).

To assess the generalisability of the findings across different ancestral groups, we also conducted analyses in non-European datasets. GWAS summary statistics were retrieved from individuals of East Asian (EAS) genetic ancestry for SCZ^44^ (N = 29,519 cases; N = 44,392 controls), BIP^45^ (N = 4479 cases; N = 75,725 controls), MD^46^ (N = 13,893 cases; N = 155,912 controls), and dietary factors^47^ (N = 165,084), including consumption of meat, vegetables, and fish, while data on fruit intake was unavailable. For further information, see the original publications.^44^, ^45^, ^46^, ^47^

### Individual-level data in UKB

We used individual-level data on BMI, lipids (TG and HDL), lifestyle factors (Healthy Food, Meat, PhysAct, and Screen), and current smoking from UKB participants (accession number 27412). Healthy Food was the first PC of cooked vegetables, raw vegetables or salad, oily fish, non-oily fish, fresh fruits, and dried fruits, in line with Pirastu et al.^28^ Meat was the first PC of beef, processed meat (including pork and other processed meat), poultry, lamb and mutton.^28^ PhysAct involved moderate-to-vigorous physical activity during leisure time weekly, and Screen involved time spent using screen during leisure time daily, consistent with Wang et al.^16^ A total of 235,147 participants (N = 126,789 female; N = 108,358 male) were included with both BMI, lipids (TG and HDL) and lifestyle data. The participants had a mean (standard deviation, SD) age of 56.94 (7.98), and a BMI of 27.22 (4.58). The mean (SD) levels of TG and HDL were 1.73 (1.01) and 1.46 (0.38), respectively. 8.9% of the participants were current smokers, while the rest were either former smokers or had never smoked. We used a subsample of UKB as a training sample to find the threshold of the best-performing PRS. This subsample included 60,052 participants (N = 33,717 female; N = 26,335 male), with a mean (SD) age of 57.10 (8.03), and mean BMI (SD) of 27.51 (4.77).

People with lived experience were involved in the rationale, study design and discussion of the results.

### Ethics

The National Health Service National Research Ethics Service (ref. 11/112 NW/0382) approved the UKB project, and data was obtained under accession number 27412. Individual-level data from All of Us was accessed through the All of Us Research Program Researcher Workbench and the research program is approved by the National Institutes of Health. The Regional Committee for Medical Research Ethics (REC) South-East Norway (#2009/2485) approved the use of individual-level data from UKB and All of Us. The individual GWASs were approved by their respective ethical committees. Written informed consent was obtained from all participants, and the work has been conducted in accordance with the Declaration of Helsinki. The Norwegian REC evaluated the analytical approach including summary statistics and determined that no additional review board approval was needed as no individual data were used.

### Statistical analyses

#### MiXeR

We applied MiXeR^31^ to quantify the polygenic overlap between SMDs and lifestyle factors. MiXeR models the distribution of additive SNP effects as a point–normal mixture, in which each variant belongs either to a null component (zero effect) or to a non-zero component with normally distributed effects. This framework models the full distribution of GWAS association statistics rather than genome-wide significant loci. In cross-trait analyses, MiXeR extends this framework to jointly model SNP effects across traits, allowing shared variants to have either concordant or discordant effect directions. Inference is performed using GWAS z-scores while modelling the effects of LD and allele frequency. This allows MiXeR to distinguish between multiple correlated association signals arising from LD and the underlying number of independent variants influencing the traits.^31^ Within this framework, variants assigned to the non-zero component are interpreted as trait-influencing variants, representing independent genetic effects after accounting for LD. Further, MiXeR assumes that the distribution of additive SNP effects is independent of allele frequency, LD, and genomic location.^31^ While these assumptions may be imperfectly satisfied for some traits, simulations in the original study demonstrated that MiXeR estimates are robust to a range of such violations.^31^ As recommended, we evaluated model fit for each analysis as outlined below.

First, we used univariate MiXeR to estimate the SNP-based heritability and polygenicity, which is expressed as the number of trait-influencing variants with the strongest effects which jointly account for 90% of SNP heritability of the phenotype. The threshold is set to 90% to avoid extrapolating model parameters into variants with infinitesimally small effects.^31^ Second, we used bivariate MiXeR to estimate the total number of shared variants and the fraction of SNPs with concordant effects in the shared genetic component. Additionally, MiXeR estimates the Dice coefficient (i.e., the proportion of SNPs shared by two phenotypes out of the total number of SNPs influencing both phenotypes) and the genetic correlation between two phenotypes (Supplementary Methods).^31^

To assess model fit, we calculated the difference between the Akaike Information Criterion (AIC) of the best-fitting MiXeR estimates and a reference model. For univariate MiXeR, the reference was a baseline infinitesimal model (Supplementary Methods).^31^ In the bivariate MiXeR analysis, the estimated polygenic overlap was evaluated against two constrained alternative models: a minimum-overlap model, representing the smallest overlap compatible with the observed genetic correlation, and a maximum-overlap model, in which all variants influencing the less polygenic trait also influence the more polygenic trait. Positive AIC differences indicate that the MiXeR-estimated overlap provides a better fit to the GWAS summary statistics than the constrained alternative.

For Supplementary Analyses, we used trivariate MiXeR, which extends bivariate MiXeR to assess genetic overlap among three phenotypes (i.e., each SMD and two lifestyle factors).^48^ Trivariate MiXeR assesses if the pattern of genetic overlap among three phenotypes differs from the overlap patterns expected based on the three bivariate overlaps under the naïve assumption of maximum entropy (Supplementary Method).^48^ We also used trivariate MiXeR to assess shared genetics between each of SCZ and BIP, BMI and smoking.

Major histocompatibility complex (MHC) region was excluded prior to MiXeR since the complicated LD structure of MHC may bias the estimates, following the recommendation for this method.^31^

### Conjunctional false discovery rate (ConjFDR)

We generated conditional quantile–quantile (Q–Q) plots to visualise cross-trait enrichment, which is indicated by an increase in SNPs associated with a primary phenotype (e.g., SCZ) as a function of the strength of the association with a secondary phenotype (e.g., PhysAct).^25^ The conditional (cond) FDR approach was used to increase discovery of specific genetic loci associated with SMDs and lifestyle factors at condFDR value < 0.01.^13^^,^^25^ This method uses cross-trait enrichment observed in the conditional Q–Q plots to re-rank the test statistics of a primary phenotype based on a secondary phenotype.^25^ To identify shared genetic loci, we applied conjFDR with a threshold of conjFDR <0.05.^13^^,^^25^ The conjFDR statistic is defined as the maximum of two condFDR values (the primary phenotype conditioned on the secondary phenotype, and vice versa). We excluded SNPs in the extended MHC and 8p23.1 regions due to extensive LD within these regions before fitting the conjFDR model to avoid inflation, which is part of the standard protocol.^25^ This procedure involves removing SNPs in these regions before constructing the conditional Q–Q plots and FDR model, while allowing for the detection of SNPs in these regions after the FDR model has been fitted, because the shared genetic signal may be highly biologically relevant (Supplementary Methods).^17^^,^^22^^,^^25^

### Genomic loci definition

We used the Functional Mapping and Annotation (FUMA)^32^ protocol to delineate the genomic regions of the identified SNPs. Independent significant SNPs were defined as SNPs with condFDR <0.01 or conjFDR <0.05 and r^2^< 0.6. The independent SNPs in approximate LD with each other (r^2^< 0.1) were selected as lead SNPs. Distinct genomic loci were delineated by identifying candidate SNPs in LD (r^2^≥ 0.6) with significant independent SNPs. Loci less than 250 kb apart were merged (Supplementary Methods).

### Effect directions and genetic correlations

We evaluated the directional effects of the shared loci between SMDs and lifestyle factors by comparing their *Z*-scores from the respective GWAS summary statistics. LD score regression (LDSC)^49^ was used to estimate the genome-wide genetic correlations (*r*_*g*_), providing a summary measure of the correlation of effect sizes between the two phenotypes (Supplementary Methods).

### Validation in East Asian ancestry samples

To assess whether the overall patterns for SMDs and dietary factors observed in the primary discovery cohorts were replicable within individuals of East Asian (EAS) genetic ancestry (Supplementary Methods),^44^, ^45^, ^46^, ^47^ we used LDSC^49^ and bivariate MiXeR.^31^ Sufficiently powered non-European physical activity datasets were not publicly available.

### Functional annotation

Lead SNPs were mapped to putative causal genes using the online source Open Targets Genetics,^33^ a machine learning approach that integrates information about gene distance and functional features to map SNPs to genes (Supplementary Methods). Next, mapped genes from each pairwise analysis were used for gene-set analyses focussing on Gene Ontology (GO) terms using FUMA (Supplementary Methods).^32^ In further enrichment analyses to improve power, we combined the genes mapped to lead SNPs associated with physical activity and sedentary behaviours (PhysAct, Screen and SedWork), as well as the genes linked to food intake (Healthy Food and Meat), for each SMD separately. Before the enrichment analyses, we excluded genes in the MHC and 8p23.1 regions to prevent potential inflation due to the strong LD.^24^^,^^32^ As sensitivity analyses, we also performed enrichment analyses including genes in these genomic regions to test the robustness of the results.

### Structural equation modelling (SEM)

We investigated the association between PRS for SMDs, BMI and lipids (TG and HDL), and the mediating role of lifestyle factors, in a population sample from the UKB. First, we applied PRSice (version 2.3.5)^50^ to calculate PRS for SMDs based on summary statistics from the GWASs of SMDs (Supplementary Methods). Next, we assessed the association between PRS for SMDs (predictor), BMI (outcome), and lipids (outcome), with each lifestyle behaviour (Healthy Food, Meat, PhysAct and Screen) as a potential mediator in separate SEM models with R package lavaan.^27^ Furthermore, we assessed if current smoking mediates part of the association between SCZ and BIP PRSs and BMI. SEM was performed after evaluating the distribution of the data as SEM assumes multivariate normality of residuals. We generated figures of the univariate distribution of residuals and the relationships between variables' residuals after transformation with rank-based inverse normal transformation (RINT).^51^ In each model, we adjusted for sex, age, genetic array, and the first ten genetic PCs, following prior convention.^52^^,^^53^ Sex was self-reported by the participants and recorded as a binary variable (male/female). We reported standardised coefficients (β).^27^ See further information in Supplementary Methods.

### Mendelian randomisation (MR)

As Supplementary Analyses, we conducted bidirectional MR to assess potential causal relationships between SMDs and lifestyle factors, with TwoSampleMR (version 0.5.7, R version 4.3.1).^34^ Summary statistics from GWASs of SMDs^14^^,^^29^^,^^30^ and self-reported lifestyle factors^16^^,^^28^ described above were used. MR relies on three core assumptions: (1) the genetic variants being used as instruments are associated with the exposure; (2) the instruments are not associated with confounders of the exposure–outcome association, and (3) there is no independent pathway between the instruments and outcome other than through the exposure.^34^ The use of multiple MR methods with different assumptions and approaches to account for violated assumptions is necessary to improve confidence in causal interpretations.^34^ Therefore, we calculated MR regression coefficients using the inverse variance weighted method,^54^ MR-Egger,^55^ and weighted median.^56^ We also used Pleiotropy Residual Sum and Outlier (MR-PRESSO version 1.0, R version 4.3.1).^57^ Logistic regression was used for binary disease outcomes, and linear regression was used for continuous outcomes. We focused on associations if at least two MR methods provided significant results after correction for multiple testing with FDR adjustment, indicating support for a potential causal relationship (Supplementary Methods).^58^

### Genome-wide analysis of accelerometer-assessed activity

We conducted a GWAS of accelerometer data derived from a Fitbit tracker from the All of Us.^41^ We derived two variables: an average of physical activity (AccPhysAct), aggregated from daily minutes across various intensities, and daily sedentary time (AccSed) (Supplementary Methods).^41^ Henceforth, we use “accelerometer-assessed activity” to collectively refer to both AccPhysAct and AccSed. After applying inclusion criteria to obtain sufficiently reliable activity data,^39^ the sample consisted of 30,132 participants identified as White. For this sample, we obtained quality controlled population level Allele Count/Allele Frequency (ACAF) threshold callset of whole genome sequencing (WGS) data (Supplementary Methods).^59^ The GWAS in All of Us was performed using PLINK2^60^ in whole genome sequencing variant data including variants with minor allele frequency >0.01 and missingness <0.05. The covariates included sex at birth, age, age squared, season of wear, variations in Fitbit devices, and the first 16 precalculated genetic PCs (Supplementary Methods). Sex (male/female) was self-reported. Related individuals (kinship score >0.1) had been excluded in the accessed dataset.^59^ The meta-analysis of All of Us and corresponding data of AccPhysAct and AccSed in UKB^39^ (total N = 121,237) was performed using inverse-variance-weighted fixed effects models in METAL (Supplementary Methods).^61^ Following convention, we considered SNPs with p < 5.0 × 10^−8^ as genome-wide significant. We processed the data with cleansumstats pipeline (https://github.com/BioPsyk/cleansumstats), which rendered 11,777,262 SNPs for association analysis.

### Genetic overlap between accelerometer-assessed activity and SMDs

The summary statistics from the meta-analysis of UKB and All of Us data were used to assess if the overall patterns of genetic overlap based on self-reported phenotypes were consistent using LDSC^49^ and bivariate MiXeR (Supplementary Methods).^31^

### Role of funders

The funders had no role in study design, data collection, data analysis, data interpretation, or writing of the report.

## Results

### Genetic architecture

The univariate MiXeR estimates are provided in Table S1. The model fit was acceptable (Table S1, Fig. S1; Supplementary Results), except for SedCom, which was excluded from subsequent MiXeR analyses.

**Fig. 1:**
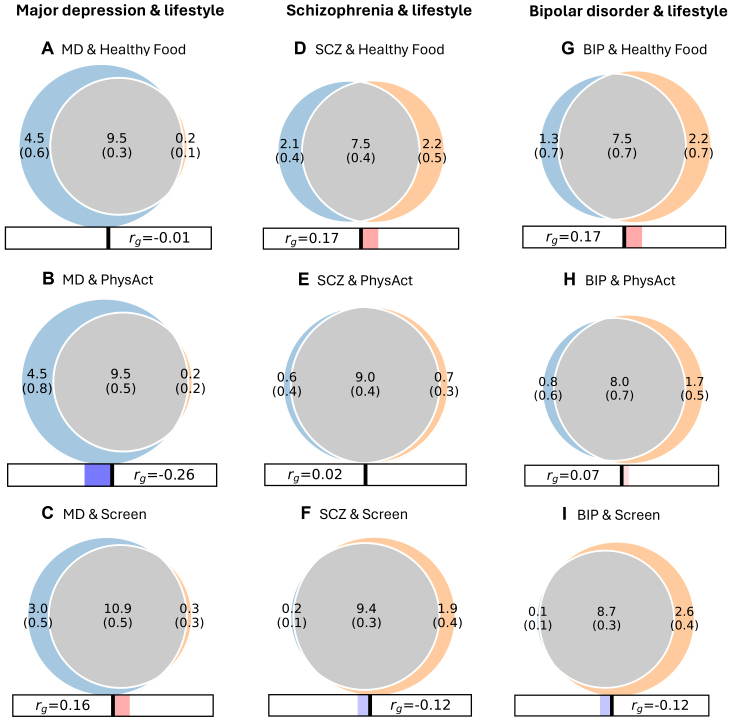
**Venn diagrams of shared and unique genetic variants from bivariate MiXeR.** Venn diagrams of shared and unique trait-influencing variants, showing polygenic overlap (grey) between severe mental disorders (blue) and lifestyle factors (orange). Major depression (MD) exhibits genetic overlap with A) healthy food intake (Healthy Food), B) moderate-to-vigorous physical activity (PhysAct), and C) leisure screen time (Screen). The figure also displays the genetic overlap between schizophrenia (SCZ) and D) Healthy Food, E) PhysAct, and F) Screen. Additionally, the genetic overlap between bipolar disorder (BIP) and G) Healthy Food, H) PhysAct and I) Screen is shown. The numbers in the Venn diagram indicate the estimated quantity of shared and unique trait-influencing variants (in thousands), explaining 90% of SNP heritability in each phenotype, followed by standard error. The size of the circles reflects the degree of polygenicity. The genetic correlations (*r*_*g*_) are also provided.

Bivariate MiXeR analyses indicated widespread genetic overlap between SMDs and lifestyle behaviours (Fig. 1). The majority of variants influencing MD, SCZ, and BIP were estimated to influence Healthy Food, PhysAct, Screen (Fig. 1A–I) and Meat (Figs. S2–S4; Supplementary Results). The SMDs shared fewer SNPs with SedWork, while nearly all the SedWork-influencing variants were shared with SMDs. The Dice coefficients ranged from 48% for MD and SedWork to 93% for SCZ and PhysAct, with a median of 80% (Table 2). All model fits were acceptable (Figs. S2–S4; Table S2; Supplementary Results). In most analyses, the AIC comparison with the maximum-overlap model was negative (Table S2), indicating that the data did not provide sufficient evidence to distinguish the estimated overlap from the theoretical maximum overlap. This situation may arise when the true overlap is close to maximal or when statistical power is insufficient to differentiate between these scenarios (Supplementary Results).^26^

**Table 2:** Bivariate MiXeR and genetic correlations between severe mental disorders and lifestyle factors.

Phenotypes	Dice coefficient (%)		Concordance (%)		Genetic correlation		
	Mean	SD	Mean	SD	*r*_*g*_	SE	p-value
MD & Healthy Food	80.34	2.51	49.50	0.23	−0.01	0.03	0.65
MD & Meat	73.22	3.31	46.02	0.46	−0.11	0.03	4.0e^−4^^a^
MD & PhysAct	80.26	3.57	39.74	0.61	−0.22	0.02	3.6e^−20^^a^
MD & Screen	86.62	2.56	55.93	0.34	0.15	0.02	1.7e^−13^^a^
MD & SedWork	47.80	3.86	49.10	0.79	−0.02	0.04	0.61
SCZ & Healthy Food	77.91	4.46	57.08	0.50	0.15	0.02	1.7e^−10^^a^
SCZ & Meat	85.81	4.46	43.66	0.45	−0.16	0.02	2.9e^−10^^a^
SCZ & PhysAct	93.35	3.30	50.53	0.35	−0.01	0.03	0.68
SCZ & Screen	90.30	2.22	45.89	0.29	−0.10	0.02	1.0e^−7^^a^
SCZ & SedWork	62.39	4.11	48.30	0.41	−0.06	0.03	0.02
BIP & Healthy Food	81.03	7.26	56.75	0.63	0.17	0.03	8.9e^−11^^a^
BIP & Meat	69.21	7.40	46.10	0.60	−0.07	0.03	3.7e^−3^
BIP & PhysAct	86.58	5.88	52.51	0.38	0.08	0.03	1.7 e^−3^^a^
BIP & Screen	86.73	2.08	45.77	0.40	−0.10	0.02	2.2e^−6^^a^
BIP & SedWork	60.82	6.57	53.61	0.58	0.05	0.03	0.15

Dice coefficient (in percentage), i.e., the proportion of shared SNPs between two phenotypes out of the total number of SNPs estimated to influence both phenotypes, estimated with MiXeR. Concordance of variant effects within the shared component. Genetic correlations (r_g_) from LD score regression. MD, major depression; SCZ, schizophrenia; BIP, bipolar disorder; Healthy Food, healthy food intake; Meat, meat consumption; PhysAct, Moderate-to-vigorous intensity physical activity; Screen, leisure screen time; SedWork, sedentary behaviour at work.

a Significant results are demarked with asterisk after Bonferroni correction (2.8e^−3^).

Modest genetic correlations (*r*_*g*_ = −0.22 to 0.17) were estimated with LDSC^49^ (Table 2). MD demonstrated a negative *r*_*g*_ with PhysAct, while a positive *r*_*g*_ with Screen. Both SCZ and BIP had a positive *r*_*g*_ with Healthy Food, and a negative *r*_*g*_ with Screen. BIP also had a positive *r*_*g*_ with PhysAct. The SMDs displayed a negative *r*_*g*_ with Meat. The estimated proportion of shared variants with concordant effects was 40%–57% in the bivariate analyses, supporting a general picture of mixed effect directions. However, the shared SNPs between MD and PhysAct (40%) showed mostly discordant effect directions, in line with the negative *r*_*g*_. The remaining genetic correlations were close to zero and non-significant (Table 2). Since r_*g*_ provides is a genome-wide summary measure of the correlation of effect sizes between two phenotypes, a balanced mixture of genetic variants with concordant and discordant effects will result in a non-significant genetic correlation despite potentially abundant polygenic overlap (Fig. S5A). A negative genetic correlation can also coexist with polygenic overlap if the shared variants captured by MiXeR mainly have discordant effects (Fig. S5B), as previously illustrated.^24^^,^^26^

Trivariate MiXeR indicated most genetic overlap between Healthy Food, Meat, and each of the SMDs (53% for MD, 49% for SCZ, and 46% for BIP). The analyses also revealed considerable genetic overlap between other trios, including PhysAct, Screen, and each of the SMDs (35% for MD, 43% for SCZ and 32% for BIP). In addition, the model estimated a large, shared component between smoking, BMI and both SCZ (44%) and BIP (37%). There was little discrepancy (0–5%) between the trivariate model and the results deduced from three bivariate analyses (Tables S3–S7; Figs. S6–S8; Supplementary Results). The close resemblance between the observed trivariate genetic pattern and the expected overlap based on bivariate analyses under a naïve assumption of a maximum entropy indicates that, once bivariate overlaps are specified, the trivariate overlap is effectively random. This result implies that biological constraints relevant to overlap are predominantly reflected at the bivariate level and the observed trivariate overlap therefore carries little additional systematic biological signal.

### Validation in EAS ancestry samples

The power of the summary statistics from EAS GWASs was insufficient to obtain reliable MiXeR estimates, as indicated by negative AIC values. LDSC estimated positive genetic correlations between BIP and intake of fish (*r*_*g*_ = 0.24) and vegetables (*r*_*g*_ = 0.29), as well as between MD and fish intake (*r*_*g*_ = 0.25); however, the results were only nominally significant after correction for multiple testing (Table S8).

### Shared genetic loci

**Fig. 2:**
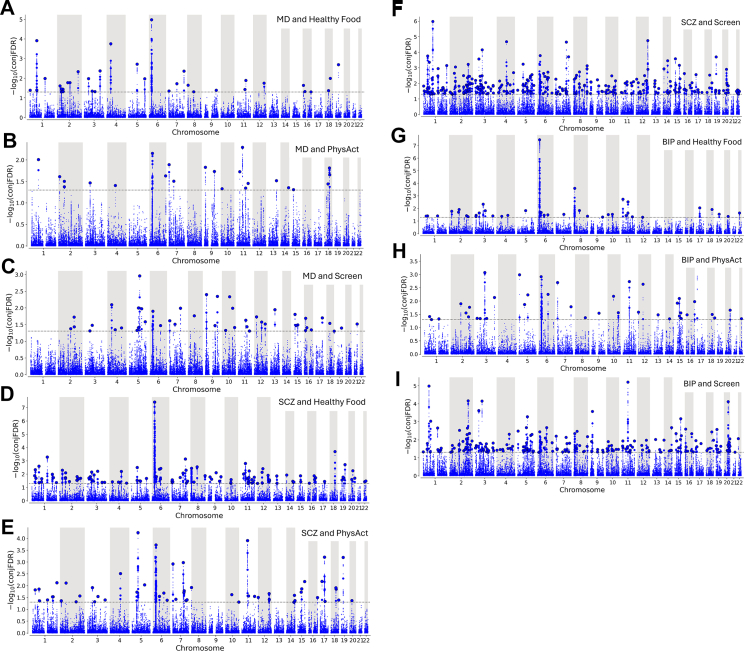
**Common genetic variants jointly associated with severe mental disorders and lifestyle factors at conjFDR <0.05.** Common genetic variants jointly associated with major depression (MD) and A) healthy food intake (Healthy Food), B) moderate-to-vigorous physical activity (PhysAct), and C) leisure screen time (Screen); schizophrenia (SCZ) and D) Healthy Food, E) PhysAct, and F) Screen; and bipolar disorder (BIP) and G) Healthy Food, H) PhysAct, and I) Screen at conjunctional false discovery rate (conjFDR) < 0.05. Manhattan plots showing the –log_10_ transformed conjFDR values for each SNP on the y axis and chromosomal positions along the x axis. SNPs with conjFDR <0.05 (i.e., −log_10_ FDR >1.3) are shown with enlarged data points. A black circle around the enlarged data points indicates the most significant SNP in each LD block. The figure shows the localisation of the ‘conjunctional loci’.

In the conditional Q–Q plots, we observed SNP enrichment for SMDs given their association with lifestyle behaviours, and vice versa, except from SedCom (Figs. S9 and 10). CondFDR identified a series of loci associated with SMDs conditional on lifestyle behaviours (Tables S9–S23A), and vice versa (Tables S9–S23B). At conjFDR <0.05, we identified 8–61 shared loci with MD, 19–258 shared loci with SCZ, and 9–188 shared loci with BIP (Table 3; Fig. 2; Fig. S11). The analyses yielded a total of 551 unique loci jointly associated with SMDs and lifestyle behaviours (Tables S24–S39).

**Table 3:** Shared loci between severe mental disorders and lifestyle factors.

Phenotypes	Shared loci, N	Concordant effects, %
MD & Healthy Food	35	57.1%
MD & Meat	61	37.7%
MD & PhysAct	23	21.7%
MD & Screen	46	69.6%
MD & SedWork	8	62.5%
SCZ & Healthy Food	108	66.7%
SCZ & Meat	67	26.9%
SCZ & PhysAct	54	44.4%
SCZ & Screen	258	40.7%
SCZ & SedWork	19	57.9%
BIP & Healthy Food	39	87.2%
BIP & Meat	26	57.7%
BIP & PhysAct	40	70.0%
BIP & Screen	188	37.2%
BIP & SedWork	9	66.7%

The table lists the number of shared loci at conjunctional false discovery rate <0.05, and the percentage of loci with concordant allelic effect directions. MD, major depression; SCZ, schizophrenia; BIP, bipolar disorder; Healthy Food, healthy food intake; Meat, meat consumption; PhysAct, Moderate-to-vigorous intensity physical activity; Screen, leisure screen time; SedWork, sedentary behaviour at work.

The effect directions of the lead SNPs had variable concordance between SMDs and lifestyle behaviours (Table 3; Tables S24–S38). There were more concordant effect directions for some phenotype pairs (63–87% for MD and Screen and SedWork, SCZ and BIP and Healthy Food, and BIP and PhysAct and SedWork), with more discordant effect directions for other phenotype pairs (22–40% concordance for MD and PhysAct, MD and SCZ and Meat, and SCZ and BIP and Screen). The difference from MiXeR estimates likely stems from the smaller number of shared genetic loci at conjFDR <0.05, whereas MiXeR estimates the total number of shared variants at 90% of SNP-heritability.^31^

### Functional annotation

The mapping of lead SNPs revealed that the number of shared genes between SMDs varied across lifestyle behaviours (Tables S24–S38). MD, SCZ and BIP shared the most genes with Meat (N = 57), Healthy Food (N = 102), and Screen (N = 174), respectively.

We performed gene-set analyses of the mapped genes (Tables S40–S48; Supplementary Results). Genes shared between SCZ and Meat, SCZ and Screen, and BIP and Screen, revealed several synaptic, neuronal, and neurodevelopmental gene-sets. There were no significant gene-set results for MD and individual lifestyle behaviours. Further, we performed enrichment analyses based on genes mapped to the lead SNPs associated with the food intake phenotypes combined as well as physical activity and sedentary behaviours combined, for each SMD. There were several enriched neurodevelopmental gene-sets, including “generation of neurons” and “dendrite development”, associated with the genes mapped for food intake and MD and SCZ (Tables S45 and S46), but none for BIP and food intake. Additionally, the genes mapped to SCZ and food intake were associated with behavioural (e.g., “behaviour”) and synaptic (e.g., “synapse assembly”) gene-sets. The genes shared between physical activity/sedentary behaviours and SCZ and BIP, but not MD, showed enrichment in several gene-sets involved in central nervous system development (e.g., “neurogenesis”), synapse formation (e.g., “synapse organisation”), and cellular processes (e.g., “cell projection organization”) (Tables S47 and S48). There were consistent results from enrichment analyses that included genes in MHC and 8p23.1 (Tables S40–S48).

### SEM

Figs. S12–S14 indicated normally distributed residuals in marginal densities, and symmetric bivariate densities, although for Meat, the distribution was moderately skewed.

### Lifestyle factors as potential mediators in the association between PRS and BMI

The SEM analyses indicated a positive direct association of MD PRS with BMI (β = 0.020–0.024, p < 0.001 [SEM]), while a negative direct association of SCZ PRS (β = −0.024 to −0.022, p < 0.001 [SEM]) and BIP PRS (β = −0.012 to −0.009, p = 1.17e^−8^–1.06e^−5^ [SEM]) with BMI (Figs. S15–S18). Further, the SMD PRSs showed significant associations with the lifestyle factors (Figs. S15–S18; Supplementary Results), which were consistent with the direction of effects observed in the analyses of the GWAS samples. The analyses suggested that part of the association between PRS for SMDs and BMI is mediated by lifestyle. Figs. S15–S18 illustrate the direct and indirect associations with beta estimates. The association between MD PRS and BMI was partly mediated through PhysAct, Screen, and Meat (Figs. S16–S18). The association between SCZ PRS with BMI was partly mediated by Healthy Food, Meat and Screen (Figs. S15 and S16; Fig. S18). Additionally, the association between BIP PRS and BMI was partly mediated by each lifestyle factor (Figs. S15–S18).

### Lifestyle factors as potential mediators in the association between PRS and lipids

The SEM analyses indicated a positive direct association of MD PRS with TG (β = 0.014–0.017, p < 0.001 [SEM]) and a negative direct association with HDL (β = −0.012 to −0.010, p < 0.001 [SEM]). SEM suggested that part of the association between PRS for MD and both lipids is mediated by PhysAct, Screen, and Meat. The analyses indicated a positive direct association between SCZ PRS and HDL (β = 0.015–0.016, p < 0.001 [SEM]), with partial mediation through Healthy Food, Meat, and Screen. SEM demonstrated no direct association between SCZ PRS and TG, but a negative indirect association via Healthy Food, Meat, and Screen. For BIP PRS there was a significant indirect association with lower TG and increased HDL through each lifestyle factor (Supplementary Results).

### Smoking as a potential mediator in the association between SCZ and BIP PRS and BMI

SEM indicated a negative association between smoking and BMI (β = −0.105; 95% CI, −0.119 to −0.091, p < 0.001 [SEM]). There was an indirect association between SCZ PRS and BMI via smoking, while no significant indirect association was found for BIP PRS (Supplementary Results).

### Potential causal relationships supported by MR analyses

MR analyses indicated potential causal relationships, but consensus across the four MR methods was lacking (Tables S49 and S50; Supplementary Results): Screen showed a positive effect on MD risk and a negative effect on BIP, and Healthy Food showed a positive effect on BIP risk according to at least two methods. In addition, a causal effect from SCZ to less Meat and more Healthy Food was suggested (Tables S49 and S50; Supplementary Results).

### Loci associated with accelerometer-assessed activity

The GWAS in All of Us identified one genome-wide significant locus (p < 5 ×10^−8^) [PLINK] associated with AccSed, while there was no significant locus for AccPhysAct (Table S51). In the meta-analyses, three and seven loci reached genome-wide significance for AccPhysAct and AccSed, respectively (Table S52). One of the loci for AccPhysAct and five of the loci for AccSed were not identified in the original UKB GWAS.^39^ The genetic correlations between AccPhysAct, AccSed and self-reported phenotypes are provided in Table S53 (see also Supplementary Results).

### Genetic overlap between accelerometer-assessed activity and SMDs

The univariate MiXeR analyses demonstrated acceptable model fit with positive AICs (Table S54; Fig. S19; Supplementary Results). Bivariate MiXeR estimated that the majority of variants influencing SMDs overlap with those influencing AccPhysAct and AccSed (Figs. S20–S22). The Dice coefficients ranged from 74% to 90%. The estimated overlap was close to complete (Table S56), and MiXeR was unable to differentiate the modelled overlap from maximum possible overlap except for the pair BIP and AccSed (Table S55; Figs. S20–S22; Supplementary Results).

In line with the analyses based on self-reported phenotypes, there were negative genetic correlations between MD and AccPhysAct (*r*_*g*_ = −0.12), SCZ & AccSed (*r*_*g*_ = −0.09), and BIP and AccSed (*r*_*g*_ = −0.07), while the latter was not significant after correction for multiple testing (Table S56).

## Discussion

Using a comprehensive approach, we show considerable genetic overlap between SMDs and self-reported lifestyle behaviours (food intake, physical activity and sedentary behaviour), with different patterns of overlap across the disorders. MD was genetically correlated with less healthy lifestyle behaviours, while SCZ and BIP displayed genetic correlations with healthier lifestyle factors. A similar pattern of divergent findings across SMDs were found for objectively measured accelerometer activity. This observation was also evident in the different effect directions of the 551 distinct shared loci. The genetic associations between SMDs and BMI and lipids were in part mediated by lifestyle behaviours. The divergent patterns of genetic relationships with lifestyle factors across SMDs provide insights into the underlying biological pathways, which seem to mediate the relationship between SMDs, BMI, and lipids.

Bivariate and trivariate MiXeR revealed extensive genetic overlap despite weak genetic correlations between SMDs and lifestyle factors. An overall pattern emerged suggesting that SCZ and BIP have a genetic propensity towards healthier lifestyle behaviours (higher levels of Healthy Food and PhysAct and lower Screen score), while MD is genetically associated with unhealthier lifestyle (less PhysAct and higher Screen score). The findings corroborate recent evidence of a negative genetic correlation between MD and exercise based on self-report and accelerometer-measured activity.^15^^,^^62^ Previous findings for SCZ and BIP are inconsistent,^16^^,^^18^, ^19^, ^20^, ^21^^,^^63^ and the genetic relationship between SMDs and lifestyle factors may differ across populations. The genetic link with more Healthy Food and less Meat in SCZ and BIP may indicate a propensity towards a more plant and fish-based diet, consistent with some other studies.^21^^,^^64^ In EAS ancestry samples, we also found that BIP is genetically correlated with healthier dietary intake. However, the negative genetic correlation with Meat across SMDs was not replicated in the EAS cohorts, in line with a prior study of EAS samples.^63^

The results for BMI and lipids align with the genetic relationship between lifestyle and SMDs. Genetic liability to MD is associated with higher BMI and TG and lower HDL, while the opposite direction was found for SCZ and BIP, consistent with other studies.^13^^,^^15^^,^^17^^,^^23^ This is supported by our recent work in lipidomics, showing a divergent pattern of genetic relationships between SMDs and a range of metabolic markers.^65^ Further, we found that part of the associations between genetic risk for SMDs, BMI, and lipid levels appears to be mediated by these lifestyle behaviours involving food intake and physical activity. In addition, a small part of the negative genetic association with lower BMI in SCZ may be mediated by a genetic propensity to smoking. Although the genetic effects are modest, the findings may be useful for development of risk prediction and facilitate targeted interventions (i.e., precision medicine). The role of genetic susceptibility and interaction with lifestyle behaviours may also inform design of public health programs for cardiometabolic health, taking mental disorders, particularly MD, into account.

Interestingly, the genetic findings for SCZ and BIP are incongruent with clinical and epidemiological observations of higher BMI, dyslipidemia, and unhealthy lifestyle factors in these patient populations.^1^^,^^3^^,^^4^ Still, there are indications of lower BMI before onset of SCZ in longitudinal studies,^66^ and greater lipid disturbances and more unhealthy lifestyle as the illness progress.^67^, ^68^, ^69^ Thus, our findings indicate that factors beyond common genetic variants are involved, such as metabolic side-effects of psychotropic medications, cognition, symptoms (e.g., fatigue and amotivation), and socioeconomic challenges.^4^^,^^6^ Longitudinal data indicate that patients with SCZ treated with antipsychotics display more weight gain than controls and untreated individuals, with the latter group maintaining a lower BMI trajectory than controls.^70^ Accordingly, the adverse effects of antipsychotics probably outweigh the genetic predisposition to lower BMI at the phenotypic level. These medications may also mask or override genetic effects on lipids and lifestyle factors,^4^^,^^71^ while further research is necessary to clarify the influence of the medications in conjunction with other modifiable factors and genetic risk.

Our results in MD are in alignment with clinical and epidemiology data for CVD risk factors^72^ and unhealthy lifestyle behaviours^4^ in MD. Thus, genetic drivers of unhealthy lifestyle seem to work in concert with genetic factors in MD. Moreover, the results are consistent with other data indicating that regular exercise is associated with lower MD risk.^4^ However, we cannot rule out that some of the genetic overlap may be influenced by SNPs associated with depressive symptoms (e.g., fatigue and reduced motivation) that manifest as reduced activity.

The overall pattern of SCZ and BIP being genetically associated with healthier lifestyle behaviours, while MD displayed the opposite, also emerged in the MR analyses. However, caution is warranted when interpreting MR results for complex psychiatric disorders and lifestyle traits.^73^ The lack of robust findings across MR methods applied in this study and mixed evidence in previous studies,^18^^,^^19^^,^^21^^,^^74^ prevent us from drawing robust causal inferences.

We identified mixed effect directions among the shared loci between SMDs and lifestyle behaviours. The considerable genetic pleiotropy with varying concordance rates may be compatible with the presence of subgroups of patients with a higher genetic propensity towards both SMDs and unhealthy lifestyle behaviours, as suggested for CVD comorbidity.^13^^,^^22^ For example, subgroups with a higher CVD risk may include atypical depression,^75^ BIP with more depressive symptoms,^76^ and SCZ with more severe negative symptoms such as amotivation,^77^ which are also linked to lifestyle.^77^^,^^78^ Currently, clinical evidence supporting this subgroup hypothesis is more apparent for MD: Individuals with atypical depressive symptoms, are also more likely to experience metabolic disturbances and immunological dysregulation, making them more at risk for CVD.^78^ This clustering of symptoms and immune-metabolic dysregulation is often accompanied by emotional eating and reduced physical activity.^78^^,^^79^ Moreover, studies indicate a shared genetic basis for the atypical depressive symptoms and immuno-metabolic dysregulation.^78^ Further characterisation of the underlying biological and genetic mechanisms is necessary, which may lead to new treatment options for this subtype.^78^

While the biological implication of the findings of the current study is complex, genetic loci associated with physical activity are expressed in brain regions of the reward system and dopaminergic neurons^16^ involved in motivation and processing emotions. The dysregulation of dopamine signalling in the reward circuit is also implicated in SMDs^24^^,^^30^^,^^80^ and represent a plausible neurobiological pathway shared between MD and physical inactivity. Further, our finding of a modest negative genetic correlation between SMDs and Meat aligns with some genetic studies,^64^^,^^74^ while epidemiological findings are inconclusive.^81^ Meat is a source of proteins and other nutrients important for maintaining the central nervous system and regulating mood and cognition.^82^ However, processed red meat contains saturated fats, which influence cardiometabolic health^35^ and may impair brain functioning.^82^

Functional analyses of the shared loci between SMDs and lifestyle factors implicated the involvement of biological processes associated with neurodevelopment, neuronal and synaptic properties. The results are in agreement with the neurodevelopmental hypothesis of SCZ,^24^ and brain dysfunction being implicated in the pathophysiology of BIP and MD.^80^ In addition, brain function regulates lifestyle behaviours,^83^ which in turn influence CVD risk. The results expand on prior findings of genetic overlap between SMDs and CVD risk implicating brain-expressed genes.^13^^,^^17^^,^^22^ Furthermore, the neuronal and synaptic processes associated with genes shared with lifestyle behaviours align with overlapping neuronal functions regulating lifestyle behaviours.^83^ Moreover, while the shared loci were implicated in neurobiological processes, the biological processes may have mixed effect directions across SMDs. Biological processes and pathways are likely to interact with the environment (e.g., antipsychotics and stressors) to drive the phenotypic associations between SMDs and unhealthy lifestyle within subgroups.^84^ However, experimental studies are needed to clarify the underlying mechanisms.

The current study has some limitations. The lifestyle GWASs relied on self-reported behaviours, which may be influenced by emotional states and cognitive biases (e.g., recall bias).^16^^,^^28^ Awareness of the benefits of physical activity and certain food items can also influence the accuracy of self-report and contribute to overreporting of healthy behaviours. In addition, the food categories are coarse and do not capture relevant nuances in diets. To mitigate the limitations of self-reported physical activity, we performed analyses with accelerometer-assessed activity supporting the validity of the main findings. Further, the UKB participants are healthier with higher socioeconomic status than the general population.^85^ This healthy volunteer bias is likely to influence the lifestyle towards more beneficial behaviours. While this can increase the likelihood of type II errors, the direction of effects is likely to be generalisable.^85^ Moreover, the GWASs were performed in European populations, with limited ancestral diversity. Thus, the results may not be generalisable across other ancestries and cultures with different lifestyle practices. Other imitations include the SEM and MR methods, which provide uncertain evidence for causal inferences.^27^^,^^73^ Drawing causal inferences from MR is precluded by the complexity of the psychiatric and behavioural phenotypes and the high risk of horizontal pleiotropy.^73^ Furthermore, while conjFDR is useful for identifying shared genetic loci, the tool cannot pinpoint the causal variants.^25^ The gene mapping approach^33^ and enrichment analyses^32^ have limited accuracy in determining the causal variants and biological processes, respectively. Finally, we lacked data on illness severity, symptoms and medication use. As GWAS cohorts increase, deep phenotyping of participants will enable the investigation of potential subgroups, clinical characteristics, and their relationship with lifestyle factors.

In conclusion, by applying a comprehensive statistical approach, we uncovered divergent patterns of genetic overlap between SMDs and lifestyle behaviours. While MD was associated with a genetic propensity towards unhealthy lifestyle behaviours, the opposite trend was found for SCZ and BIP, implicating the importance of modifiable factors. Furthermore, lifestyle behaviours seem to partly mediate the relationship between genetic risk for SMDs, BMI, and lipids. Moreover, the findings also indicated subgroups of SMDs with higher genetic predispositions to unhealthy lifestyle behaviours. The findings have implications for understanding the interplay between SMDs and lifestyle, and will support more targeted lifestyle interventions and individualised treatment to prevent CVD in SMDs.

## Contributors

LR and OAA conceived the study. LR, ZR, NP and OAA were involved in study design. The All of Us data was accessed and verified by ZR and AS, and LR, ZR and AS accessed and verified all the other underlying data. ZR conducted most of the statistical analyses, and LR conducted some analyses, with methodological input from AS, NP, PP and OAA. All authors had full access to the data if they wished and contributed to interpretation of results. LR wrote the original manuscript draft. All authors contributed to the review and editing of the manuscript. All authors read and approved the final version of the manuscript.

## Data sharing statement

The summary statistics from the Psychiatric Genomics Consortium analysed in the current study can be downloaded here: https://www.med.unc.edu/pgc/download-results/, and the subsample of MD from 23andMe can be requested here: https://www.23andme.com. 23andMe summary statistics are made available through 23andMe to qualified researchers under an agreement with 23andMe that protects the privacy of 23andMe participants. Please visit https://research.23andme.com/dataset-access/ for more information. The lifestyle, BMI, and smoking datasets are available in the following repositories of GWASs:

Food intake: https://www.ebi.ac.uk/gwas/publications/35653391.

Self-reported physical activity and sedentary behaviours: https://www.ebi.ac.uk/gwas/publications/36071172.

Accelerometer-assessed physical activity and sedentary behaviour from UKB: https://doi.org/10.5287/bodleian:yJp6zZmdj.

BMI: https://portals.broadinstitute.org/collaboration/giant/index.php/GIANT consortium data files.

Smoking: https://conservancy.umn.edu/items/ca7ed549-636b-41c0-ae79-97c57e266417.

Summary statistics from East-Asian samples are available in the following repositories:

MD: https://figshare.com/articles/dataset/mdd2021asi/16989442.

SCZ: https://doi.org/10.6084/m9.figshare.19193084.v1.

BIP: https://figshare.com/articles/dataset/bip2024/27216117?file=49760769.

Dietary factors: https://humandbs.dbcls.jp/en/hum0014-v37.

Individual-level data from the UKB was obtained through application (27412) to the UKB (www.ukbiobank.ac.uk) and is based on approval from the UK Biobank Access Committee. Analyses must be conducted on the UK Biobank Research Analysis Platform (UKB-RAP). Access to individual-level data from the All of Us Research Program: Controlled Tier Dataset version 8 is available to authorised users here: https://www.researchallofus.org/data-tools/workbench/ after registration (https://workbench.researchallofus.org/).

Summary statistics generated for accelerometer-based activity from All of Us and UKB meta-analysis have been uploaded to Zenodo, with the following link https://doi.org/10.5281/zenodo.19980833.

Codes used for carrying out the analyses are available here:

Cond/conjFDR: https://github.com/precimed/pleiofdr.

Univariate and bivariate MiXeR: https://github.com/precimed/mixer.

Trivariate MiXeR: https://github.com/precimed/mix3.

LDSC: https://github.com/bulik/ldsc.

FUMA: https://fuma.ctglab.nl/

Open Targets Genetics: https://genetics.opentargets.org/

PRS: https://choishingwan.github.io/PRSice/

SEM: https://github.com/yrosseel/lavaan.

MR: https://github.com/MRCIEU/TwoSampleMR.

GWAS: https://www.cog-genomics.org/plink/2.0/

Meta-analysis: https://github.com/statgen/METAL.

## Declaration of interests

Dr. Anders M. Dale is a Founding Director and holds equity in CorTechs Labs, Inc. (DBA Cortechs.ai), Precision Pro, Inc., Precision Health AS, Precision Health and Wellness, Inc., and Diploid Genomics, Inc. Dr. Dale is the President and a Board of Trustees member of the J. Craig Venter Institute (JCVI) and holds an appointment as Professor II at the University of Oslo in Norway. Dr. Andreassen has received speaker fees from Lundbeck, Lily, BMS, Janssen, Otsuka, and Sunovion and is a consultant to and holds equity in Cortechs.ai., Precision Health, and Ledidi. He is also a member of the Scientific Advisory Board of Ledidi and The Health Portfolio Board of Research Council of Norway. Dr. Frei is a consultant to Precision Health. Pravesh Parekh reports support from grants from the National Institutes of Health, Wellcome Leap via the CARE program, European Union's Horizon 2020 research and innovation programme under the Marie Skłodowska-Curie grant, and the Research Council of Norway, in addition to support for attending meetings and/or travel from International Society for Psychiatric Genetics, Organization for Human Brain Mapping, and the Society for Biological Psychiatry. Nadine Parker also reports support for attending meetings and/or travel from International Society for Psychiatric Genetics and the Society for Biological Psychiatry. The other authors report no potential competing interests.
