# Supplementary material for: Evidence for overlapping genetic architecture between lifestyle factors and severe mental disorders with different patterns across diagnoses
Source: eBioMedicine. 2026 May 27;128:106304. doi: 10.1016/j.ebiom.2026.106304 (PMC13233568; doi:10.1016/j.ebiom.2026.106304)
Supplement: Supplementary Material [file mmc1.docx]

**Supplementary Material for**

**Evidence for overlapping genetic architecture between lifestyle factors and severe mental disorders with different patterns across diagnoses**

Linn Rødevand, Zillur Rahman, Piotr Jaholkowski, Nadine Parker, Unnur A. Valdimarsdóttir, Olav B. Smeland, Markos Tesfaye, Pravesh Parekh, Oleksandr Frei, Srdjan Djurovic, Nils Eiel Steen, Anders M. Dale, Alexey Shadrin, Ole A. Andreassen.

**Content**

[Supplementary Methods 3](#_Toc228786770)

[Statistical analyses 3](#_Toc228786771)

[MiXeR analysis 3](#_Toc228786772)

[Conditional Q-Q plots 8](#_Toc228786773)

[Conditional and Conjunctional False Discovery Rate 10](#_Toc228786774)

[Genomic loci definition 12](#_Toc228786775)

[Effect sizes and genetic correlations 12](#_Toc228786776)

[Validation in EAS ancestry samples 12](#_Toc228786777)

[Functional annotation 13](#_Toc228786778)

[Structural Equation Modeling 14](#_Toc228786779)

[Mendelian Randomization 15](#_Toc228786780)

[Genome-wide analyses of accelerometer-assessed activity 16](#_Toc228786781)

[Genetic overlap between accelerometer-assessed activity and SMDs 18](#_Toc228786782)

[Supplementary Results 18](#_Toc228786783)

[MiXeR estimates 18](#_Toc228786784)

[Cross-trait enrichment 21](#_Toc228786785)

[Conditional False Discovery Rate results 21](#_Toc228786786)

[Conjunctional False Discovery Rate results 21](#_Toc228786787)

[Functional annotation 22](#_Toc228786788)

[Mediation analyses with SEM 23](#_Toc228786789)

[Mediation analyses of direct and indirect associations between PRS for SMDs and BMI via lifestyle behaviours 23](#_Toc228786790)

[Mediation analyses of direct and indirection associations between PRS for SMDs and lipids via lifestyle behaviours 25](#_Toc228786791)

[Mediation analyses of direct and indirection associations between PRS for SCZ and BIP and BMI via smoking 26](#_Toc228786792)

[MR 26](#_Toc228786793)

[Loci associated with accelerometer-assessed activity 26](#_Toc228786794)

[Genetic overlap between accelerometer-assessed activity and SMDs 27](#_Toc228786795)

[Supplementary Figures 29](#_Toc228786796)

Supplementary Methods

Statistical analyses

MiXeR analysis

We used the causal mixture model (MiXeR)^2, 3^ to estimate the unique and shared genetic architecture between the phenotypes (https://github.com/precimed/mixer). MiXeR infers characteristics of the genetic architecture of complex phenotypes based on GWAS summary statistics using Gaussian mixture models, which assumes that a given data set can be modeled as a “mixture” of predefined components, each with their own Gaussian (normal) distribution. First, MiXeR constructs a univariate mixture model for each phenotype. For each SNP, $i$, univariate MiXeR models its additive genetic effect of allele substitution,$\beta_{i}$, as a point-normal mixture, $\beta_{i}=\left( 1-\pi_{1} \right)N\left( 0,0 \right)+\pi_{1}N(0, \sigma_{\beta}^{2})$, where $\pi_{1}$ represents the proportion of non-null SNPs (`polygenicity`) and $\sigma_{\beta}^{2}$ represents variance of effect sizes of non-null SNPs (`discoverability`). Then, for each SNP, $j$, MiXeR incorporates LD information and allele frequencies for SNPs extracted from 1000 Genomes Phase3 data by LD score regression software^3, 4^, and estimates the expected probability distribution of the signed test statistic, $z_{j}=\delta_{j}+\epsilon_{j}=N\sum_{i} \sqrt{H_{i}}r_{ij}\beta_{i}+\epsilon_{j}$, where $N$ is sample size, $H_{i}$ indicates heterozygosity of i-th SNP, $r_{ij}$ indicates allelic correlation between i-th and j-th SNPs, and $\epsilon_{j}\sim N(0, \sigma_{0}^{2})$ is the residual variance. Further, the three parameters, $\pi_{1}, \sigma_{\beta}^{2}, \sigma_{0}^{2}$, are fitted by direct maximization of the likelihood function. The number of trait-influencing variants (i.e., variants with pure genetic effects not induced by LD) is estimated as $M\pi_{1}$, where M is the number of SNPs in the reference panel. The phenotypic variance explained on average by a trait-influencing variant is calculated as $\bar{H}\sigma_{\beta}^{2},$ in which $\bar{H}$ is the average heterozygosity across SNPs in the reference panel. As SNP-based heritability is a function of a trait’s polygenicity and discoverability, this parameter is calculated as $h_{SNP}^{2}=M\pi_{1}\times\bar{H}\sigma_{\beta}^{2}$^2^.

MiXeR is extended to bivariate analyses to calculate the shared genetic architecture between two traits. The bivariate MiXeR model assumes that the additive genetic effects of a given variant on two traits can be described as a mixture of four components, including 1) null SNPs in both traits ($\pi_{0})$, 2) SNPs with a unique effect on the first trait ($\pi_{1}$), 3) SNPs with a unique effect on the second trait ($\pi_{2}$), 4) and SNPs with non-zero effect on both traits ($\pi_{12}$). Furthermore, MiXeR models variance-covariance matrix as $\boldsymbol{\Sigma}_{\mathbf{12}}=\left[ \begin{matrix} \sigma_{1}^{2} & {\rho_{12}\sigma}_{1}\sigma_{2} \\ {\rho_{12}\sigma}_{1}\sigma_{2} & \sigma_{2}^{2} \end{matrix} \right]$ where $\rho_{12}$ indicates correlation of effect sizes within the shared component, and $\sigma_{1}^{2}$ and $\sigma_{2}^{2}$ correspond to the discoverability parameter estimated in the univariate analysis of the two traits. After fitting parameters of the model, genetic correlation is calculated as $r_{g}=\frac{\rho_{12}\pi_{12}}{\sqrt{(\pi_{1}+\pi_{12})(\pi_{2}+\pi_{12})}}.$ For details, see ^2^. After estimating the size of the shared and unique components, MiXeR calculated the Dice coefficient ~~(DC)~~ to estimate the overall extent of genetic overlap with the formula Dice coefficient = $\frac{2\pi_{12}}{{\pi_{1}+\pi_{2}+2\pi}_{12}}$.

To test the reliability of the model parameters, i.e., the ability of the MiXeR to accurately predict the actual GWAS data, we calculated 1) Akaike information criterion (AIC) values, and constructed 2) modelled vs. actual conditional Q-Q plots, and 3) negative log-likelihood curves. Both AIC values and Q-Q plots were generated for univariate and bivariate MiXeR. To identify analyses with insufficiently powered GWAS summary statistics, we used AIC ($2k-2\ln L$), where $k$ is the number of free parameters in the model, $L$ is the value of the likelihood function, and $n$ is the effective number of SNPs used in optimization procedure. In *univariate* analyses, AIC denotes the difference between AIC of the model fitted using MiXeR and AIC of the infinitesimal model, which assumes that all variants are non-null yielding the constrained univariate model with 2 free parameters ($\sigma_{\beta}^{2}, \sigma_{0}^{2}$) and fixing polygenicity parameter equal to 1. A positive AIC value implies that the GWAS sample is sufficiently powered to separate the model fitted using MiXeR from the infinitesimal model.

For *bivariate* analyses, we calculated the difference between *AIC* for the full bivariate model, $k=3$, and AIC for the reduced bivariate model, $k=2$, due to $\pi_{12}$ being constrained to smallest or largest possible value ( $\pi_{12}^{min}=r_{g}\sqrt{\pi_{1}^{u} \pi_{2}^{u}}$ and $\pi_{12}^{max}=min(\pi_{1}^{u}, \pi_{2}^{u})$), respectively. Each bivariate MiXeR analyses provides two AIC values, including AIC_best_vs_min_ (which compares MiXeR modelled fit with a constrained model with minimal polygenic overlap) and AIC_best_vs_max_ (which compares MiXeR modelled fit with a constrained model with maximum polygenic overlap). Accordingly, AIC differences for bivariate MiXeR are calculated by comparing the best-fitting model to minimum possible overlap (constrained by genetic correlation) and maximum possible overlap (constrained by the polygenicity of the least polygenic trait). Two positive values of AIC indicates that GWAS summary statistics have enough information to distinguish the custom polygenic overlap, as shown on the MiXeR Venn diagrams, from the constrained models with minimal ($\pi_{12}^{min}$) and maximum ($\pi_{12}^{max}$) polygenic overlap. A positive AIC_best_vs_min_ while a negative AIC_best_vs_max_ imply genetic overlap beyond the minimal polygenic overlap and that the MiXeR estimates are indistinguishable from complete overlap. Moreover, this scenario can be interpreted as extensive genetic overlap and that more powerful datasets are likely to improve the estimates given the proximity to complete overlap.^1^

Furthermore, parameter optimization for the number of shared SNPs was illustrated in log-likelihood curves, which plot the negative log-likelihood function (y axis) against the modelled number of shared variants (x axis) (Figs. S2-4).^2^ The “best” model of polygenic overlap is represented as the lowest point (i.e. the lowest negative log-likelihood) on the curve. Minimum and maximum possible overlap were represented as the minimum (left) and maximum (right) values plotted along the x-axis (number of variants) on the curve, which were compared with the MiXeR-modelled estimates in the AIC tests. A positive AIC difference compared to minimum overlap is visualized by a descending curve from the minimum number of shared variants to the lowest point. A positive AIC difference compared to maximum overlap is illustrated by an ascending curve to the maximum number of shared variants.

The *univariate* Q-Q plots show the distribution of expected p values under a null model (no SNPs associated with the phenotype) (x axis) versus observed p values (y axis) (Fig. S1). Univariate Q-Q plots illustrate whether the univariate MiXeR estimates provide accurate estimates of the data plots. The conditional Q-Q plots for *bivariate* analyses illustrate observed versus expected −log_10_ p values in the primary trait as a function of the significance of association with a secondary trait at the level of p ≤ 0.1, p ≤ 0.01, and p ≤ 0.001, with successive leftward deflections of SNP strata with higher significance in the secondary trait (see middle plots in Figs. S2-4). Accurate MiXeR estimates are indicated if the data Q-Q plots (solid lines) are closely reproduced by the model predictions (dashed lines) across all p value strata.^2^

Finally, we used *trivariate* MiXeR (<https://github.com/precimed/mix3>),^5^ an extension of bivariate MiXeR to investigate genetic overlap between three phenotypes. For a given set of three bivariate overlaps and without any prior knowledge, the maximum entropy distribution of the trivariate overlap can be conceptualized as the central point among all possible trivariate overlap distributions.^5^ Reconstructing trivariate overlap from these three bivariate analyses under the assumption of maximum entropy can result in erroneous estimates.^5^ Trivariate MiXeR assesses if the pattern of genetic overlap among three phenotypes differs from the overlap patterns expected based on three bivariate overlaps under the naïve assumption of maximum entropy. This statistical approach for trivariate overlap preserves the basic assumptions of the bivariate MiXeR model. The model adopts an additive genetic effects framework. In the univariate analysis, the direct (not induced by LD) effect $\beta_{j}$ of the *j*^th^ variant on a phenotype is modeled as a mixture of null and phenotype-influencing components characterized by two parameters: the proportion of variants influencing the phenotype (polygenicity, $\pi\in[0,1]$) and the variance of their effect sizes (discoverability, $\sigma^{2}$):

$\beta_{j}=\left\{ \begin{aligned} 0, &1-\pi\\ N\left( 0,\sigma^{2} \right), & \pi\end{aligned} \right.$

where $N\left( 0,\sigma^{2} \right)$ is a normal distribution with zero mean and $\sigma^{2}$ variance.

In an analysis of the three phenotypes ($i=1, 2, 3$), some variants may affect all three phenotypes ($\pi_{123}$), other variants may affect a pair of phenotypes but not the third phenotype ($\pi_{12}$, $\pi_{13}$, $\pi_{23}$), and some variants might be phenotype-specific ($\pi_{1},\pi_{2},\pi_{3}$). Most variants are expected to have no effect on any phenotype ($\pi_{0}=1-\pi_{1}-\pi_{2}-\pi_{3}-\pi_{12}-\pi_{13}-\pi_{23}-\pi_{123}$). We assume that the variants for a given phenotype follow the same distribution of effect sizes (with corresponding discoverabilities $\sigma_{1}^{2}$, $\sigma_{2}^{2}$, $\sigma_{3}^{2}$), irrespective of their effects on the other two phenotypes. Genetic correlations are modeled by introducing correlations of effect sizes within each of three pairwise overlaps ($\rho_{12},\rho_{13},\rho_{23}$). Under these assumptions, the trivariate distribution of direct effects of the j^th^ variant is modeled as a mixture of eight components:

$\left( \begin{matrix} \beta_{1j} \\ \beta_{2j} \\ \beta_{3j} \end{matrix} \right)=\left\{ \begin{aligned} \begin{aligned} \bar{0}, &\pi_{0} \\ N\left( \bar{0},\boldsymbol{\Sigma}_{1} \right), &\pi_{1} \end{aligned} \\ \begin{matrix} \begin{aligned} N\left( \bar{0},\boldsymbol{\Sigma}_{2} \right), &\pi_{2} \\ N\left( \bar{0},\boldsymbol{\Sigma}_{3} \right), &\pi_{3} \end{aligned} \\ \begin{aligned} N\left( \bar{0},\boldsymbol{\Sigma}_{12} \right), &\pi_{12} \\ N\left( \bar{0},\boldsymbol{\Sigma}_{13} \right), &\pi_{13} \end{aligned} \\ \begin{aligned} N\left( \bar{0},\boldsymbol{\Sigma}_{23} \right), &\pi_{23} \\ N\left( \bar{0},\boldsymbol{\Sigma}_{123} \right), &\pi_{123} \end{aligned} \end{matrix} \end{aligned} \right.$

where $\bar{0}=\left( \begin{matrix} 0 \\ 0 \\ 0 \end{matrix} \right)$ and $\boldsymbol{\Sigma}_{1}\boldsymbol{=}\left( \begin{matrix} \sigma_{1}^{2} & 0 & 0 \\ 0 & 0 & 0 \\ 0 & 0 & 0 \end{matrix} \right)$, $\boldsymbol{\Sigma}_{2}\boldsymbol{=}\left( \begin{matrix} 0 & 0 & 0 \\ 0 & \sigma_{2}^{2} & 0 \\ 0 & 0 & 0 \end{matrix} \right)$, $\boldsymbol{\Sigma}_{3}\boldsymbol{=}\left( \begin{matrix} 0 & 0 & 0 \\ 0 & 0 & 0 \\ 0 & 0 & \sigma_{3}^{2} \end{matrix} \right)$,

$\boldsymbol{\Sigma}_{12}\boldsymbol{=}\left( \begin{matrix} \sigma_{1}^{2} & \rho_{12}\sigma_{1}\sigma_{2} & 0 \\ \rho_{12}\sigma_{1}\sigma_{2} & \sigma_{2}^{2} & 0 \\ 0 & 0 & 0 \end{matrix} \right)$, $\boldsymbol{\Sigma}_{13}\boldsymbol{=}\left( \begin{matrix} \sigma_{1}^{2} & 0 & \rho_{13}\sigma_{1}\sigma_{3} \\ 0 & 0 & 0 \\ \rho_{13}\sigma_{1}\sigma_{3} & 0 & \sigma_{3}^{2} \end{matrix} \right)$, $\boldsymbol{\Sigma}_{23}\boldsymbol{=}\left( \begin{matrix} 0 & 0 & 0 \\ 0 & \sigma_{2}^{2} & \rho_{23}\sigma_{2}\sigma_{3} \\ 0 & \rho_{23}\sigma_{2}\sigma_{3} & \sigma_{3}^{2} \end{matrix} \right)$,

$\boldsymbol{\Sigma}_{123}\boldsymbol{=}\left( \begin{matrix} \sigma_{1}^{2} & \rho_{12}\sigma_{1}\sigma_{2} & \rho_{13}\sigma_{1}\sigma_{3} \\ \rho_{12}\sigma_{1}\sigma_{2} & \sigma_{2}^{2} & \rho_{23}\sigma_{2}\sigma_{3} \\ \rho_{13}\sigma_{1}\sigma_{3} & \rho_{23}\sigma_{2}\sigma_{3} & \sigma_{3}^{2} \end{matrix} \right)$ are covariance matrices of multivariate normal distributions corresponding to the different phenotype-influencing components**.**

The joint signed association test statistics (z-score) of the j^th^ variant is then determined by:

$\left( \begin{matrix} z_{1j} \\ z_{2j} \\ z_{3j} \end{matrix} \right)=\sum_{k=1}^{M} \sqrt{h_{k}}r_{jk}\left( \begin{matrix} \sqrt{N_{1j}}\beta_{1k} \\ {\sqrt{N_{2j}}\beta}_{2k} \\ {\sqrt{N_{3j}}\beta}_{3k} \end{matrix} \right)+\epsilon$

where $N_{ij}$ ($i=1, 2, 3$) is the sample size of the GWAS for the i^th^ phenotype and j^th^ variant, $h_{k}$ is the heterozygosity of variant *k*, $M$ is the number of variants in LD with the variant *k*, $r_{jk}$ is the Pearson’s correlation coefficient between the genotypes of the j^th^ and k^th^ variants (quantifying LD), and $\epsilon\sim N\left( \bar{0},\boldsymbol{\Sigma}_{0} \right)$ is a normally distributed vector of residuals with covariance matrix

$\boldsymbol{\Sigma}_{0}\boldsymbol{=}\left( \begin{matrix} \sigma_{01}^{2} & \rho_{012}\sigma_{01}\sigma_{02} & \rho_{013}\sigma_{01}\sigma_{03} \\ \rho_{012}\sigma_{01}\sigma_{02} & \sigma_{02}^{2} & \rho_{023}\sigma_{02}\sigma_{03} \\ \rho_{013}\sigma_{01}\sigma_{03} & \rho_{023}\sigma_{02}\sigma_{03} & \sigma_{03}^{2} \end{matrix} \right)$,

where $\sigma_{0i}^{2}$ ($i=1, 2, 3$) is a residual variance of the i^th^ phenotype and $\rho_{0ij}$ ($i,j=1, 2, 3$) is a correlation between residuals of the i^th^ and j^th^ phenotypes. To estimate the nineteen parameters of the model $\left( \pi_{1},\pi_{2},\pi_{3},\pi_{12},\pi_{13},\pi_{23},\pi_{123},\sigma_{1},\sigma_{2},\sigma_{3},\sigma_{01},\sigma_{02},\sigma_{03},\rho_{12},\rho_{13},\rho_{23},\rho_{012},\rho_{013},\rho_{023} \right)$ a step-wise procedure is used maximizing the likelihood of the z-scores observed in the GWAS summary statistics. First, three univariate analyses are conducted to estimate univariate polygenicities ($\pi_{1}^{u},\pi_{2}^{u},\pi_{3}^{u}$), discoverabilities ($\sigma_{1},\sigma_{2},\sigma_{3}$) and residual variances ($\sigma_{01},\sigma_{02},\sigma_{03}$) for each of the three phenotypes. Then, the bivariate analyses are performed to estimate pairwise genetic overlaps ($\pi_{12}^{b},\pi_{13}^{b},\pi_{23}^{b}$), correlations of effect sizes within each of the three pairwise overlaps ($\rho_{12},\rho_{13},\rho_{23}$) and correlations between residuals ($\rho_{012},\rho_{013},\rho_{023}$) for each of the three pairs of phenotypes with univariate parameters fixed to the values obtained at the univariate step. Finally, the genetic overlap between all three phenotypes ($\pi_{123}$) is estimated with both univariate and bivariate parameters fixed to the values obtained in the univariate and bivariate steps. Phenotype pair-specific polygenicities can then be calculated as:

$\pi_{12}= \pi_{12}^{b}-\pi_{123}$,

$\pi_{13}= \pi_{13}^{b}-\pi_{123}$,

$\pi_{23}= \pi_{23}^{b}-\pi_{123}$,

and phenotype-specific polygenicities can be calculated as:

$\pi_{1}=\pi_{1}^{u}-\pi_{12}-\pi_{13}-\pi_{123}$,

$\pi_{2}=\pi_{2}^{u}-\pi_{12}-\pi_{23}-\pi_{123}$,

$\pi_{3}=\pi_{3}^{u}-\pi_{13}-\pi_{23}-\pi_{123}$.

Univariate, bivariate and trivariate log-likelihood functions are implemented using numerical integration of the characteristic function applying a trapezoidal rule with fixed step size as described previously.^6^ For further information, see Shadrin et al.^5^

Conditional Q-Q plots

Q-Q plots compare a nominal probability distribution against an empirical distribution. In the presence of all null relationships, nominal p values form a straight line on a Q-Q plot when plotted against the empirical distribution. For SMD and lifestyle behaviours SNPs and for each categorical subset (strata), -log_10_ nominal p values were plotted against -log_10_ empirical p values (conditional Q-Q plots). Leftward deflections of the observed distribution from the projected null line illustrate increased tail probabilities in the distribution of test statistics (z-scores) and consequently an over-abundance of low p values compared to that expected by chance, also called ‘enrichment’. This is illustrated in Figs. S9-10.

We can calculate quantitative estimates of likely true associations from the distributions of summary statistics, under large-scale testing paradigms, such as GWASs.^7, 8^ Conditional Q-Q plots of nominal p values from GWAS summary statistics visualizes this enrichment of statistical association relative to that expected under the global null hypothesis. The usual Q-Q curve has the nominal p value, denoted by "p", as the y-ordinate and the corresponding value of the empirical cdf, denoted by "q", as the x-ordinate. Under the global null hypothesis the theoretical distribution is uniform on the interval [0,1]. We instead plot -log_10_ p against -log_10_ q to emphasize tail probabilities of the theoretical and empirical distributions. Therefore, genetic enrichment is illustrated with a leftward shift in the Q-Q curve, corresponding to a larger fraction of SNPs with nominal -log_10_ p value greater than or equal to a given threshold. Conditional Q-Q plots are created by creating subsets of SNPs based on levels of an auxiliary measure for each SNP, and computing Q-Q plots separately for each level. If SNP enrichment is captured by variation in the auxiliary measure, this is expressed as successive leftward deflections in a conditional Q-Q plot as levels of the auxiliary measure increase. We constructed conditional Q-Q plots of empirical quantiles of nominal -log_10_ values for SNP association for all SNPs, and for subsets (strata) of SNPs determined by the nominal p values of their association with the conditional phenotypes, and vice versa. Specifically, we calculated the empirical cumulative distribution (cdf) of nominal p values for SMDs for all SNPs and for SNPs with significance levels below the indicated cut-offs for the conditional phenotypes (e.g., Healthy Food) (-log_10_(p) ≥ 1, -log_10_(p) ≥ 2, -log_10_(p) ≥ 3 corresponding to p < 0.1, p < 0.01, p < 0.001 respectively). The nominal p values (–log_10_(p)) are plotted on the y-axis, and the empirical quantiles (–log_10_(q), where q=1-cdf(p)) are plotted on the x-axis. To assess for polygenic effects below the standard GWAS significance threshold, we focused the conditional Q-Q plots on SNPs with nominal –log_10_(p) < 7.3 (corresponding to p > 5x10^-8^). We controlled for spurious enrichment by calculating all conditional Q-Q plots after random pruning averaged over 500 iterations. At each iteration, one SNP in every LD block (defined by an r^2^ > 0.1) was randomly selected and the empirical cdfs were computed using the corresponding p values.

Conditional and Conjunctional False Discovery Rate

The ‘enrichment’ seen in the conditional Q-Q plots can be interpreted in terms of true discovery rate (TDR = 1 – false discovery rate (FDR)).^9^ More specifically, for a given p value cutoff, the FDR is defined as

FDR(p) = π_0_F_0_(p) / F(p), [1]

where π_0_ is the proportion of null SNPs, F_0_ is the null cumulative distribution function (cdf), and F is the cdf of all SNPs, both null and non-null.^8^ Under the null hypothesis, F_0_ is the cdf of the uniform distribution on the unit interval [0,1], so that Eq. [1] reduces to

FDR(p) = π_0_p / F(p), [2]

The cdf F can be estimated by the empirical cdf q = N_p_ / Ν, where N_p_ is the number of SNPs with p values < p, and N is the total number of SNPs. Replacing F by q in Eq. [2], we get

Estimated FDR(p) = π_0_p / q, [3]

which is biased upwards as an estimate of the FDR^10^. Replacing π_0_ in Equation [3] with unity gives an estimated FDR that is further biased upward;

q* = p / q, [4]

If π_0_ is close to one, which is probably true for most GWASs, the increase in bias from Eq. [3] is minimal. Therefore, the quantity 1 – p/q, is biased downward and thus a conservative estimate of the TDR. Referring to the Q-Q plots, we see that q* is equivalent to the nominal p value divided by the empirical quantile, as defined previously. We can thus read the FDR estimate directly off the Q-Q plot as

-log_10_(q*) = log_10_(q) – log_10_(p), [5]

demonstrating that the estimated FDR is directly related to the horizontal shift of the curves in the Q-Q plots from the expected line x = y, i.e. a larger shift corresponds to a smaller FDR.

*Conditional FDR* (condFDR) is an extension of the standard FDR, which incorporates information from GWAS summary statistics of a conditional phenotype (e.g., Healthy Food) to adjust significance levels in the primary phenotype (e.g., SCZ). The FDR can be interpreted as the probability that a SNP is null given that its p value is as small as or smaller than its observed p value. The condFDR is defined as the probability that a SNP is null in the first phenotype given that the p values in the first and second phenotypes are as small as or smaller than the observed ones. Ranking SNPs by the standard FDR or by p values gives the same ordering of SNPs. Given the cross-trait SNP enrichment between the primary and conditional phenotypes, ranking SNPs by condFDR will reorder SNPs when the primary and conditional phenotypes are genetically related.

The *conjunctional FDR* (conjFDR) approach is used to discover shared genetic loci between two phenotypes (e.g., SCZ and Healthy Food). The conjFDR is determined after inverting the roles of the primary and conditional phenotypes and repeating the condFDR procedure. ConjFDR is defined as the posterior probability that a SNP is null for either phenotype or both simultaneously, given that its p values for association with both phenotypes are as small as or smaller than the observed p values.^11-15^ A conservative estimate of the conjFDR is obtained by the maximum of the two condFDR values for a given SNP ^16^. Furthermore, complex correlations in regions with intricate LD can bias FDR estimation.^17^ Therefore, we excluded SNPs in the extended major histocompatibility complex (MHC) and chromosome 8p23.1 (genome build 19 locations chr6: 25119106–33854733 and chr8: 7242715–12483982, respectively) and SNPs in LD (r^2^ > 0.1) with such SNPs before fitting the FDR models. Thus, we avoided artificially inflated genetic enrichment owing to the LD structure of these regions.^18^ This is common in cond/conjFDR studies of SMDs that show strong associations with these genomic regions.^18-20^ Note that this procedure involves excluding MHC and 8p23.1 before creating the conditional Q-Q plots, thereby making it possible to observe cross-trait enrichment that is not driven by the strong associations within these regions. Still, SNPs within these regions are not excluded from the cond/conjFDR analyses per se as SNPs in these regions are given a cond/conjFDR value based on their p value and may have biological relevance. MHC was also excluded when performing MiXeR analyses as the intricate LD structure creates difficulties in providing reliable model estimates.^2^ The 8p23.1 region is characterized by strongly correlated SNPs, yet less complex LD pattern, and therefore MiXeR is able to model this LD pattern^2^. P values were corrected for inflation using a genomic inflation control procedure.^11^

Genomic loci definition

We defined independent genomic loci using FUMA, an online tool for functional annotation of genetic variants (<http://fuma.ctglab.nl/>).^21^ Summary statistics from the GWASs on SMDs and lifestyle behaviours were used as input for FUMA. First, independent significant SNPs were identified as SNPs with condFDR < 0.01 and independent from each other at LD r^2^ < 0.6. Secondly, to create a subset of lead SNPs we retained the independent SNPs with LD with each other at r^2^ < 0.1. Next, distinct genomic loci were identified by merging physically overlapping lead SNPs (LD blocks < 250 kb apart). We selected a SNP with the most significant p value as a lead SNP of the merged locus. Borders of the genomic loci were determined by identifying all SNPs in LD (r^2^ ≧ 0.6) with one of the independent significant SNPs in the locus. The 1000 Genomes Project reference panel was used to calculate the LD information.^22^

Effect sizes and genetic correlations

Effect size (Z-scores) of the shared SNPs were retrieved from the GWAS summary statistics for SMDs and lifestyle behaviours.^23-27^ Genome-wide genetic correlation (*r_g_*) between each pair of phenotypes were calculated using LD score regression (LDSC).^28^ This method provides a summary measure of the correlation of effect sizes of the SNPs. LD score regression was estimated using the Python-based package available at https://github.com/bulik/ldsc, as described here: https://github.com/bulik/ldsc/wiki/Heritability-and-Genetic-Correlation. We corrected for multiple testing using Bonferroni correction: A p value of 0.05 was divided by 18 tests (3 SMDs x 6 lifestyle behaviours).

Validation in EAS ancestry samples

We also used LDSC^4^ and bivariate MiXeR^2^ to assess the genetic relationship between dietary factors and SMDs in independent EAS ancestry samples. Genetic correlations were considered statistically significant after correcting for multiple comparisons across the three SMDs and three dietary factors. The absence of data on intake of fruits in the EAS dataset precluded the generation of the composite phenotype Healthy Food^26^ similar to the one used in the primary analysis. Consequently, we were unable to assess the consistency of allelic effect directions among shared lead SNPs from conjFDR between the discovery cohorts and independent samples.

Functional annotation

We mapped lead SNPs to genes using the online source Open Targets Genetics.^29^ Open Targets is a machine learning approach that integrates information, including transcriptomics (expression quantitative trait loci, eQTL), proteomics (protein quantitative trait loci, pQTL), functional predictions based on the DNA sequence, chromatin interaction and conformation, and the distance between the variant and the transcript start site to estimate a single V2G score for each variant-gene pair. The nearest gene is usually given the highest score as the probability of being causally associated with a gene is highest for the nearest gene, although this is not always the case.^30^

For each lead SNP, we selected the gene with the highest overall score from Open Targets. The mapped genes were used as input for Gene Ontology (GO) gene-set analysis and pathway analysis using FUMA.^31^ Protein-coding genes were selected as background genes in FUMA. We did not use genes mapped to candidate SNPs in the enrichment analyses due to the high risk of false positive results, especially when the gene-set has a high number of genes. We performed these enrichment analyses genes mapped to lead SNPS shared between SMDs and each lifestyle behaviour (e.g., Healthy Food). We also conducted enrichment analyses based on genes mapped to the lead SNPs associated with the food phenotypes (i.e., Healthy Food and Meat) combined, as well as physical activity and sedentary behaviours phenotypes (i.e., PhysAct, Screen and SedWork) combined, for each SMD. This resulted in more powerful analyses, allowing the assessment of categories of lifestyle behaviours (i.e., food intake and physical activity/sedentary behaviours) in the gene-set and pathway analyses.

We performed these FUMA analyses with and without mapped genes in complex LD regions (MHC and 8p23.1), separately. Because these regions contain numerous correlated SNPs, the presence of individual associations within these clusters can drive to the apparent enrichment of entire gene-sets. Analyses were corrected for multiple testing (Bonferroni correction).

Structural Equation Modeling

We applied a structural equation modeling (SEM) approach to assess the direct and indirect association between polygenic risk score (PRS) for each SMD and CVD risk factors, including BMI and lipids, potentially mediated by lifestyle behaviours. We focused on BMI and the lipids TG and HDL as ~~a~~ relevant CVD risk factors given the previously reported distinct genetic relationships with SCZ and BIP^32-34^ compared to MD^20, 35^ and the association of BMI and lipids with lifestyle factors.^26, 27^ First, we used PRSice software version 2.3.5^36^ to calculate PRS for MD, SCZ and BIP (p value thresholds = 1e^-8^, 1e^-7^, 1e^-6^, 1e^-5^, 1e^-4^, 1e^-3^, 0.01, 0.05, 0.1, 0.5, 1) in the UKB, based on summary statistics from the GWAS datasets of SMDs after excluding UKB sample from the BIP and MD dataset. We selected the significance threshold of the best-performing PRS in an independent subsample of UKB, which was used in subsequent analyses. Next, we constructed SEM models using lavaan (version 0.6-18) in R (version 4.1)^37^ to estimate the direct and indirect associations between PRSs, BMI and lipids with the lifestyle behaviours Healthy Food, Meat, PhysAct and Screen as potential mediators. We constructed separate SEM models with each potential lifestyle mediator (i.e., each of Healthy Food, Meat, PhysAct and Screen) for each SMD PRS association with BMI and lipids. Further, building upon the genetic overlap identified in trivariate MiXeR analyses, we used SEM to further elucidate the relationship between genetic liability to SCZ and BIP, current smoking and BMI. Specifically, we assessed if current smoking mediates part of the association between SCZ and BIP PRSs and BMI. We restricted these analyses to SCZ and BIP to maintain a hypothesis-driven approach.

We used bootstrapped standard error estimates. P values were corrected for multiple testing using Bonferroni correction. All associations were adjusted for age, sex, genetic array, and the first ten genetic principal components. We estimated standardized coefficients (β) using the standardized argument in the summary function in lavaan.^37^ We created one figure per lifestyle mediator, incorporating the results for PRS for MD, SCZ and BIP. These figures were constructed to illustrate the direct associations between PRS for each SMD and BMI as the primary outcome, and indirect associations mediated by lifestyle behaviours.

SEM relies on the assumption of multivariate normality of residuals.^38^ Formal tests of normality are highly sensitive to sample size; in very large samples, even minor, negligible deviations from normality are likely to yield statistically significant results.^39^ Given the large sample analyzed in this study, we assessed the distribution of the residuals visually rather than relying on formal significance tests (e.g., Mardia’s test). Thus, we constructed figures including marginal density with univariate distribution of the residuals, and bivariate density to evaluate the joint distribution, i.e. the relationship between the residuals of the variables. We generated these figures after rank‑based inverse normal transformation (RINT)^40^ of quantitative variables and for each SEM model, including one mediator (e.g. Screen) and one outcome variable (e.g. BMI).

Mendelian Randomization

As supplementary analyses, we used Mendelian randomization (MR), which is an approach that estimates causal relationship between two variables (i.e., exposure and outcome) by leveraging the fact that individuals are randomly assigned genetic risk for the exposure of interest.^41^ MR analyses select the genetic variants associated with the exposure as “instruments”. The MR approach relies on three main assumptions: (1) the instruments are associated with the exposure, (2) the instruments are independent of confounders, (3) the instruments influence the outcome only via the exposure. Violations of these assumptions yield unreliable estimates from MR analysis and, thus, using multiple MR methods is the standard to test causal relationships.^41^ One of the more robust approaches is two-sample MR which relies on GWAS summary statistics from independent samples for the exposure (e.g., SCZ) and outcome (e.g., Healthy Food) of interest. We employed several two-sample MR methods, including inverse variance weighted (IVW) method,^42^ MR-Egger,^43^ and weighted median method,^44^ using R package (version 4.3.1, TwoSampleMR version 0.5.7).^45^ Furthermore, we used an additional approach that is more robust against violation of the assumptions and thus less prone to bias due to horizontal pleiotropy (i.e., SNPs influence the outcome through some pathway other than the exposure), MR Pleiotropy Residual Sum and Outlier (MR-PRESSO).^46^

The classic method for MR is IVW, which is a fixed-effect meta-analysis of the SNP effects on the outcome over the SNP effects on the exposure. The causal effect estimates of each genetic variant are combined in an IVW meta-analysis. Compared to the IVW approach, the weighted median and MR Egger approaches are more robust to heterogeneity in estimates, which can indicate violation of MR assumptions and horizontal pleiotropy. Further, we applied MR-PRESSO, which detects and removes outliers in estimates and then recalculates a more robust causal estimate. We focused on the relationships that were statistically significant according to at least two of the four MR methods after multiple testing correction based on FDR adjusted p-values. For further information about the MR tools, see the method publications.^42-46^

Summary statistics from the GWASs for SMDs and self-reported lifestyle factors described under the section Participant Samples were used for the MR analyses. We selected SNPs that passed genome-wide significance (p < 5 × 10^−8^), which is the standard,^41^ and clumping using PLINK with clump_p = 1, clump_r^2^ = 0.001, and clump_kb = 10000 against the 1000 Genomes Phase3 503 EUR samples keeping other settings default.

Genome-wide analyses of accelerometer-assessed activity

Below we describe the technical quality control in the All of Us, the GWAS performed in this cohort, followed by the meta-analysis of the GWAS results from All of Us and UKB.

**Quality control of whole genome sequencing data in All of Us**

We used already quality controlled population level Allele Count/Allele Frequency (ACAF) threshold callset of whole genome sequencing (WGS) data. This data had been analyzed with the Illumina DRAGEN platform optimized for mapping, alignment, sorting, duplicate marking, and haplotype variant calling,^47^ and provided to researchers through All of Us Researcher Workbench. As described by investigators in All of Us,^47^ the ACAF threshold callset includes variants that have a population-specific allele frequency > 1% or a population-specific allele count > 100 in any ancestry subpopulations. In the exome callset, variants are within the exon regions of the Gencode v42 basic transcripts, with regions expanded by 15 bases on either side of each exon. We then filtered the WGS data requiring ≥ 95% call rate and ≥ 0.5% minor allele frequency before running the GWAS.^47, 48^ In the accessed WGC data we did not apply Hardy- Weinberg equilibrium filter. Further, relatives were excluded from the dataset, in line with the procedure described by the research team in All of Us.^48^ The accessed data includes an independent set of 30,132 individuals following kinship filtering.^48^ For further details on quality control and laboratory methods for the genomic data in All of Us, see the comprehensive overview provided by the research program’s investigators.^48^

**Association analyses in All of Us**

We used RINT^40^ to AccPhysAct and AccSed, ensuring normally distributed data as input to the GWAS. A GWAS of WGS variant call data was performed using PLINK2.^49^ The covariates included sex at birth, device type, device version, season of wear, age, age squared, and the first 16 genetic principal components to account for population stratification, which were precalculated. The calculation of these principal components is described by the research team in All of Us.^48^ We followed the FUMA protocol^21^ to define a genome-wide significant locus (p < 5 x 10^-8^) in the GWAS and meta-analysis, using the same boundaries as described for cond/conjFDR locus. The summary statistics underwent preprocessing and cleaning based on the cleansumstats pipeline (https://github.com/BioPsyk/cleansumstats),^50^ yielding 9,9249,43 SNPs for subsequent analyses.

**Meta-analysis**

The meta-analysis of summary statistics for AccPhysAct and AccSed in All of Us and UKB was performed using inverse-variance-weighted fixed effects models in METAL.^51^ Prior to analyses, the data in UKB was also processed using the cleansumstats pipeline,^50^ which resulted in 11,777,262 SNPs for meta-analysis.

Genetic overlap between accelerometer-assessed activity and SMDs

The combined summary statistics were used to assess genetic overlap with SMDs using bivariate MiXeR^2^ and LDSC.^4^ Genetic correlations were deemed significant after correction for multiple comparisons (i.e., a p value of 0.05 was divided by 6 tests (3 SMDs x 2 accelerometer-derived phenotypes).

Supplementary Results

MiXeR estimates

Univariate MiXeR estimated different SNP-based heritability of SMDs and the lifestyle behaviours (Table S1). SCZ (h^2^_SNP_ = 0.38) possessed highest h^2^_SNP_, followed by BIP (h^2^_SNP_ = 0.19). MD (h^2^_SNP_ = 0.07) demonstrated similar SNP-based heritability as some of the lifestyle behaviours, including Healthy Food (h^2^_SNP_ = 0.06), Meat (h^2^_SNP_ = 0.06), and Screen (h^2^_SNP_ = 0.08), while SedWork (h^2^_SNP_ = 0.03) and SedCom (h^2^_SNP_ = 0.02) possessed lower SNP-based heritability (Table S1).

Univariate Q-Q plots indicate that MiXeR-based predictions provide accurate estimates of the data plots, in which the orange line (model predictions) follow the blue line (observed GWAS data) with a leftward shift from the dashed line (null line) (Fig. S1). The exception was SedCom as the univariate Q-Q plot shows that the blue line (observed GWAS data) closely follows the dashed line, indicating that the GWAS for SedCom is under-powered (Fig. S1), in line with the negative AIC value (Table S1).

Bivariate analyses revealed extensive genetic overlap (Fig. 1; Figs. S2-4). MiXeR suggested that the majority of the variants influencing MD also influence Healthy Food (9.5K), PhysAct (9.5K), Screen (10.9K) (Fig. 1A-C) and Meat (8.1K) (Fig. S2). Most of the SCZ-influencing variants were also shared with Healthy Food (7.5K), PhysAct (9.0K), Screen (9.4K) (Fig. 1D-F) and Meat (7.7K) (Fig. S3). Furthermore, the majority of the BIP-influencing variants were estimated to influence Healthy Food (7.5K), PhysAct (8.0K) and Screen (8.7K) (Fig. 1G-I), and considerable, yet less, overlap was found with Meat (5.9K) (Fig. S4), which is less polygenic. Finally, SMDs shared fewer SNPs with SedWork, the least polygenic trait, although nearly all the SedWork-influencing variants were shared with SMDs. Similarly, the majority of SNPs influencing the other lifestyle behaviours (Healthy Food, Meat, PhysAct and Screen) were shared with SMDs (Figs. S2-4). The Dice coefficient (i.e., the percentage of shared variants between SMDs and each lifestyle behaviours out of the total number of SNPs influencing both traits) is provided in Table 2, showing that most of the Dice coefficients were between 70% and 90%.

The bivariate analyses demonstrated acceptable model fit (Table S2 and Figs. S2-4) showing positive best vs. minimum overlap AIC differences for all 15 analyzed pairs of phenotypes (i.e. the MiXeR-modeled overlap fitted genetic signal observed for two phenotypes better than the model with minimum genetic overlap for a given level of genetic correlation). For most of phenotype pairs, estimated genetic overlap was almost complete and MiXeR model was not able to distinguish modeled overlap from maximum possible overlap in 10 out of 15 analyses. The exceptions included SCZ and Healthy Food and Meat, BIP and Healthy Food, Meat and PhysAct, where the model indicated existence of phenotype-specific variants for both SMDs and lifestyle behaviours. For the phenotype pairs where MiXeR indicated less overlap (SMDs and SedWork, and BIP and Meat), with higher uncertainty of the model estimates (reflected in relatively broad standard deviations), larger GWAS samples are likely to improve accuracy of bivariate estimates.

Further, parameter optimization for number of shared variants is illustrated in the log-likelihood curves (Fig. S2-4), consistent with the AIC values. The lowest point on the curve indicates the number of shared variants after parameter optimization. A positive AIC difference compared to minimum overlap was illustrated by a descending curve from the minimum overlap to the lowest point on the curve for all phenotype pairs. A positive AIC difference compared to maximum overlap was visualized as an ascending curve to the maximum number of shared variants. The latter was observed for phenotype pairs where MiXeR did not estimate close to complete genetic overlap, as mentioned above. Moreover, the conditional Q–Q plots indicated that the MiXeR-modeled predictions follow the data Q–Q plots, although less closely at lower p values for some phenotype pairs (Figs. S2-4).

Genetic correlations: Despite considerable genetic overlap between SMDs and lifestyle behaviours, modest genome-wide genetic correlations (*r_g_* = -0.22–0.17) were estimated using LDSC (Table 2). We considered the genetic correlations after Bonferroni correction. 18 tests (3 SMDs x 6 lifestyle behaviours) resulted in adjusted significance level of 0.05/18 = 0.0028. MD had a significant negative *r_g_* with PhysAct, and a positive *r_g_* with Screen (Table 2). SCZ and BIP demonstrated a positive *r_g_* with Healthy Food, while a negative *r_g_* with Screen. Additionally, BIP demonstrated a positive *r_g_* with PhysAct. There was a negative *r_g_* between all three SMDs and Meat, although this was not significant for BIP after Bonferroni correction. The *r_g_* between SMDs and SedWork and SedCom was also non-significant after Bonferroni correction.

We performed trivariate MiXeR to estimate the amount of overlap between each of SCZ; BIP and MD and two lifestyle behaviours (e.g., Healthy Food and PhysAct) (Tables S3-5; Fig. S6). Trivariate MiXeR estimated the greatest amount of genetic overlap between MD, Healthy Food and Meat (53%), here and below the percentage indicating genetic overlap among triad of phenotypes is given with respect to the total amount of variants influencing all three phenotypes), SCZ, Meat and Healthy Food (49%), and SCZ, Screen and PhysAct (43%). There was least trivariate genetic overlap between the following phenotype trios: PhysAct, Meat and each of the SMDs (14% for MD, 12% for SCZ, and 16% for BIP). The average amount of trivariate overlap was 27% and the median was 25%, indicating that approximately one-quarter of the SNPs are shared across three phenotypes (Tables S3-5). The trivariate overlap was 44% for SCZ, BMI, and smoking, and 37% for BIP, BMI, and smoking (Figs. S7-8; Tables S6-7). The amount of discrepancy between trivariate model (Figs. S6-8A) and the results deduced based on bivariate analyses (Figs. S6-8B) was small, 0-5% (Tables S3-7). The largest difference between the overlap pattern estimated with trivariate MiXeR and overlap pattern expected from three bivariate analyses under the assumption of maximum entropy was found for BIP, Healthy Food and SedWork of ca. 5% (Table S5; Fig. S6).These estimates indicate that the trivariate model-based predictions were close to similar to the patterns of genetic overlap deduced from bivariate analyses under the assumption of maximum entropy.^5^

Cross-trait enrichment

In the conditional Q-Q plots, we observed enrichment in SMDs SNPs as a function of the significance of associations with lifestyle behaviours (Fig. S9). This enrichment is visualized as successive leftward deflections from the null distribution, which can be interpreted in terms of the true discovery rate (1-FDR).^11^ The reverse conditional Q-Q plots also illustrated enrichment in lifestyle behaviours given associations with SMDs (Fig. S10). This indicates polygenic overlap between SMDs and lifestyle behaviours. However, there was no evidence of enrichment of SMDs SNPs conditional on their associations with SedCom, and vice versa, as we did not observe successive increments of SNP enrichment for SMDs as a function of the significance of association with SedCom, and vice versa (Figs. S9-10). Therefore, SedCom was not included in the subsequent analyses.

Conditional False Discovery Rate results

To increase statistical power, we leveraged the pleiotropic enrichment using condFDR analysis and re-ranked SMD SNPs conditional on their association with lifestyle behaviours, and vice versa. At condFDR < 0.01, we identified 48 loci associated with MD conditional on Healthy Food, 57 loci conditional on Meat, 52 loci conditional on PhysAct, 54 loci conditional on Screen, and 51 loci conditional on SedWork (Tables S9-13). Additionally, we discovered 280 loci associated with SCZ conditional on Healthy Food, 253 loci conditional on Meat, 287 loci conditional on PhysAct, 370 loci conditional on Screen, and 267 loci conditional on SedWork (Tables S14-18). We discovered 101 loci associated with BIP conditional on Healthy Food, 78 loci conditional on Meat, 127 loci conditional on PhysAct, 155 loci conditional on Screen, and 94 loci conditional on SedWork (Tables S19-23). We also identified loci associated with lifestyle behaviours conditional on their associations with SMDs (see second panel in Tables S9-23).

Conjunctional False Discovery Rate results

An overview of the conjFDR results is presented in Table 3. At conjFDR < 0.05, MD shared 35 loci with Healthy Food, 61 loci with Meat, 23 loci with PhysAct, 46 loci with Screen, and 8 loci with SedWork (Fig. 2A-C; Fig. S11A-B; Tables S24-28). Furthermore, SCZ shared 108 loci with Healthy Food, 67 loci with Meat, 54 loci with PhysAct, 258 loci with Screen, and 19 loci with SedWork (Fig. 2D-F; Fig. S11C-D; Tables S29-33). Finally, BIP had 39 loci in common with Healthy Food, 26 loci with Meat, 40 loci with PhysAct, 188 with Screen, and 9 loci with SedWork (Fig. 2G-I; Fig. S11E-F; Tables S34-38). After merging physically overlapping loci, we identified a total of 551 distinct loci shared between SMDs and lifestyle behaviours at conjFDR < 0.05 (Table S39).

We determined the directionality of effects of lead SNPs within the shared loci (Table 3; Tables S24-38): 57.1% were concordant for MD and Healthy Food, 37.7% were concordant for MD and Meat, 21.7% were concordant for MD and PhysAct, 69.6% were concordant for MD and Screen, and 62.5% were concordant for MD and SedWork. Furthermore, 66.7% of the lead SNPs had concordant effect direction on SCZ and Healthy Food, 26.9% on SCZ and Meat, 44.4% on SCZ and PhysAct, 40.7% on SCZ and Screen, and 57.9% on SCZ and SedWork. Finally, 87.2% had concordant effect directions on BIP and Healthy Food, 57.7% on BIP and Meat, 70.0% on BIP and PhysAct, 37.2% on BIP and Screen, and 66.7% on BIP and SedWork.

MiXeR indicated a more mixed pattern of effect directions (Table 2) compared to conjFDR (Table 3). However, the predominance of concordant or discordant allelic effect directions among the shared loci at conjFDR < 0.05 was modest, consistent with the level of the genetic correlation (Table 2). The difference between the effect directions estimated by MiXeR and conjFDR likely stems from the smaller number of shared genetic loci at conjFDR < 0.05, whereas MiXeR estimates the total number of shared variants based on mathematical modelling.^2^

Functional annotation

We mapped the shared lead SNPs to genes using Open Targets (Tables S24-38). We performed gene-set analyses of genes mapped to lead SNPs (Tables S40-48). The genes shared between SCZ and Healthy Food did not implicate any gene-set after removing MHC and 8p23.1 (Table S40). After excluding these genomic regions, analyses of genes shared between SCZ and Meat revealed several synaptic and neuronal GO terms, including “postsynaptic density membrane” and “neuron to neuron synapse”, as well as one overrepresented pathway, ”Rett syndrome causing genes” (Table S41). The GO terms enriched among the genes shared between SCZ and Screen included “head development” and “behavior”, and neurodevelopmental gene-sets (Table S42). The GO term, “cell cell adhesion”, was significantly associated with genes shared between SCZ and PhysAct (Table S43). The enriched gene-sets associated with BIP and Screen included neuronal and synaptic gene-sets, such as “neuron development” and “synapse organization” (Tables S44). There were no significant gene-set results for MD and individual lifestyle behaviours.

We performed enrichment analyses based on genes mapped to the lead SNPs associated with the food intake phenotypes combined as well as physical activity and sedentary behaviours combined, for each SMD. There were several enriched neurodevelopmental gene-sets, including, “generation of neurons” and “dendrite development”, associated with the genes shared between food intake and MD and SCZ (Tables S45-46), but none for BIP and food intake. The genes shared between SCZ and physical activity/sedentary behaviours also showed enrichment in several GO terms, including “head development” and “central nervous system development” (Table S47). For genes shared between BIP and physical activity/sedentary behaviours, there were enriched gene-sets associated with neurodevelopment, including “neurogenesis” (Tables S48). Gene-sets associated with cellular processes were also enriched with the shared genes between physical activity/sedentary behaviours and SCZ and BIP, while none for MD. The enrichment analyses including genes in MHC and 8p23.1 (see the second set of results in Tables S40-48) provided findings close to those identified after excluding these genomic regions.

Mediation analyses with SEM

The figures for assessing the assumption of multivariate normality are provided in Figs. S12-14.

Mediation analyses of direct and indirect associations between PRS for SMDs and BMI via lifestyle behaviours

The threshold of the best-performing PRS for MD, SCZ and BIP was a p value equal to 1, 0.05 and 1.00e^-7^, respectively. The separate SEM models for each lifestyle mediator are illustrated in Figs. S15-18. SEM suggested significant direct associations between PRS for SMDs and BMI after correction for multiple testing. The direct associations vary slightly cross models because each model adjusts for a different mediator, which subtly alters the portion of the variance accounted for by the direct path versus the specific indirect path being tested. Thus, we report the range for the beta estimate from each model here and do the same for SEM with lipids below. PRS for MD was positively associated with BMI (β = 0.020 to 0.024, p < 0.001), while PRS for SCZ and BIP was negatively associated with BMI (SCZ: β = -0.024 to -0.022, p < 0.001; BIP: β = -0.012 to -0.009, p = 1.17e^-8^ to 1.06e^-5^) (Figs. S15-18).

Further, the SEM analyses suggested significant associations between PRS for SMDs and lifestyle behaviours, in line with the direction of associations found in the analyses of GWAS samples (Figs. S15-18). PRS for MD was negatively associated with PhysAct (β = -0.025; 95% CI, -0.030, -0.019; p < 0.001) and Meat (β = -0.005; 95% CI, -0.009, -0.002; p = 0.009) and positively associated with Screen (β = 0.017; 95% CI, 0.013, 0.021; p < 0.001). PRS for SCZ and BIP was positively associated with Healthy Food (SCZ PRS: β = 0.029; 95% CI, 0.025~~4~~, 0.033~~4~~; p < 0.001; BIP PRS: β = 0.011~~4~~; 95% CI, 0.007, 0.015; p = 8.18e^-8^), and negatively associated with Meat (SCZ PRS: β = -0.006; 95% CI; -0.010; -0.002; p = 0.005; BIP PRS: β = -0.004; 95% CI. -0.008, -0.001; p=0.04) and Screen (SCZ PRS: β= -0.012; 95% CI, -0.017, -0.008; p = 2.63e^-3^; BIP PRS: β = -0.015; 95% CI -0.019, -0.011; p = 1.92e^-13^). BIP PRS was also positively associated with PhysAct (β = 0.008; 95% CI, 0.003, 0.013; p = 0.003).

The SEM mediation models indicated indirect associations between PRS for SMDs and BMI mediated by lifestyle behaviours (Figs. S15-18). The relationship between PRS for MD and BMI was partly mediated through PhysAct (β = 0.003; 95% CI, 0.003, 0.004; p < 0.001), Screen (β = 0.004; 95% CI; 0.003, 0.005; p < 0.001), and Meat (β = -0.001; 95% CI; -1.23e^-3^, -2.05e^-4^ p = 0.009). The association between PRS for SCZ and BMI was in part mediated by Healthy Food (β = -4.08e^-4^; 95% CI,-5.53e^-4^; -2.77e^-4^; p = 6.01e^-9^), and Meat (β = -0.001; 95% CI; -1.28e^-3^, -2.53e^-4^; p = 0.005), and Screen (β = -0.003; 95% CI, -3.48e^-3^, -1.78e^-3^; p = 2.47e^-9^). The association between PRS for BIP and BMI was in part mediated by Healthy Food (β = -1.61e^-4^; 95% CI, -2.42e^-4^, -9.14e^-5^; p = 2.32e^-5^), and Meat (β = -5.49e^-4^; 95% CI, -0.001, -7.79e^-5^; p = 0.004), PhysAct (β = -0.001; 95% CI, -1.75e^-3^; -3.88e^-4^; p = 0.003) and Screen (β = -0.003; 95% CI, -0.004; -0.002~~-~~; p = 2.32e^-13^).

Mediation analyses of direct and indirection associations between PRS for SMDs and lipids via lifestyle behaviours

The SEM analyses indicated a positive direct association of MD PRS with TG (β = 0.014 to 0.017, p < 0.001) and a negative direct association with HDL (β = -0.012 to -0.010, p < 0.001). SEM suggested that part of the association between PRS for MD and TG is mediated by PhysAct (β = 0.003; 95% CI, 0.002, 0.004; p < 0.001), Screen (β = 0.002; 95% CI, 0.002, 0.003; p = 3.88e^-16^), and Meat as a mediator (β = -3.73e^-4^; 95% CI, -6.392e^-4^, -8.49e^-5^; p = 0.01). There was an indirect association between MD PRS and HDL with PhysAct (β = -0.003; 95% CI, -3.48e^-3^, -2.29e^-3^; p < 0.001), Screen (β = -0.002; 95% CI, -2.42e^-3^, -1.55e^-3^; p < 0.001), and Meat (β = 1.64e^-4^; 95% CI, 4.76e^-5^, 2.87e^-4^; p = 0.01) as mediators. Since the indirect association is the product of two pathways (e.g., MD PRS ~ Meat and Meat ~ TG), with one of them being negative (MD PRS ~ Meat), the indirect association with TG became negative, yet smaller. The indirect association with HDL via Meat became positive as each of the paths in the equation were negative (MD PRS ~ Meat and MD PRS ~ HDL).

The analyses indicated a weak positive direct association of SCZ PRS with HDL (β = 0.015 to 0.016, p < 0.001). The association with HDL was partially mediated through Healthy Food (β = 0.001; 95% CI, 1.17e^-3^, 1.60e^-3^; p < 0.001), Meat (β = 1.72e^-4^ ;95% CI, 5.05e^-5^, 2.93e^-4^; p = 7.94e^-3^), and Screen (β = 0.001; 95% CI, 9.50e^-4^, 1.93e^-3^; p = 5.06e^-9^). SEM demonstrated a negative, yet non-significant direct association between SCZ PRS and TG (β = -0.001 to 5.49e^-4^; p > 0.05), but significant indirect associations via Healthy Food (β = -0.002; 95% CI, -2.35e^-3^, -1.75e^-3^; p < 0.001), Meat (β = -3.92e^-4^; 95% CI, -6.70e^-4^, -1.04e^-4^; p = 7.63e^-3^), and Screen (β = -0.002; 95% CI, -2.04e^-3^, -1.06e^-3^; p = 4.52e^-9^).

While there was no significant direct association between BIP PRS and lipids (HDL: β = 0.002 to 0.003, p > 0.05; TG: β = 0.001 to 0.002; p > 0.05), SEM indicated an indirect association between BIP PRS and lower TG through Healthy Food (β = -7.71e^-4^; 95% CI, -1.04e^-3^, -4.80e^-4^; p = 7.55e^-7^), Meat (β = -2.88e^-4^ ; 95% CI, -5.70e^-4^, -2.42e^-5^; p = 0.048), PhysAct (β = -9.51e^-4^; 95% CI, -1.54e^-3^, -3.67e^-4^; p = 0.004), and Screen β = -0.002; 95% CI, -0.002, -0.001; p = 1.38e^-13^). In addition, there was an indirect association with increased HDL via Healthy Food (β = 5.29e^-4^; 95% CI, 3.46e^-4^ , 7.26e^-4^; p = 1.31e^-7^), Meat (β = 1.26^-4^; 95% CI, 6.10e^-4^ , 2.50e^-4^; p = 0.049 ), PhysAct (β = 9.72e^-4^; 95% CI, 3.69e^-4^ , 1.60e^-3^; p = 6.09e^-3^), and Screen (β = 0.002; 95% CI, 0.001, 0.002; p = 2.94e^-12^).

Mediation analyses of direct and indirection associations between PRS for SCZ and BIP and BMI via smoking

SEM indicated a negative association between smoking and BMI (β = -0.105; 95% CI, -0.119, -0.091, p < 0.001). There was an indirect association between SCZ PRS and BMI via smoking (β = -6.24e^-4^; 95% CI, -7.92e^-4^, -4.81e^-4^; p = 1.21e^-15^), while no significant indirect association was found for BIP PRS (β = -8.32e^-5^; 95% CI, -2.10e^-4^, 3.32^-5^; p = 0.21).

MR

MR analyses yielded statistically significant findings for several phenotype pairs but without consensus across methods. We observed a causal effect from Screen to higher MD risk according to three MR-methods, including MR-PRESSO, inverse weighted variance and weighted median (Tables S49-50). Additionally, MR-PRESSO and inverse variance weighted indicated a causal effect from SCZ to less Meat and more Healthy Food, and from BIP to decreased Screen. There was also a significant positive causal effect of Healthy Food on BIP risk according to MR-PRESSO and weighted median (Tables S49-50).

Loci associated with accelerometer-assessed activity

We found one genome-wide significant locus (p < 5 × 10^-8^) associated with AccSed, while there was no significant locus for AccPhysAct in the GWAS of All of Us (Table S51). In the meta-analyses of data from UKB and All of Us, three and seven loci reached genome-wide significance for AccPhysAct and AccSed, respectively (Table S52). One of the loci for AccPhysAct and five of the loci for AccSed were not identified in the UKB GWAS of accelerometer data.^52^ Due to differences in the locus definition and the software used for association testing in the UKB GWAS, some of the previously reported loci were not replicated here.

Genetic overlap between accelerometer-assessed activity and SMDs

The genetic correlations between accelerometer measures and self-reported phenotypes are provided in Table S53, showing significant positive correlations between AccPhysAct and PhysAct (*r_g_* = 0.32), and between AccSed and Screen and SedWork (*r_g_* = 0.21-0.39).

Univariate MiXeR estimates are provided in Table S54. Adequate model fit was indicated by the positive AICs and the univariate Q-Q plots for observed versus predicted GWAS p values in Fig. S19.

Bivariate MiXeR estimated substantial polygenic overlap between SMDs and AccPhysAct and AccSed, with Dice coefficients ranging from 74% for BIP and AccPhysAct to 90% for MD and AccPhysAct (Figs. S20-22). The analyses demonstrated acceptable model fit (Table S55; Figs. S20-22) for the bivariate estimates (Table S56). The AIC values indicated that MiXeR-modeled overlap was distinguishable from minimum overlap (positive AIC_best_vs_min_). The MiXeR estimates were indistinguishable from maximum overlap, as indicated by negative AIC _best_vs_max_, except for the pair BIP and AccSed (Table S55). Consistent with these AIC values, the log-likelihood curves for each phenotype pair illustrated a descending curve from minimum overlap to the curve’s lower point (Figs. S20-22). For BIP and AccSed, there was also an ascending curve to maximum overlap (Fig. S22), while this was not observed for the other phenotypes pairs with almost complete genetic overlap (Figs. S20-22). These findings indicate that there is extensive genetic overlap, yet more powerful GWAS data are likely to improve the estimates given the proximity to complete overlap.

The genetic correlations from LDSC are provided in Table S56, including significant negative genetic correlations between MD and AccPhysAct (*r_g_* = -0.12), and SCZ & AccSed (*r_g_* = -0.09). The negative genetic correlation between BIP and AccSed (*r_g_* = -0.07), and SCZ and AccPhysAct (*r_g_* = -0.07), did not reach statistical significance after multiple testing correction. The MiXeR-estimated proportion of shared variants with concordant effects were in line with the modest genetic correlations (Table S56).

Supplementary Figures

MD

SCZ

BIP

Healthy Food

Meat

PhysAct

Screen

SedCom

SedWork

Univariate Q-Q plots for distribution of expected p values under a null model (no SNPs associated with the phenotype) (x axis) versus observed p values (y axis). Univariate Q-Q plots demonstrate that MiXeR-based predictions provide accurate estimates of the data Q-Q plots except for SedCom. Blue lines indicate p values of SNPs observed in GWAS summary statistics with grey shading indicating 95% confidence interval. Orange lines indicate model predictions. The dashed line is the expected Q-Q plot under null (no SNPs associated with the phenotype). The vertical axes are limited to the genome-wide significance threshold of p < 5×10^−8^, to highlight behaviour of polygenic component. Points on the Q-Q plot are weighted according to LD structure, using n = 64 iterations of random pruning at LD threshold r^2^ = 0.1. MD, major depression; SCZ, schizophrenia; BIP, bipolar disorder; Healthy Food, healthy food intake; Meat, meat consumption; PhysAct, Moderate-to-vigorous intensity physical activity; Screen, leisure screen time; SedWork, sedentary behaviour at work; SedCom, sedentary commuting.


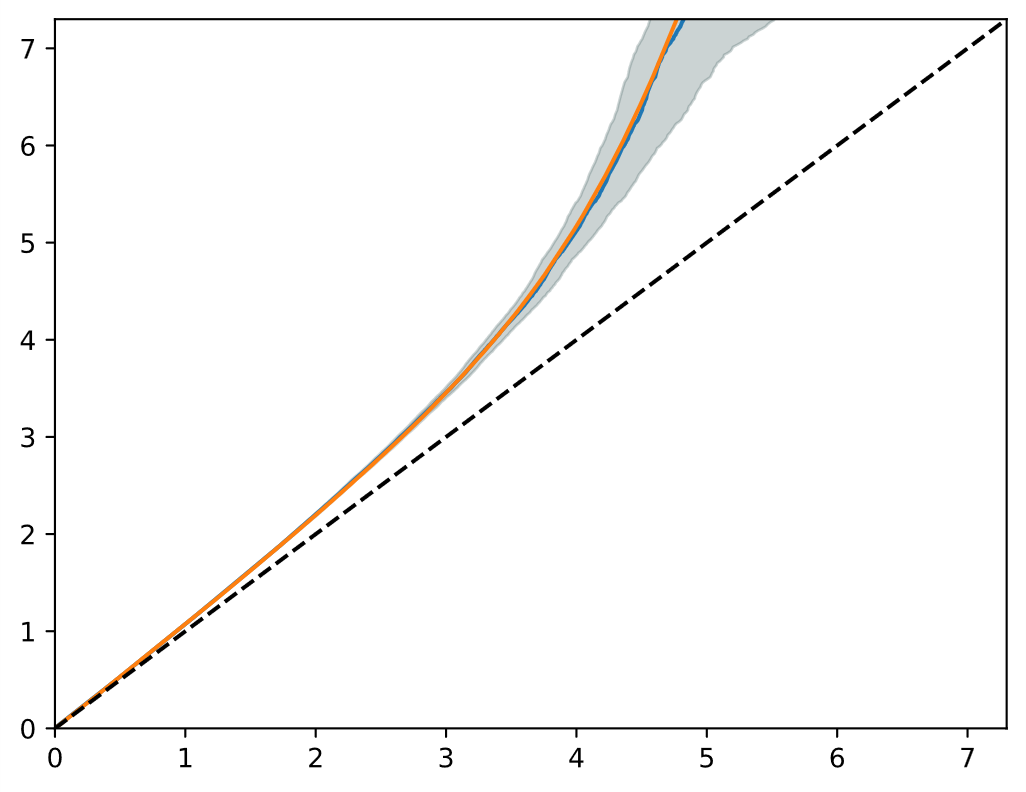

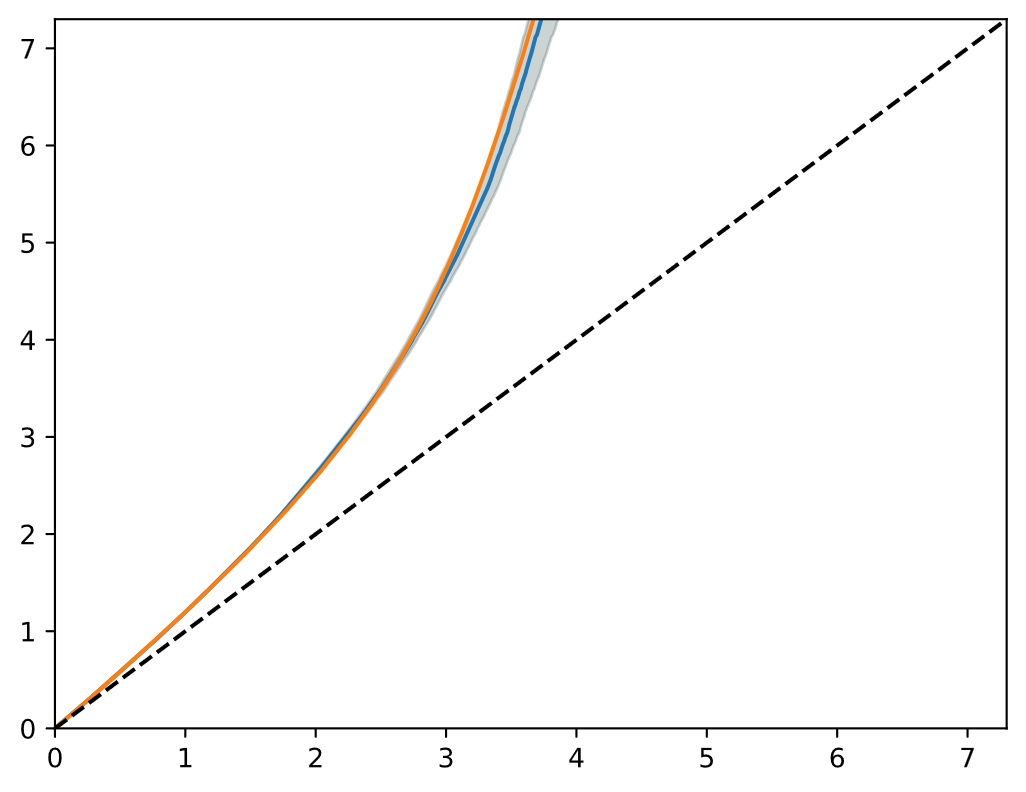

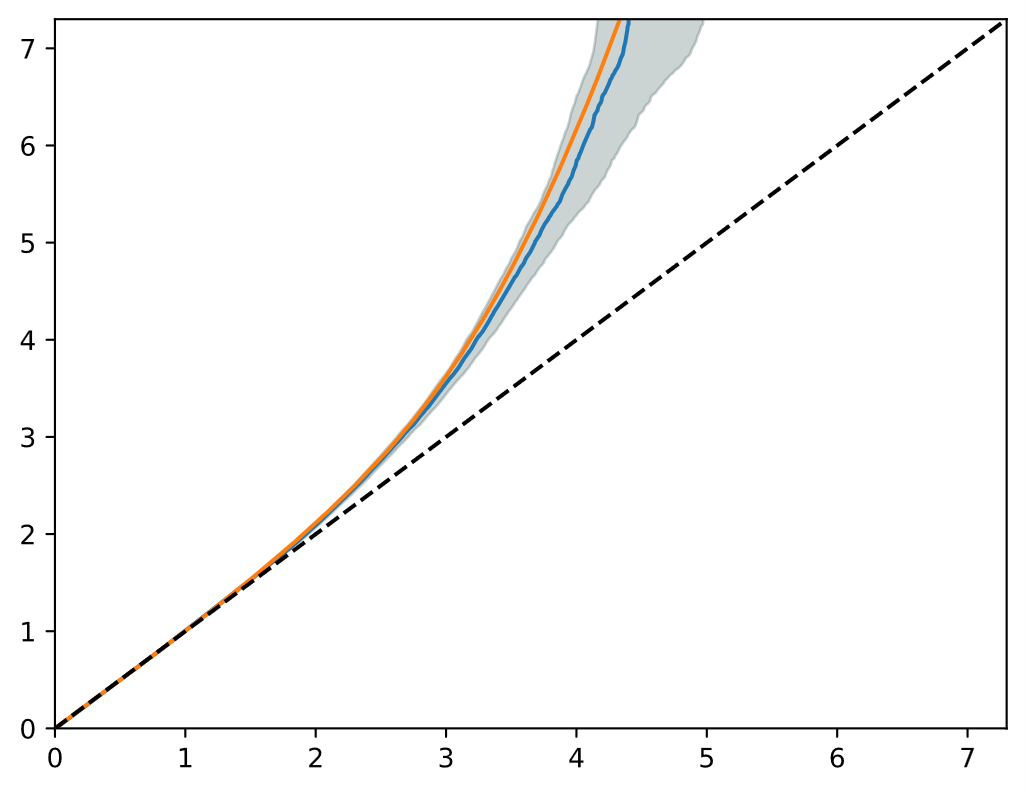

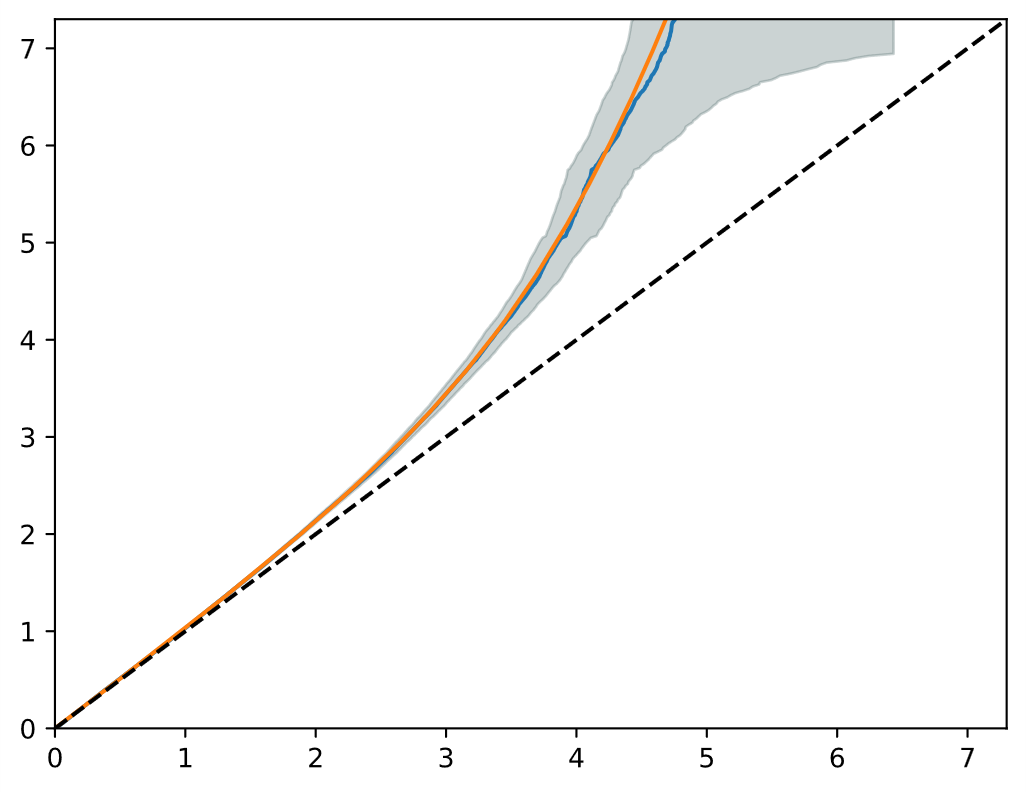

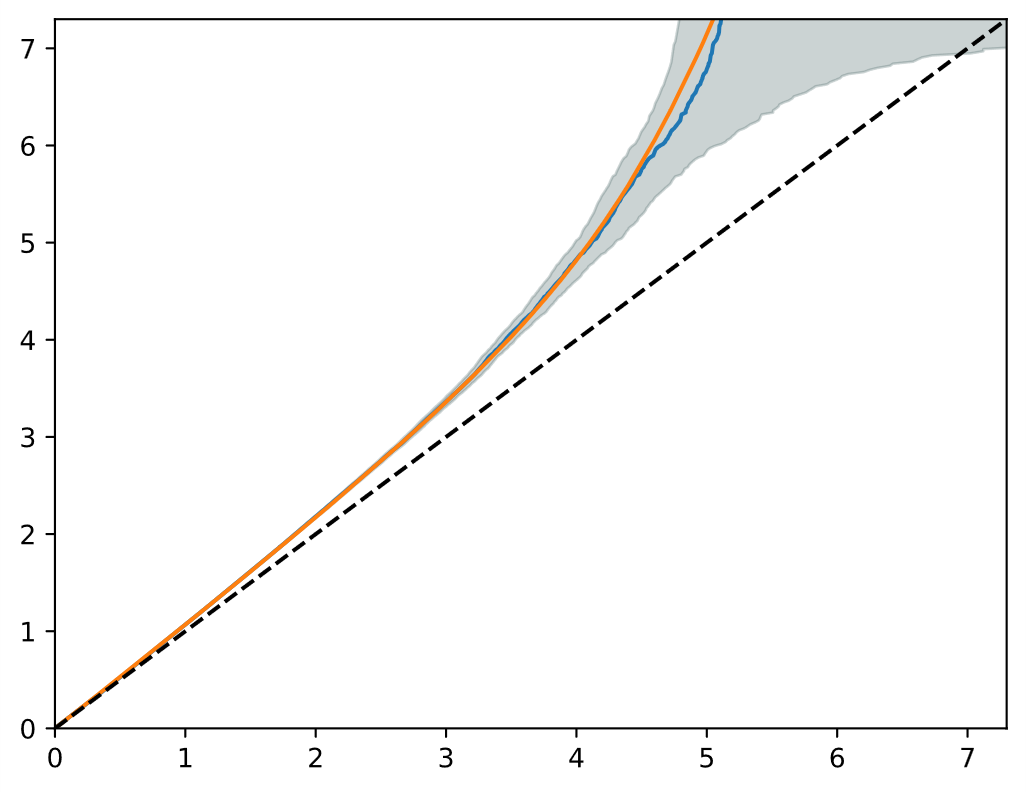

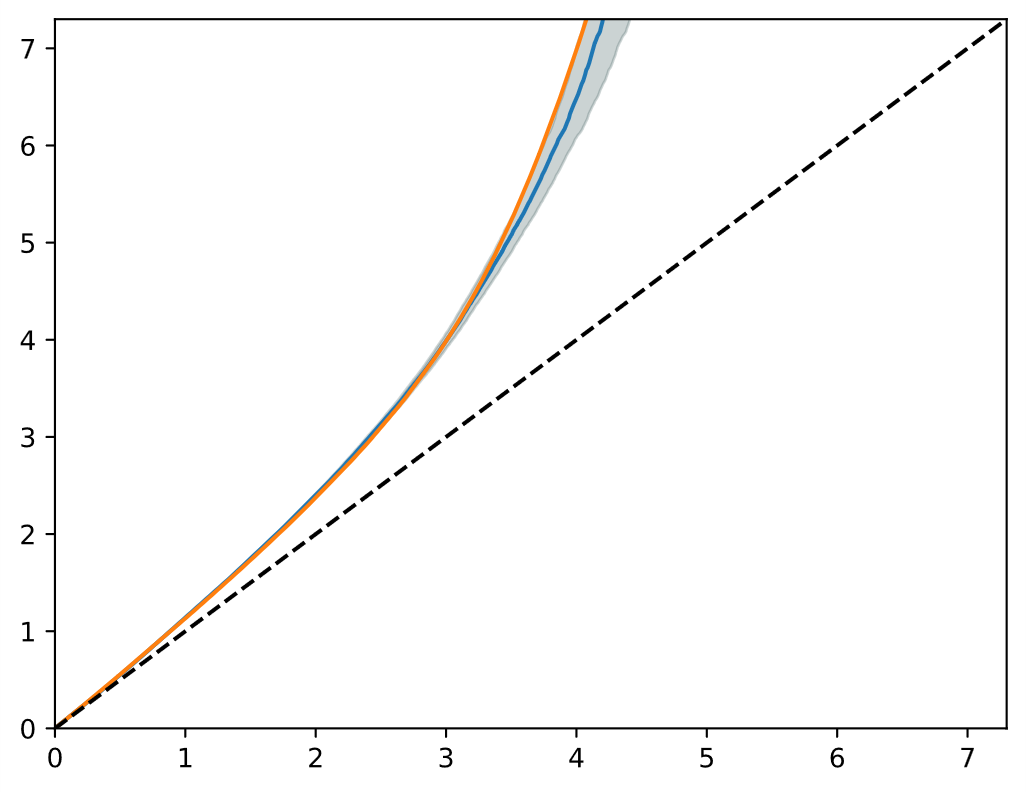

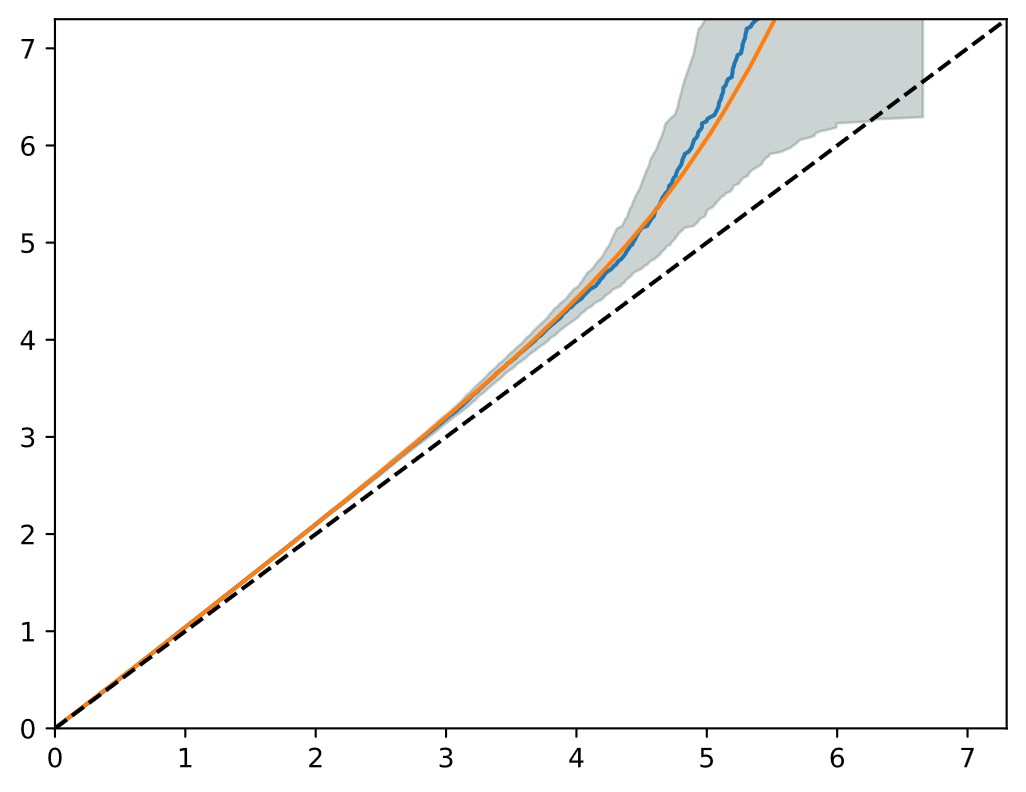

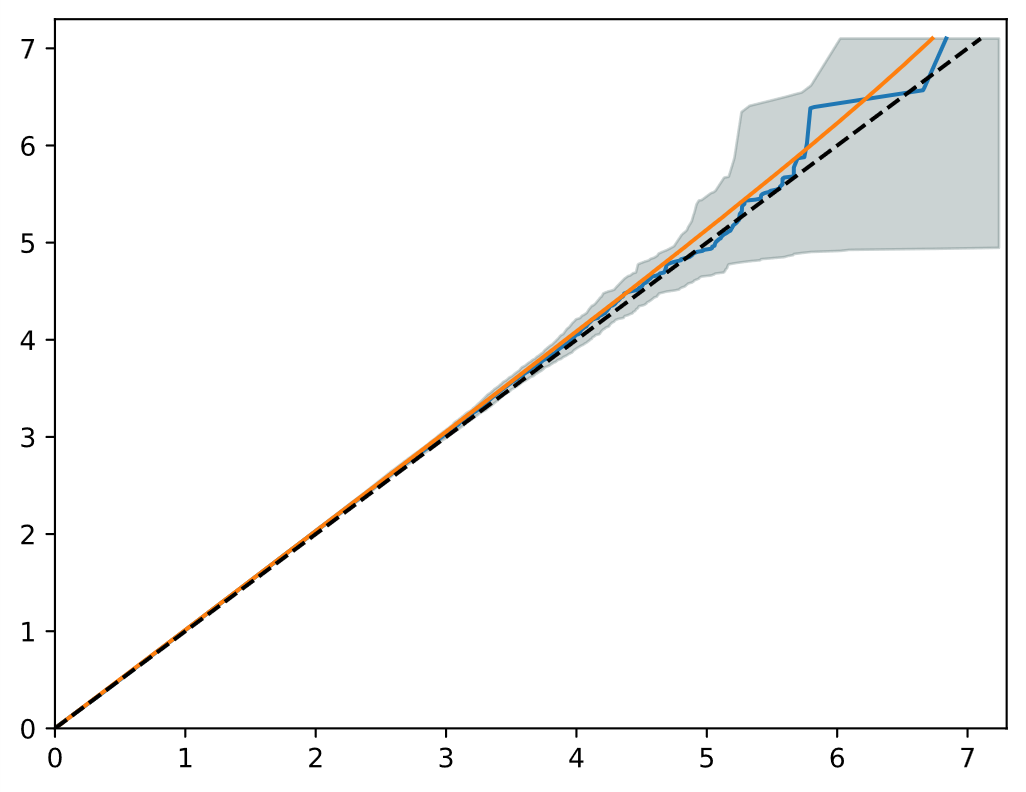

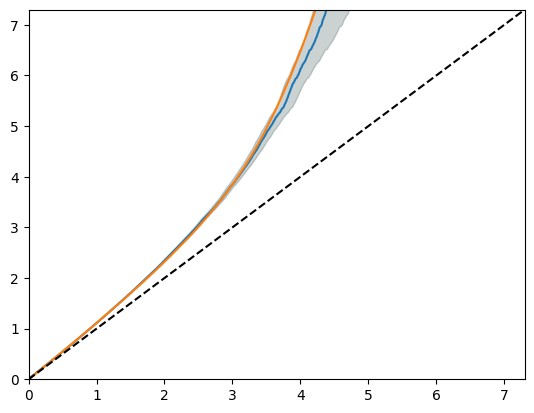


**Fig. S1. Univariate Q-Q plots from MiXeR for SMDs and lifestyle factors**

Venn Diagrams, conditional Q-Q plots, and negative log-likelihood plots, respectively. Venn diagrams of shared and unique trait-influencing variants, showing polygenic overlap (gray) between major depression (MD), (blue) and lifestyle factors (orange). The numbers in the Venn diagram indicate the estimated quantity of trait-influencing variants (in thousands), explaining 90% of SNP heritability in each phenotype, followed by standard error. Conditional Q–Q plots of observed versus expected −log_10_ p values in the primary trait as a function of significance of association with a secondary trait at the level of p < 0.1, p < 0.01, p < 0.001. Blue line indicates all SNPs. Dotted lines in blue, orange, green, and red indicate model predictions for each stratum. Black dotted line is the expected Q–Q plot under null hypothesis. Negative log-likelihood plot: minus log-likelihood calculated for the bivariate model as a function of 𝜋 parameter. The remaining parameters of the model were constrained to their fitted values. MD, major depression; Healthy Food, healthy food intake; Meat, meat consumption; PhysAct, Moderate-to-vigorous intensity physical activity; Screen, leisure screen time; SedWork, sedentary behaviour at work.


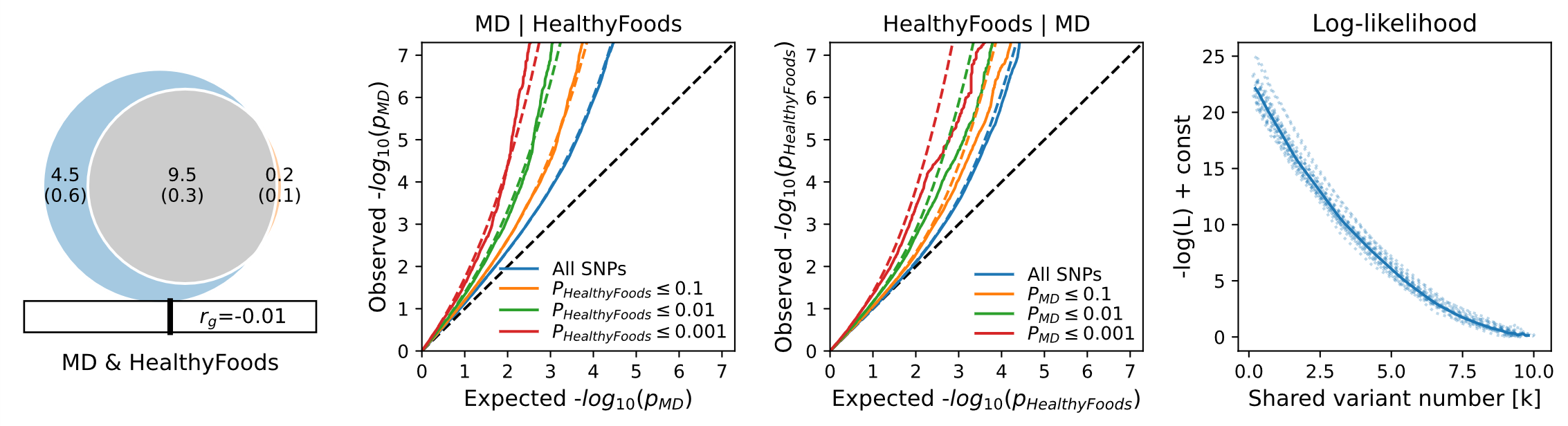

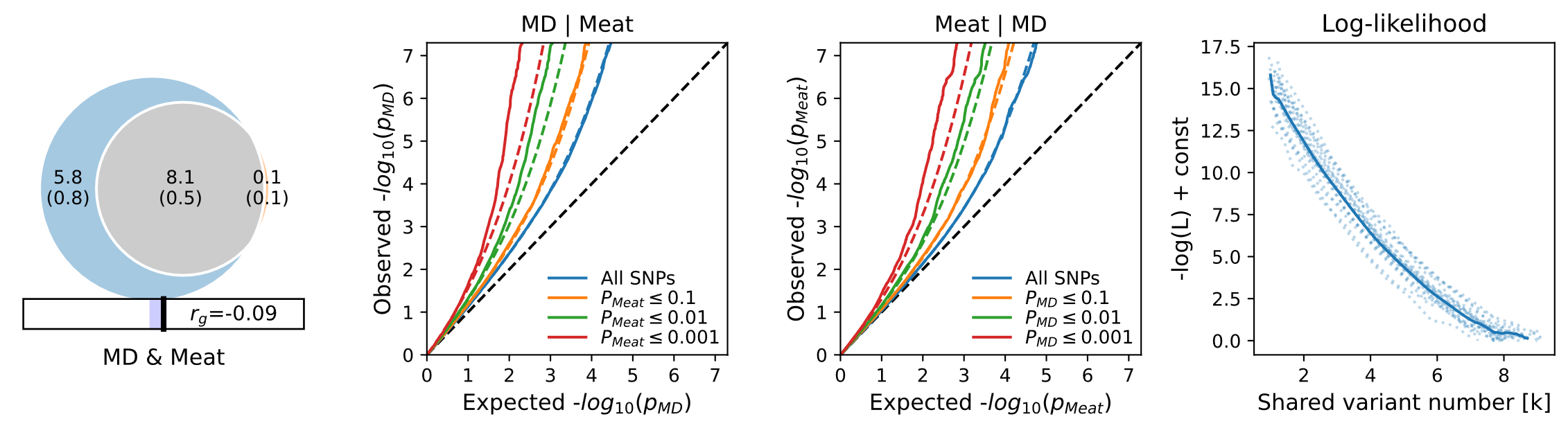

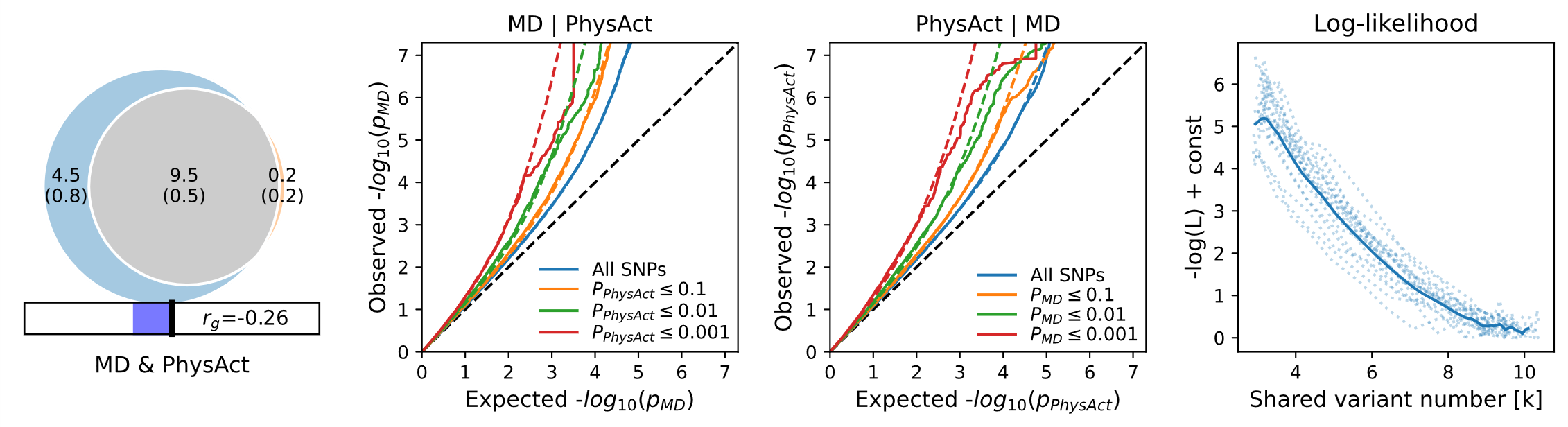

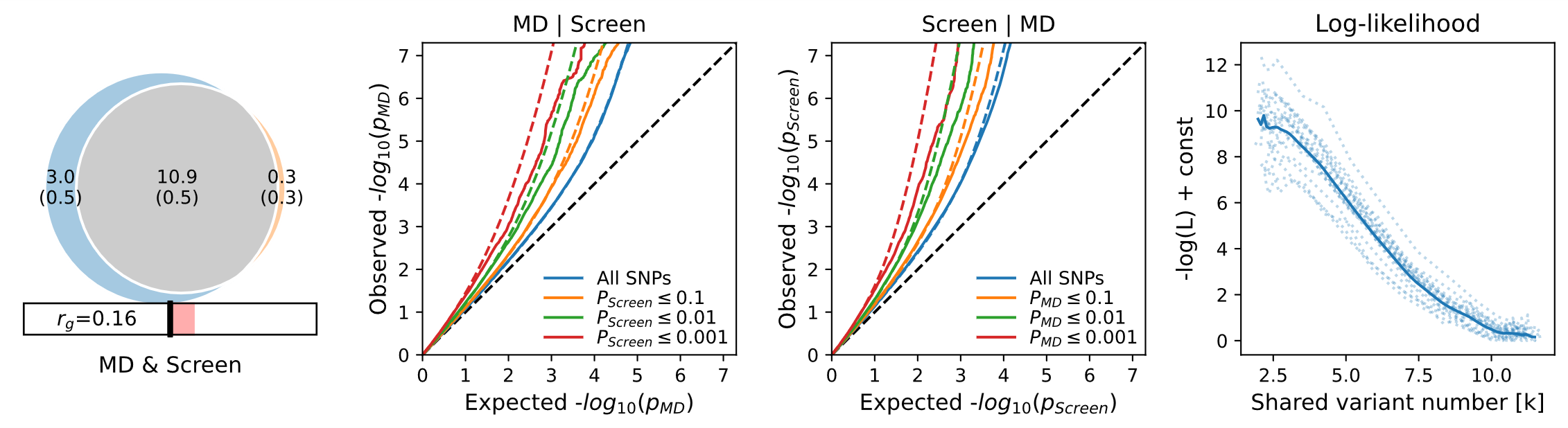

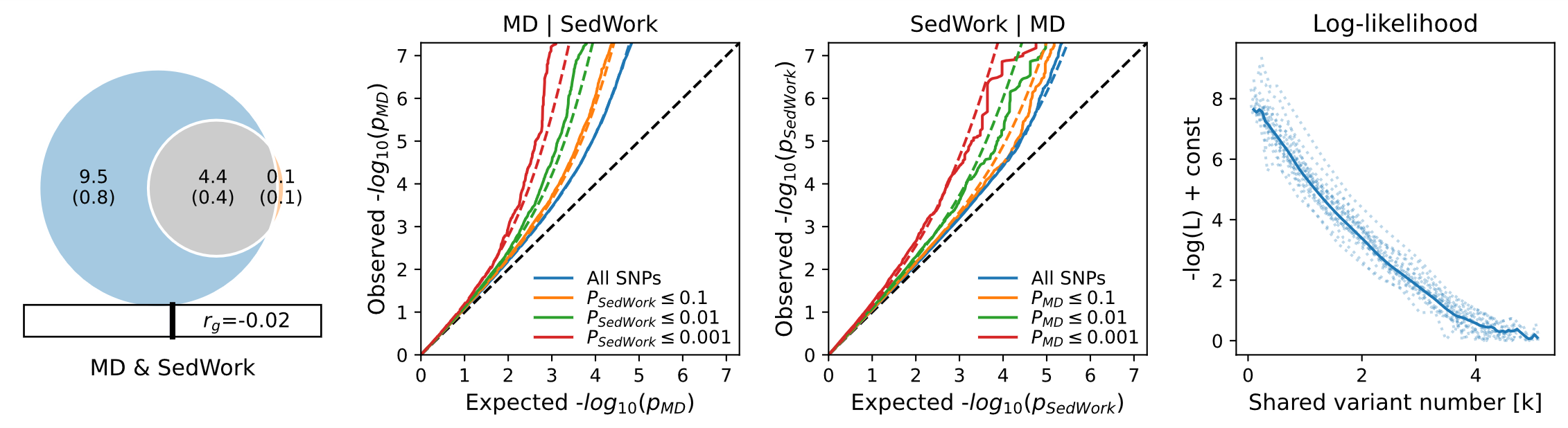


**Fig. S2. Bivariate MiXeR predictions for major depression and lifestyle factors**

Venn Diagrams, conditional Q-Q plots, and negative log-likelihood plot, respectively. Venn diagrams of shared and unique trait-influencing variants, showing polygenic overlap (gray) between schizophrenia (SCZ) (blue) and lifestyle factors (orange). The numbers in the Venn diagram indicate the estimated quantity of trait-influencing variants (in thousands), followed by standard error. SCZ, schizophrenia; Healthy Food, healthy food intake; Meat, meat consumption; PhysAct, Moderate-to-vigorous intensity physical activity; Screen, leisure screen time; SedWork, sedentary behaviour at work. Appearance of the Q-Q plot and negative log-likelihood plot is described below Fig. S2.


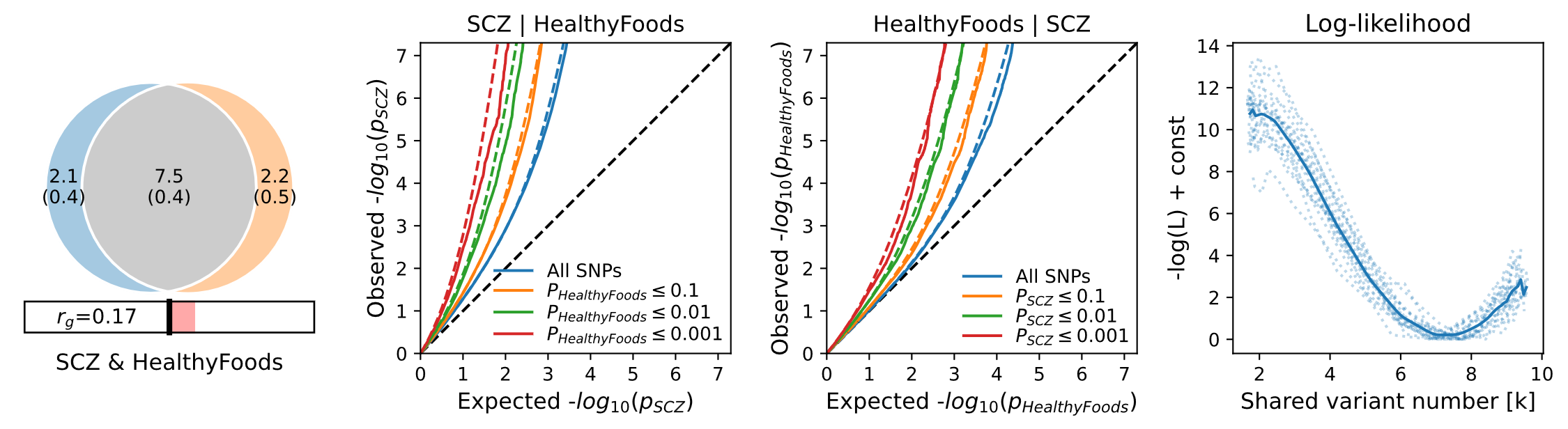

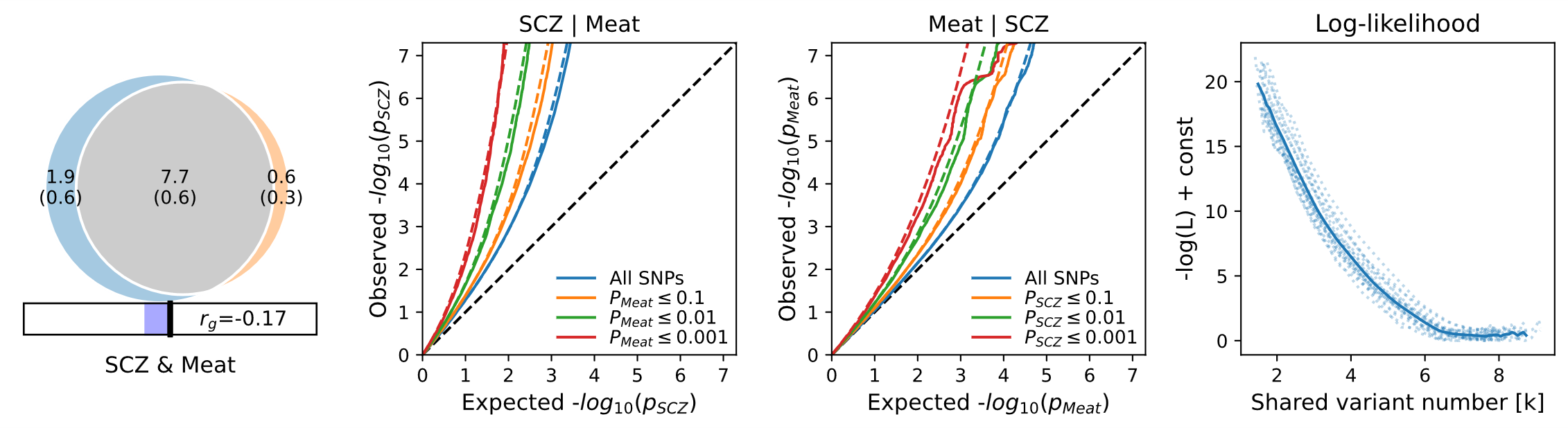

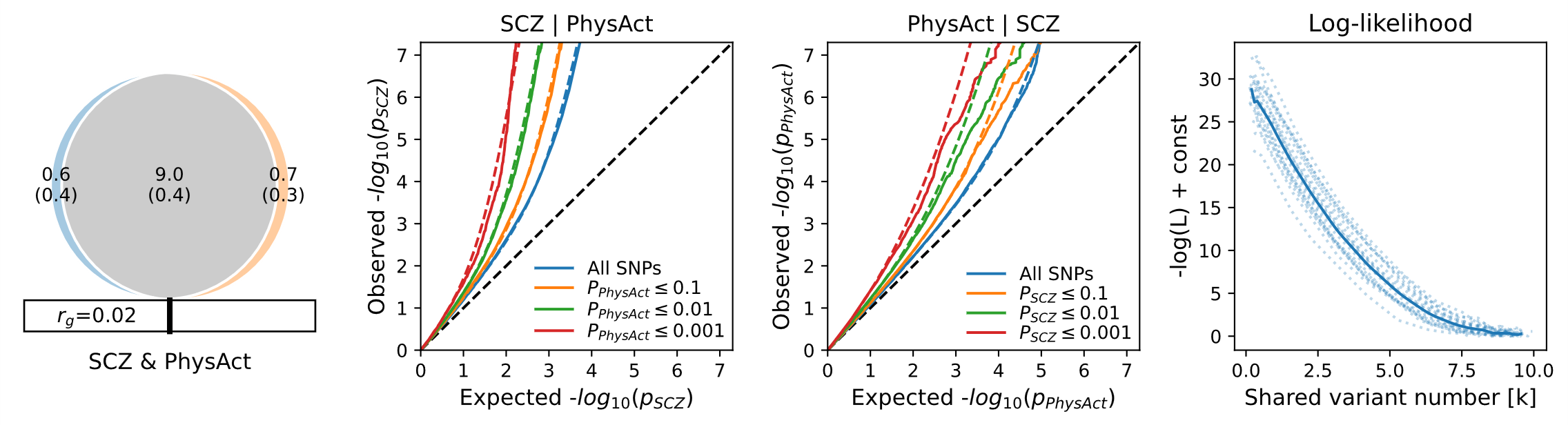

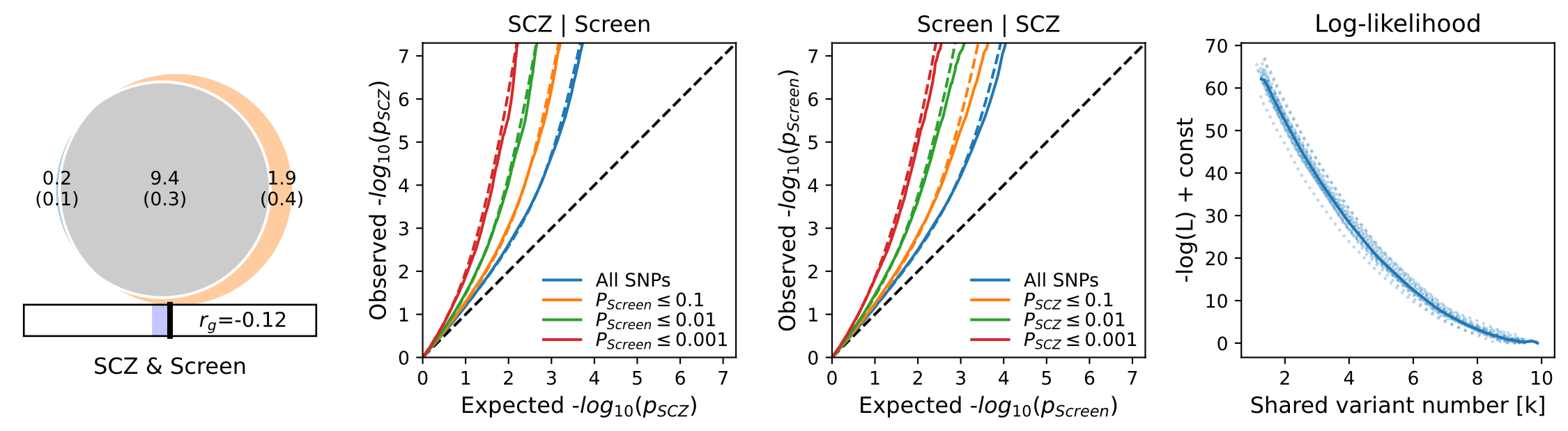

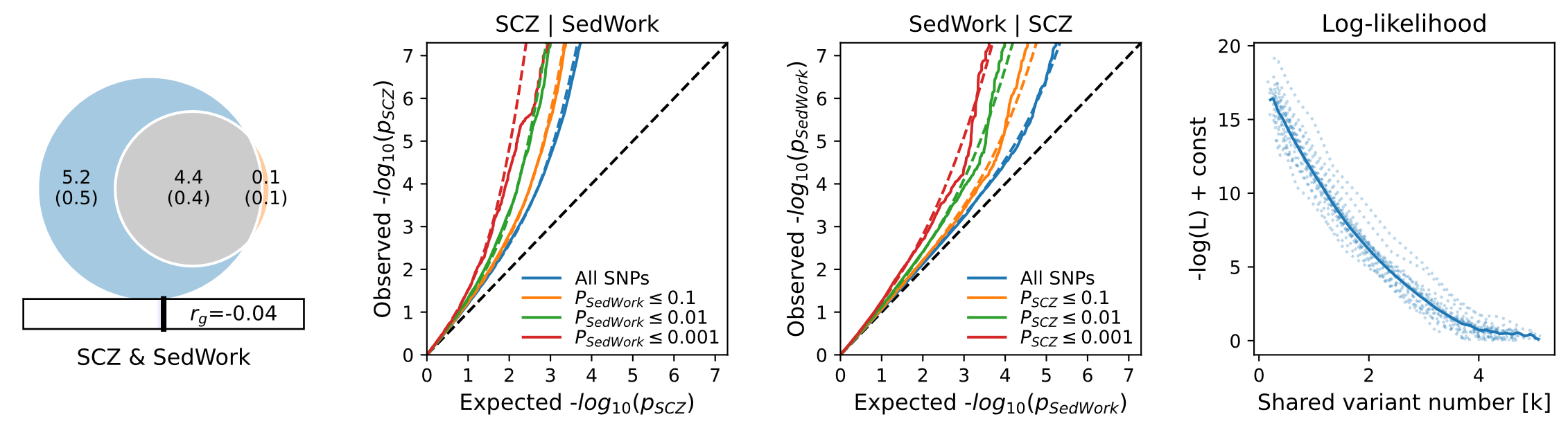


**Fig. S3. Bivariate MiXeR predictions for schizophrenia and lifestyle factors**

Venn Diagrams, conditional Q-Q plots, and negative log-likelihood plot, respectively. Venn diagrams of shared and unique trait-influencing variants, showing polygenic overlap (gray) between bipolar disorder (BIP) (blue) and lifestyle factors (orange). The numbers in the Venn diagram indicate the estimated quantity of trait-influencing variants (in thousands), followed by standard error. BIP, bipolar disorder; Healthy Food, healthy food intake; Meat, meat consumption; PhysAct, Moderate-to-vigorous intensity physical activity; Screen, leisure screen time; SedWork, sedentary behaviour at work. Appearance of the Q-Q plot and negative log-likelihood plot is described below Fig. S2.


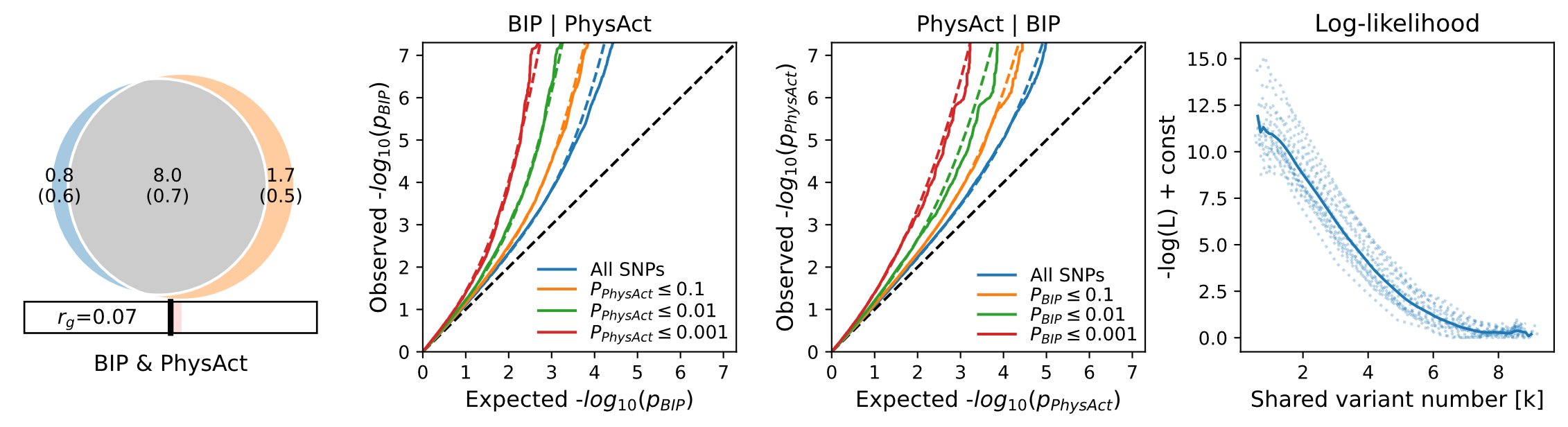

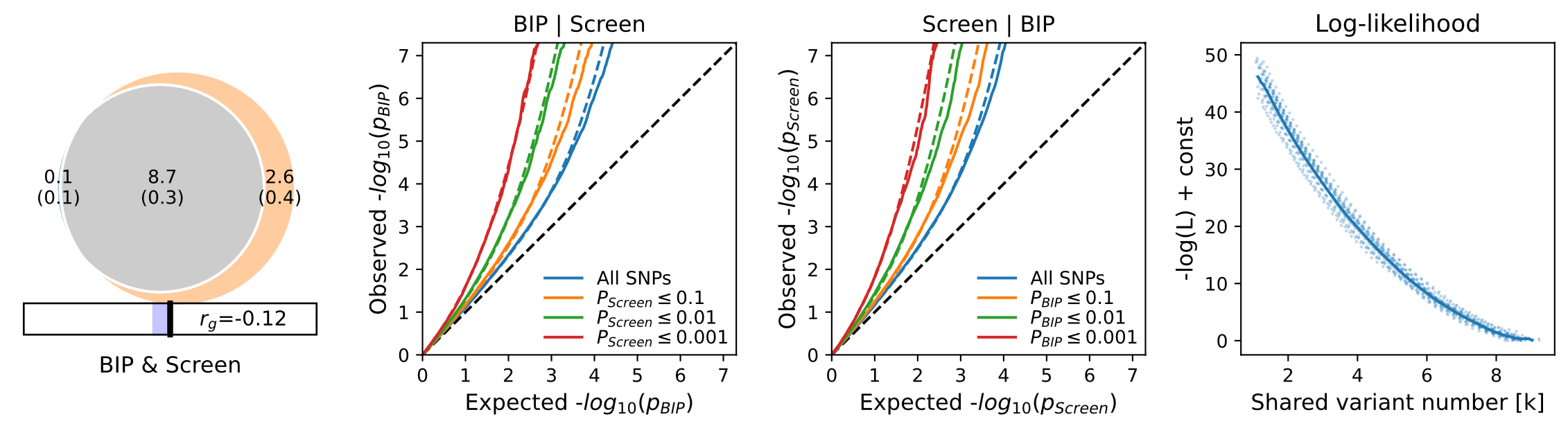

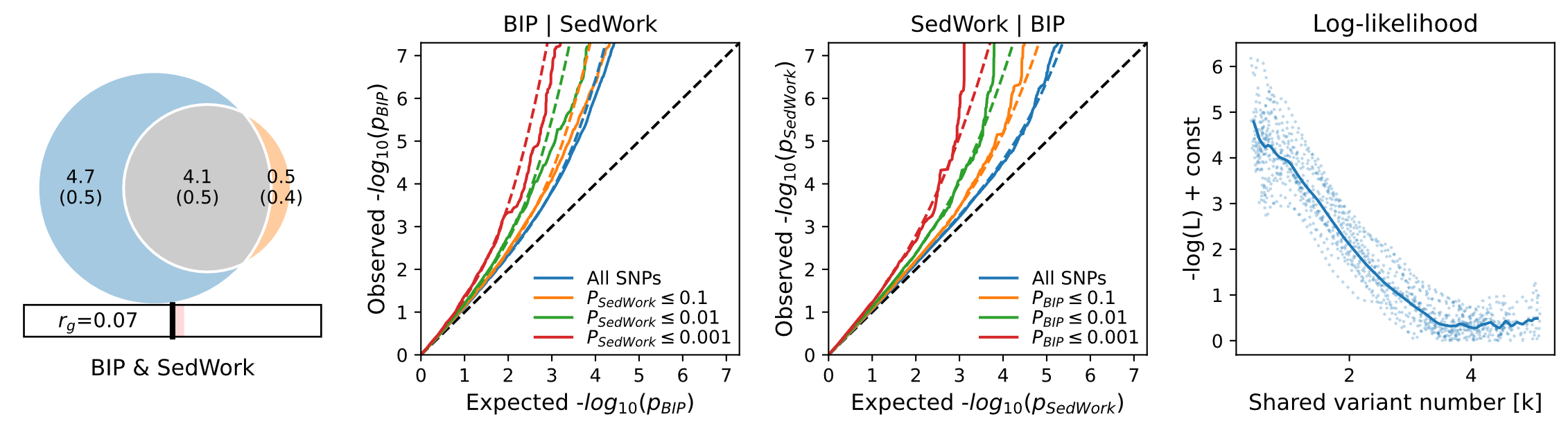

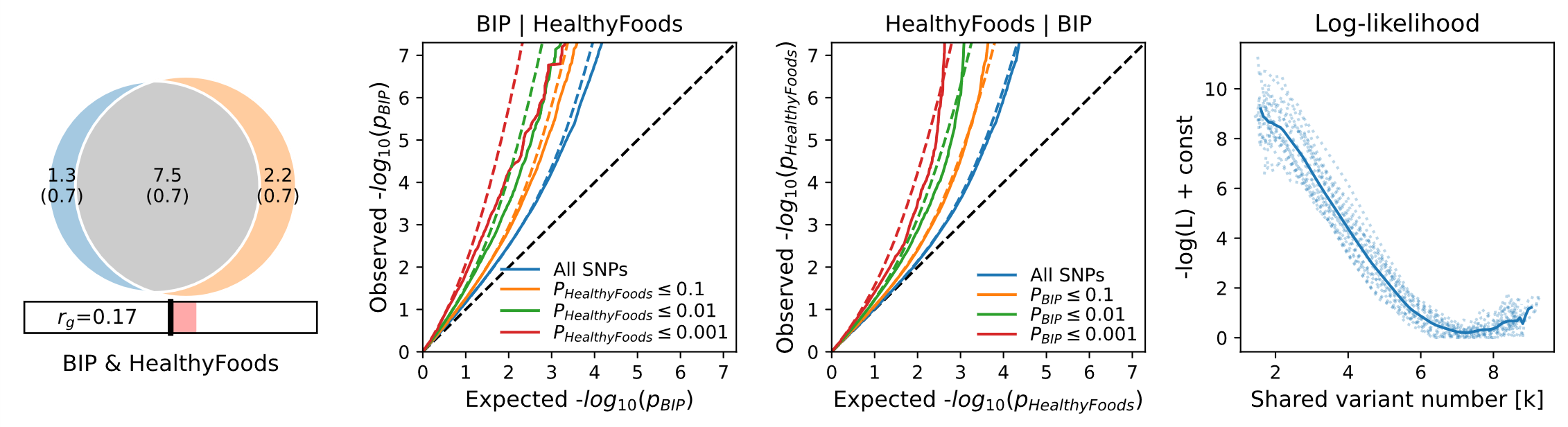

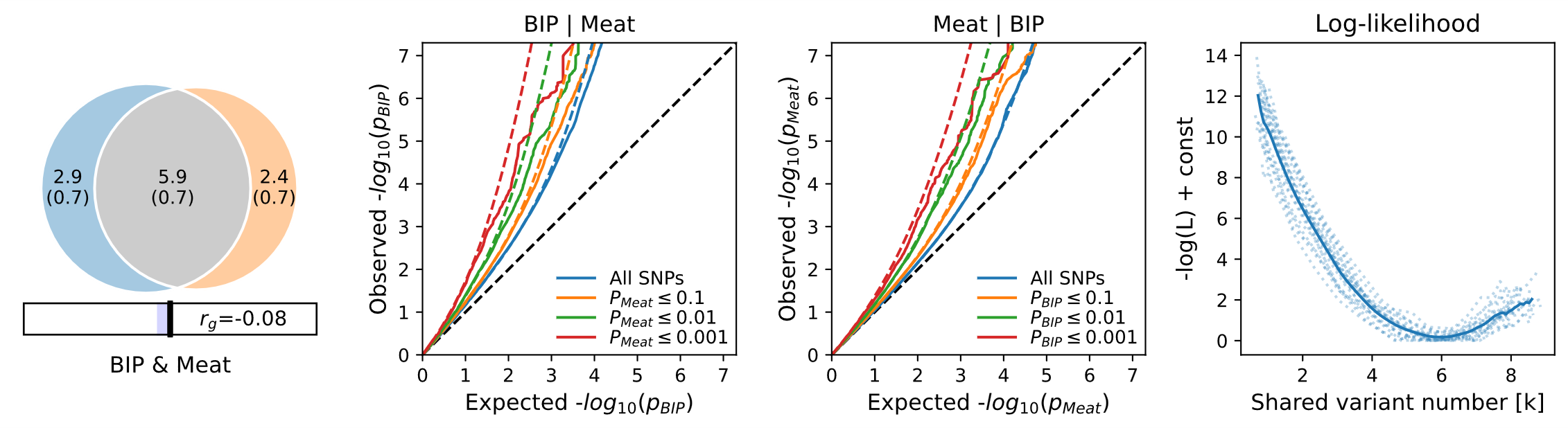


**Fig. S4. Bivariate MiXeR predictions for bipolar disorder and lifestyle factors**


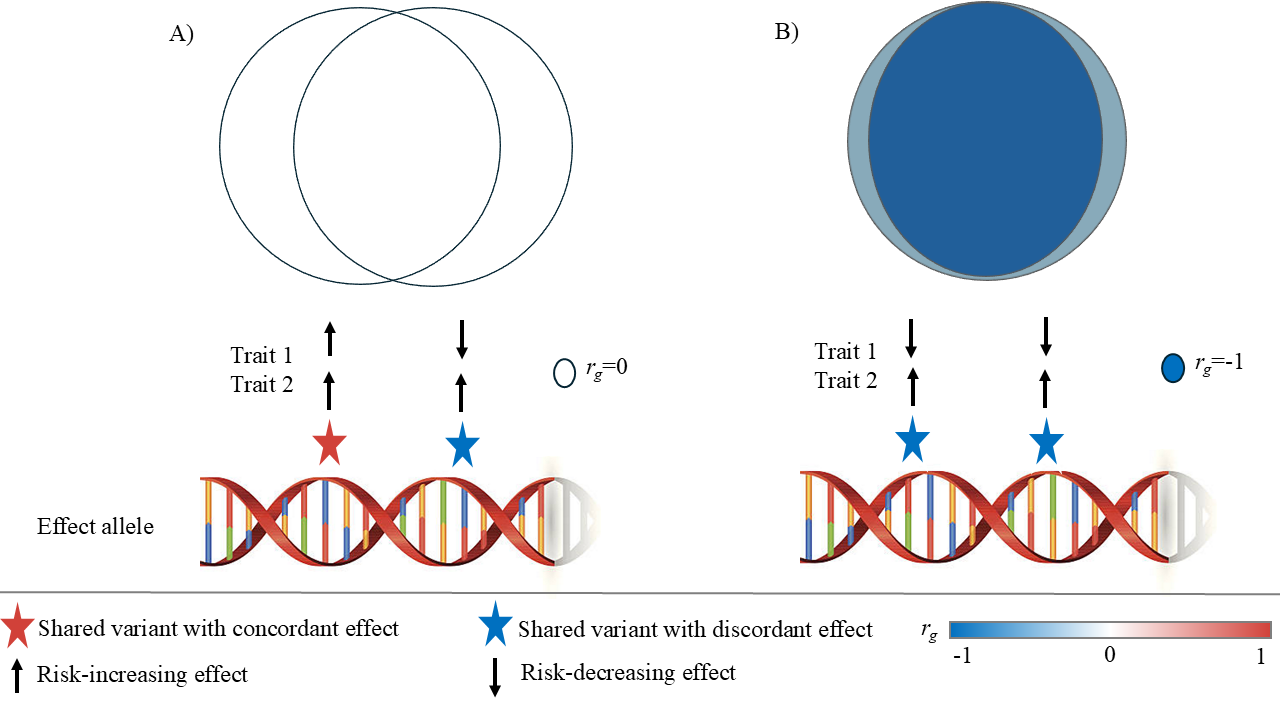


Panel A illustrates mixed effect directions of the shared variants, resulting in zero genetic correlation. Panel B illustrates discordant allelic effects, yielding negative genetic correlations. The figure is adapted from Hindley et al.^1^

**Fig. S5. Concepts of the MiXeR model: Polygenic overlap despite minimal or negative genetic correlations (*r_g_*)**


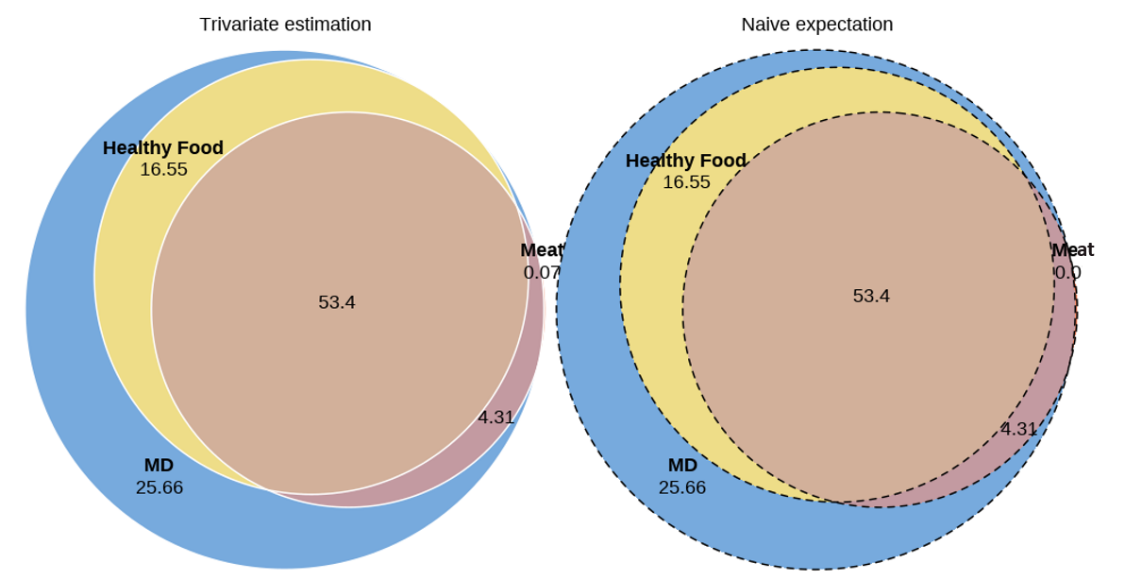


**A)**

**B)**


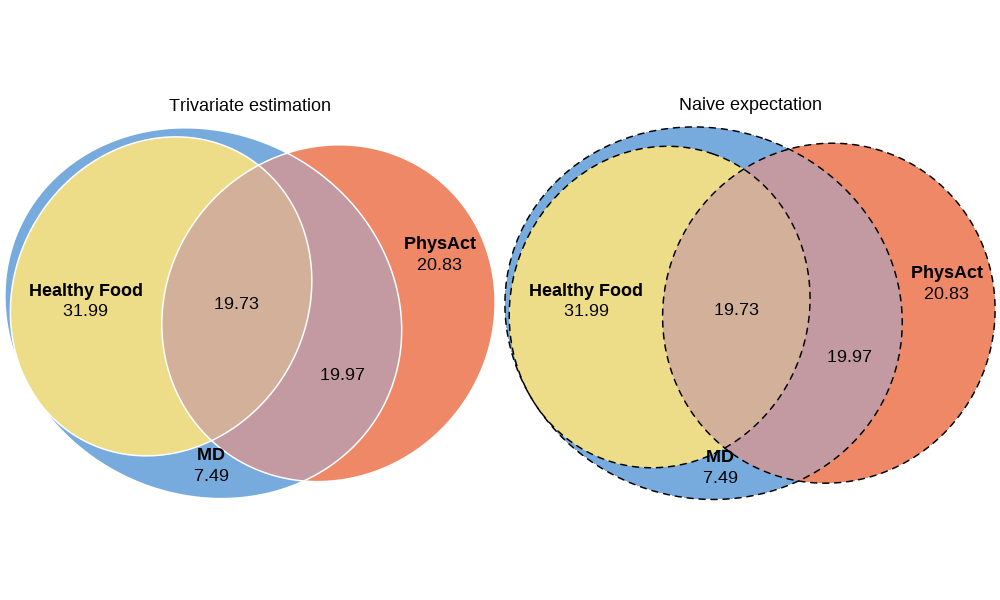

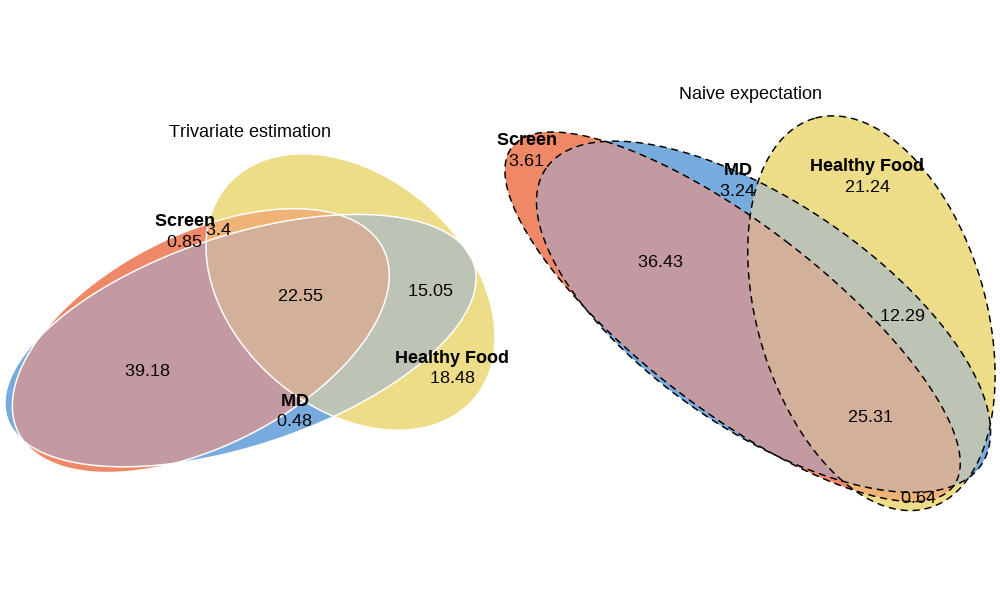

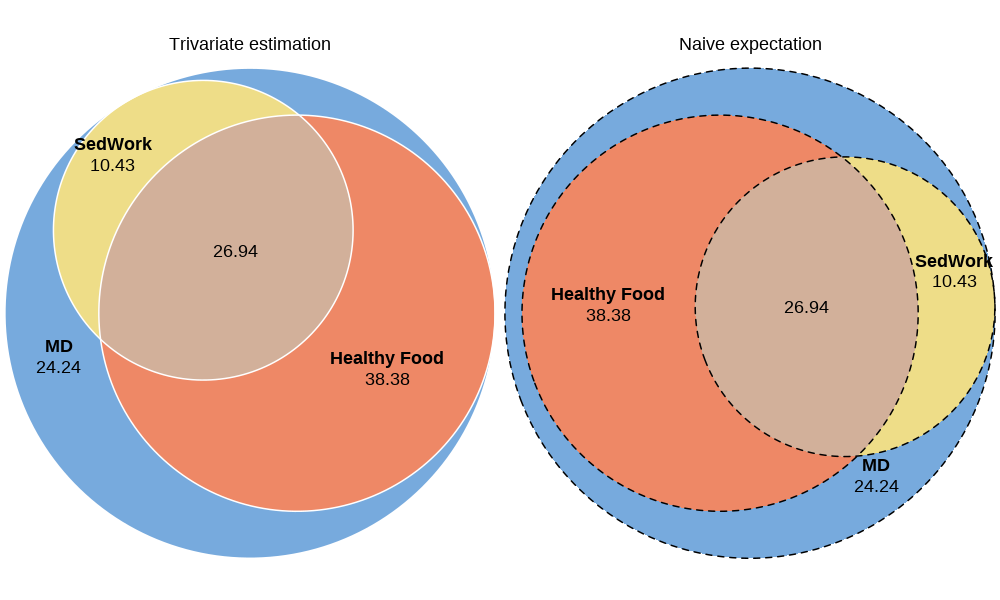


**Fig. S6. Trivariate MiXeR: Genetic overlap between severe mental disorders and two lifestyle factors**


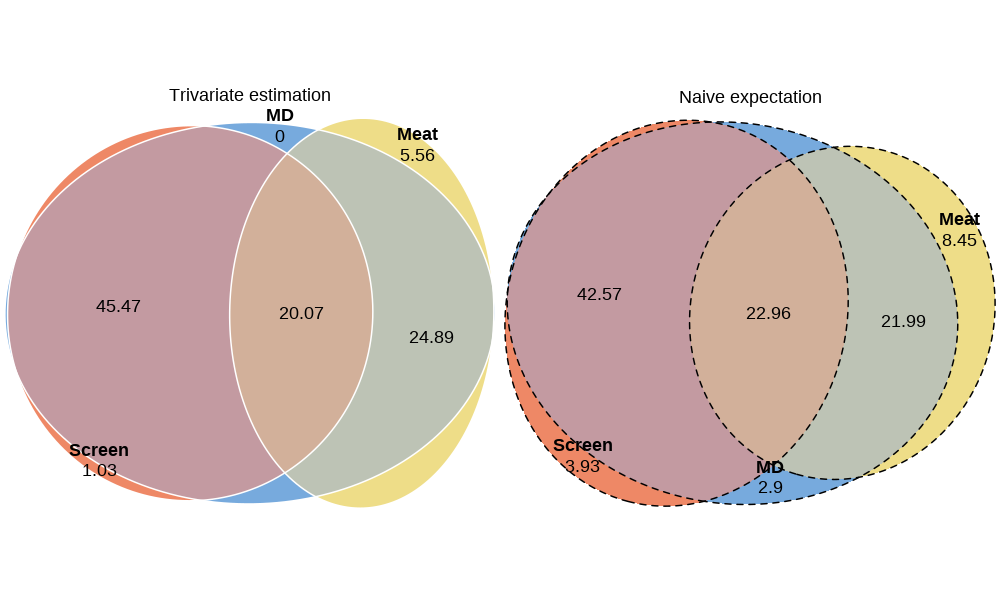

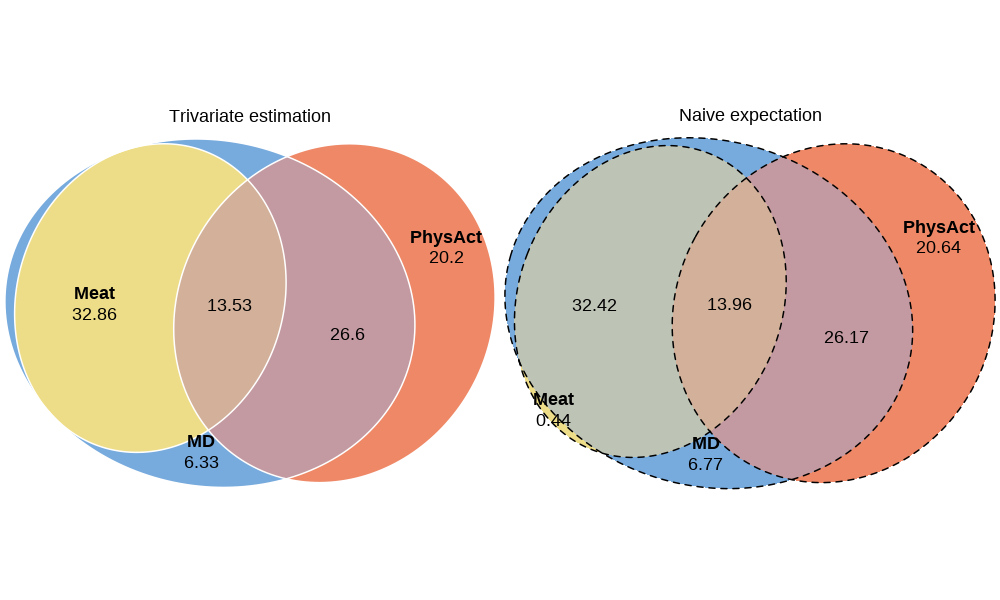

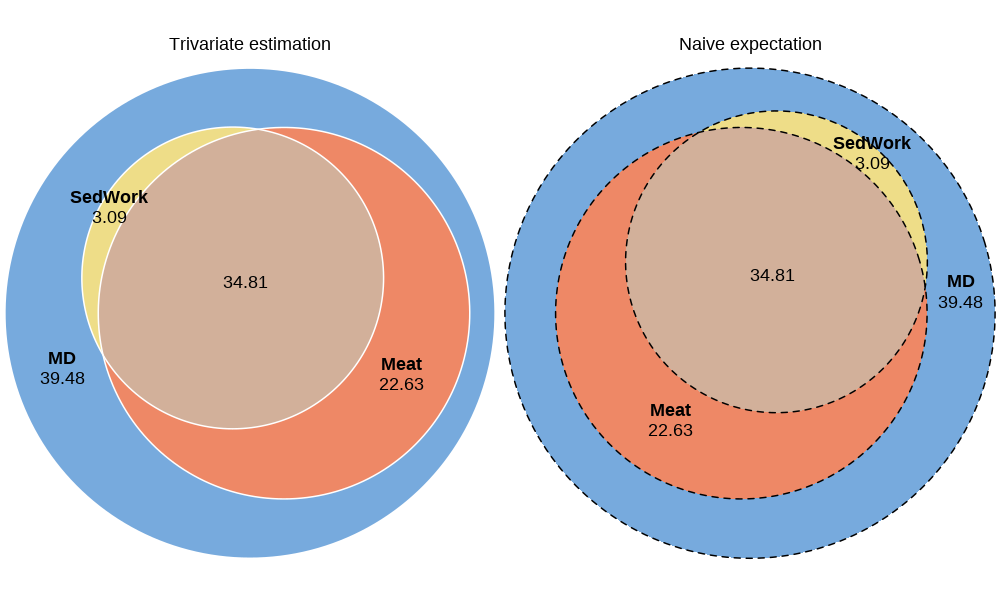


**A)**

**B)**


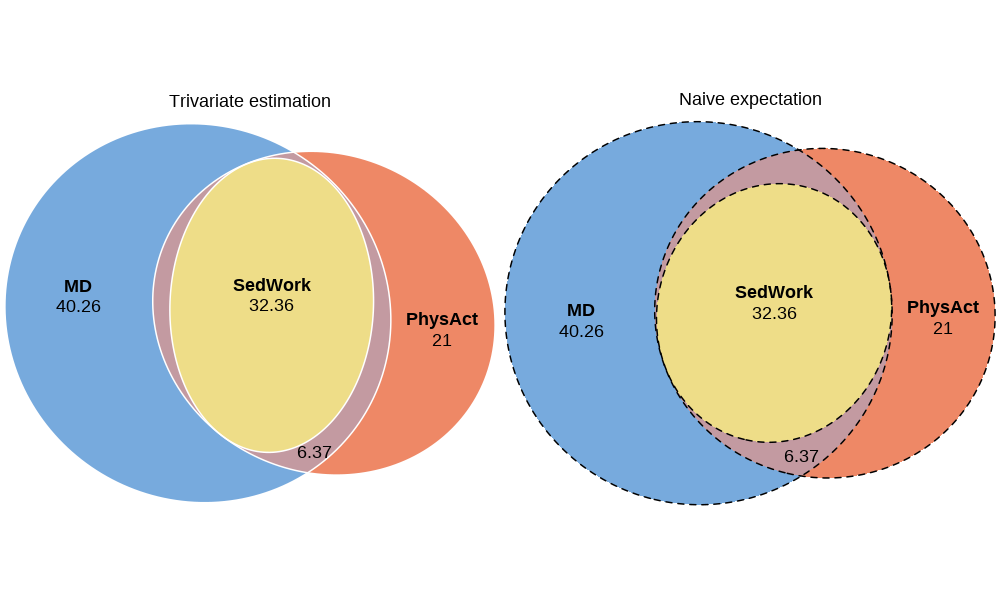

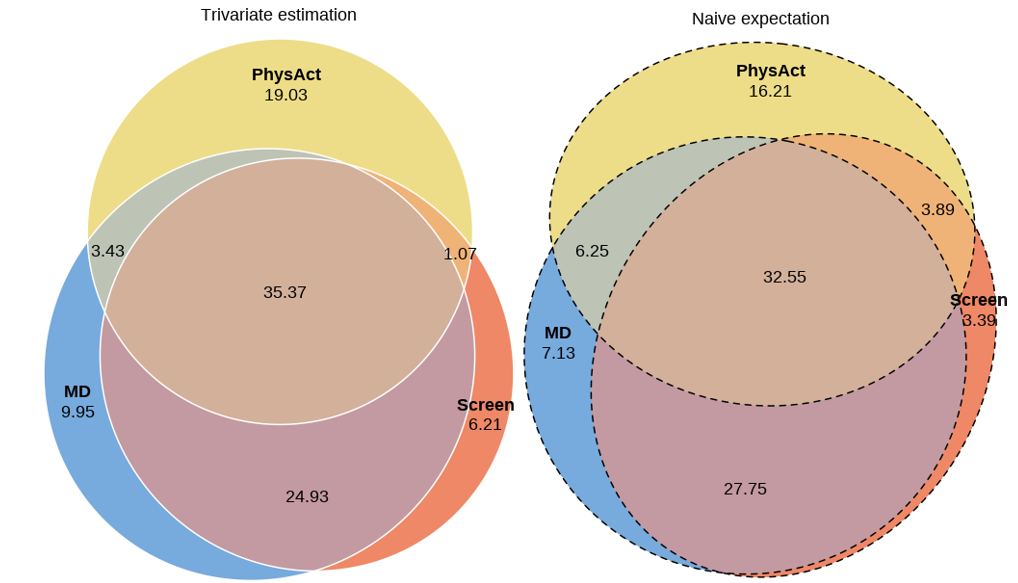

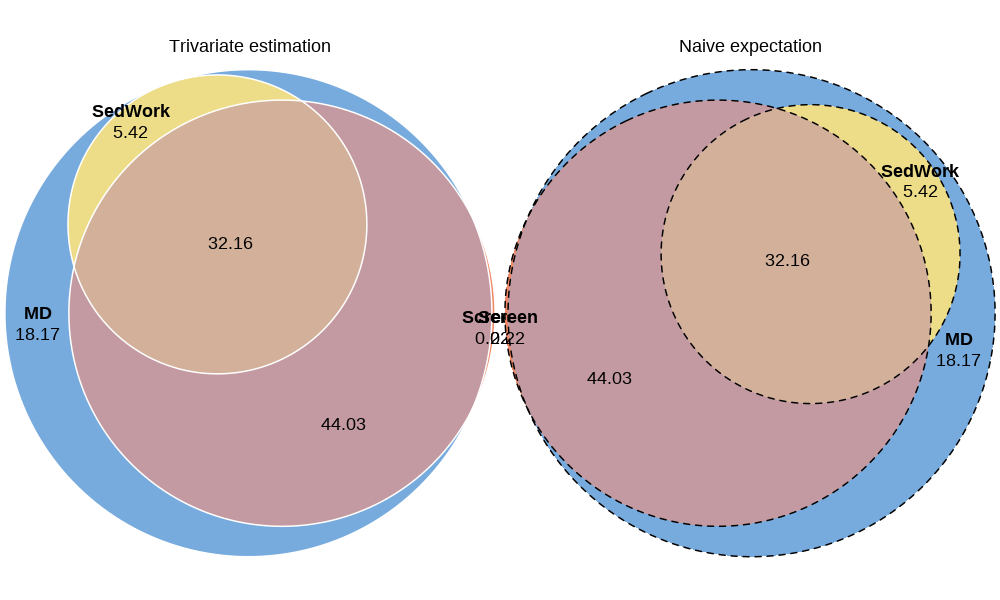


**A)**

**B)**


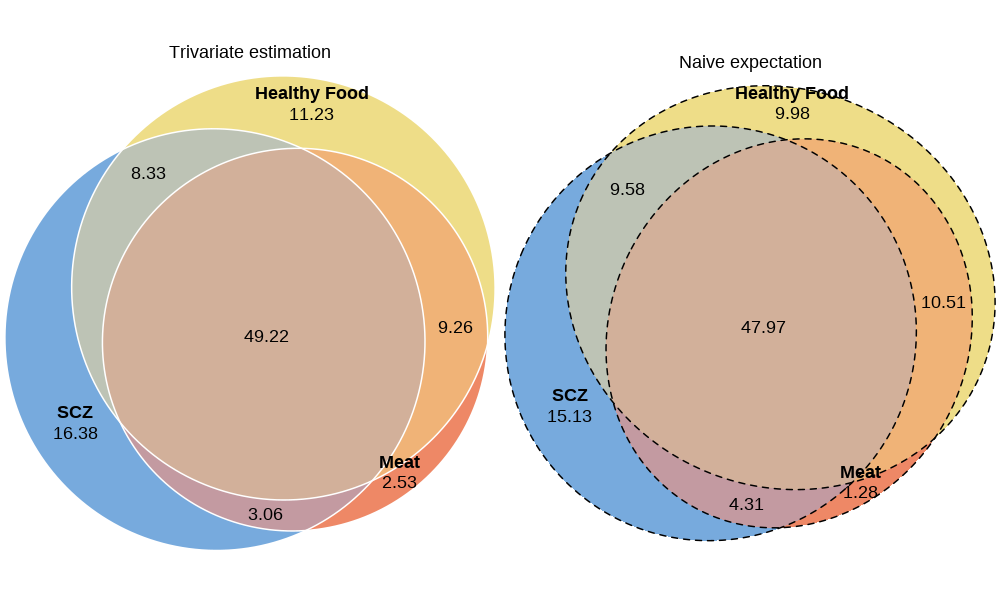


**A)**

**B)**


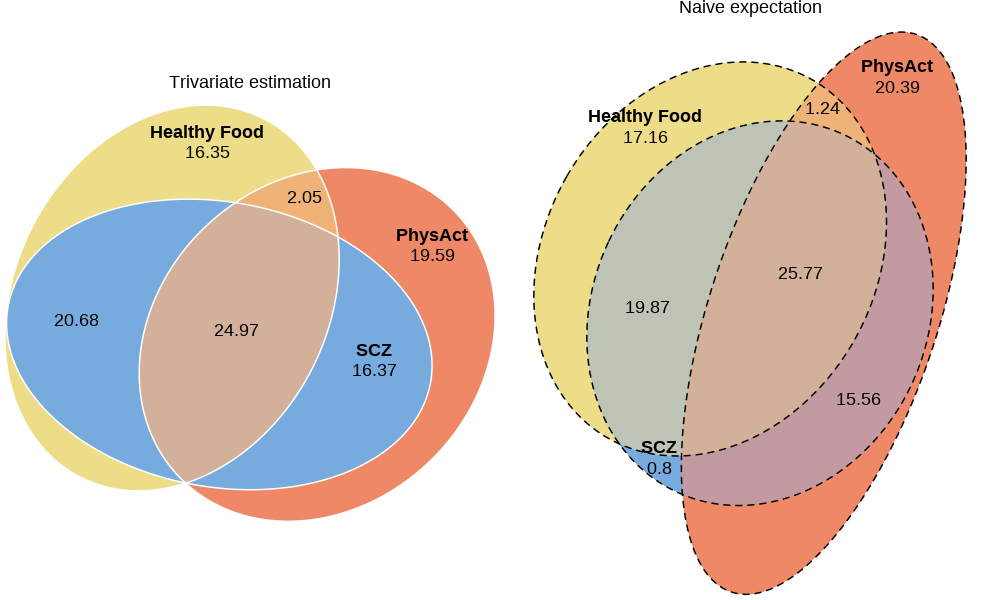


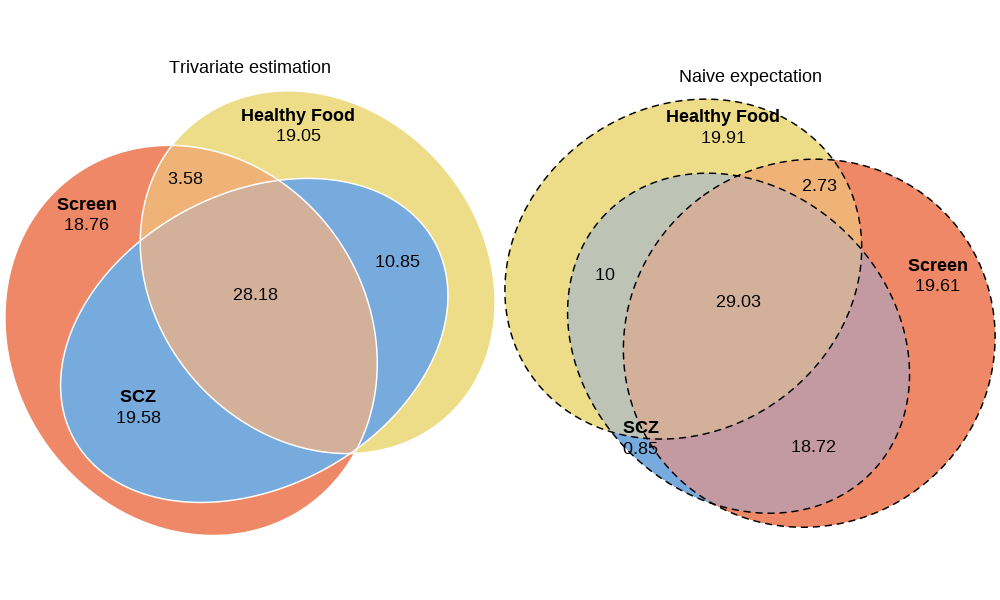


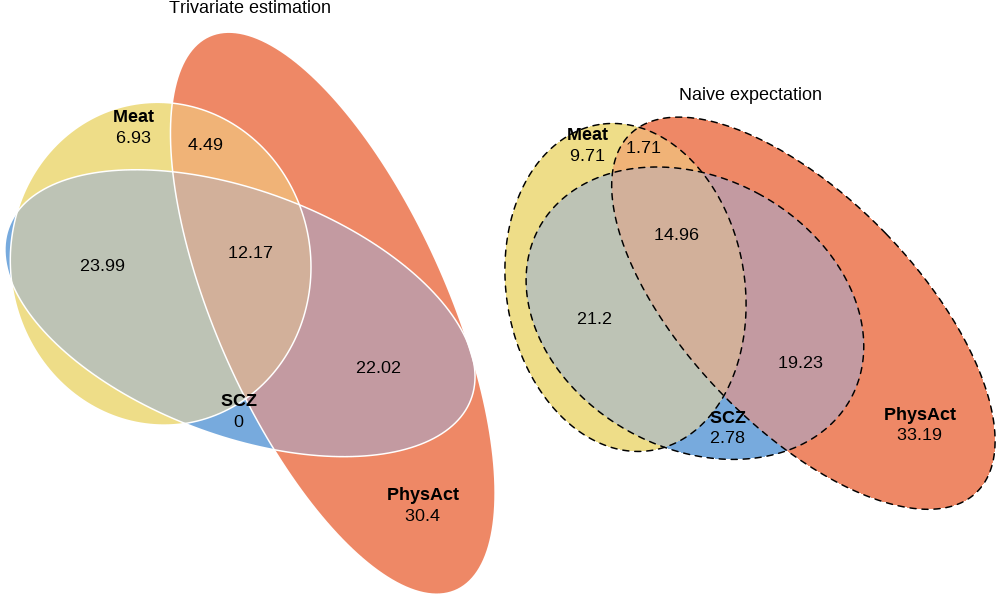

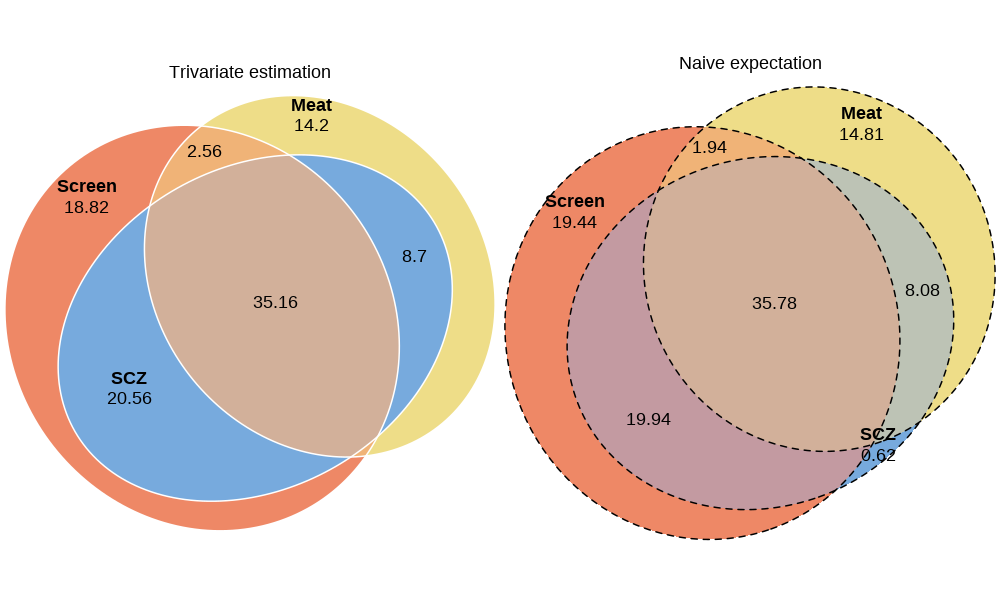

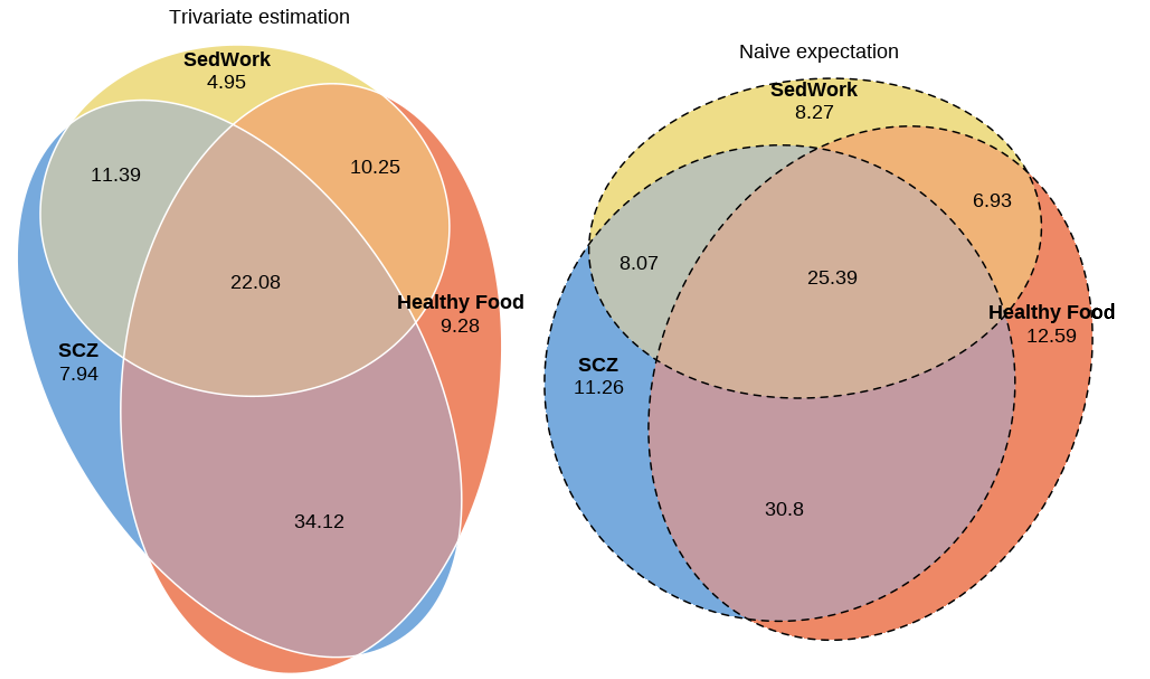


**A)**

**B)**


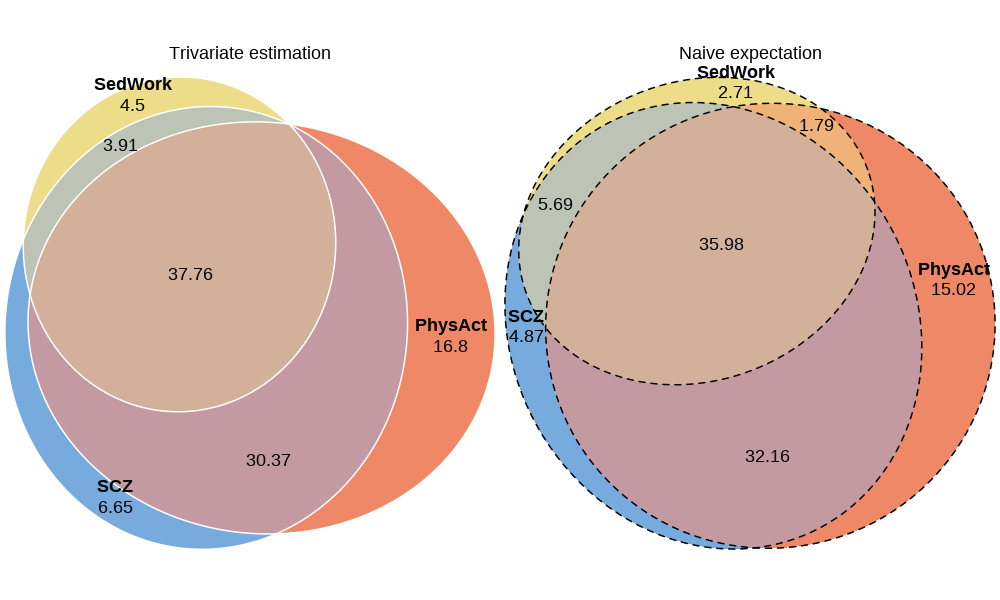

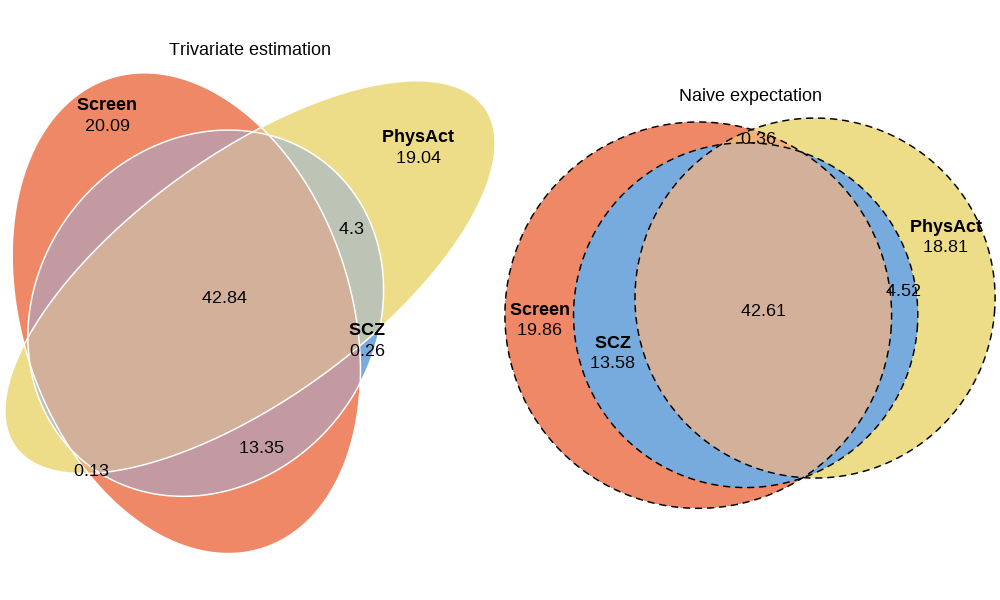

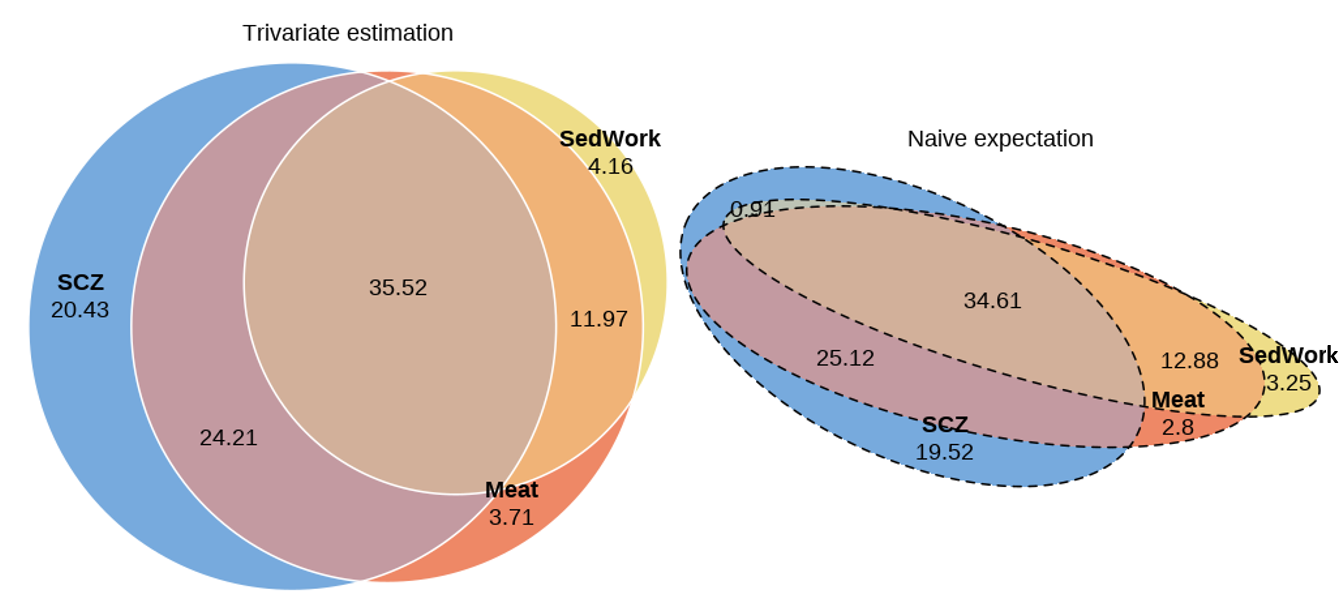


**A)**

**B)**

**A)**

**B)**


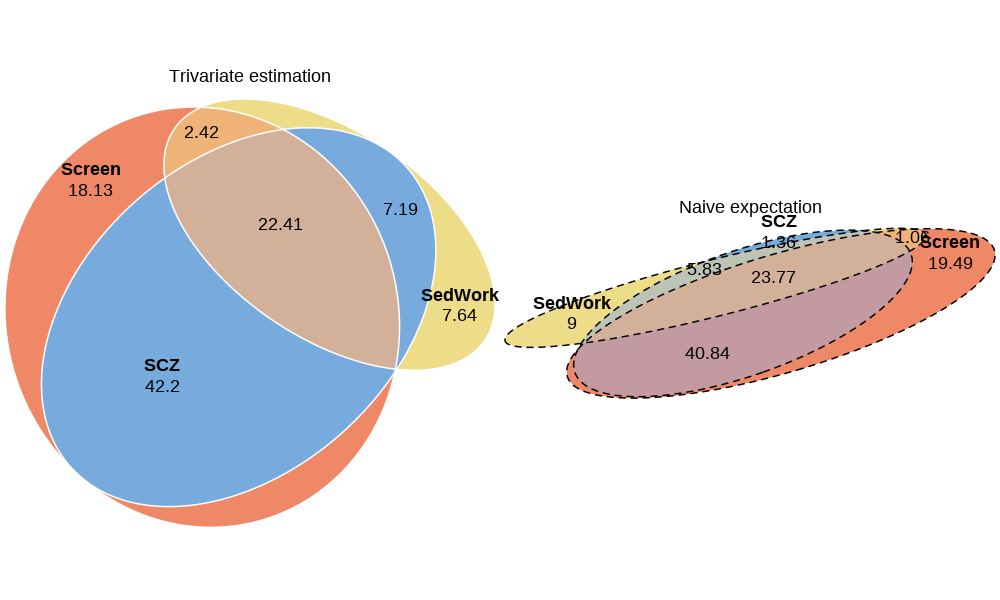

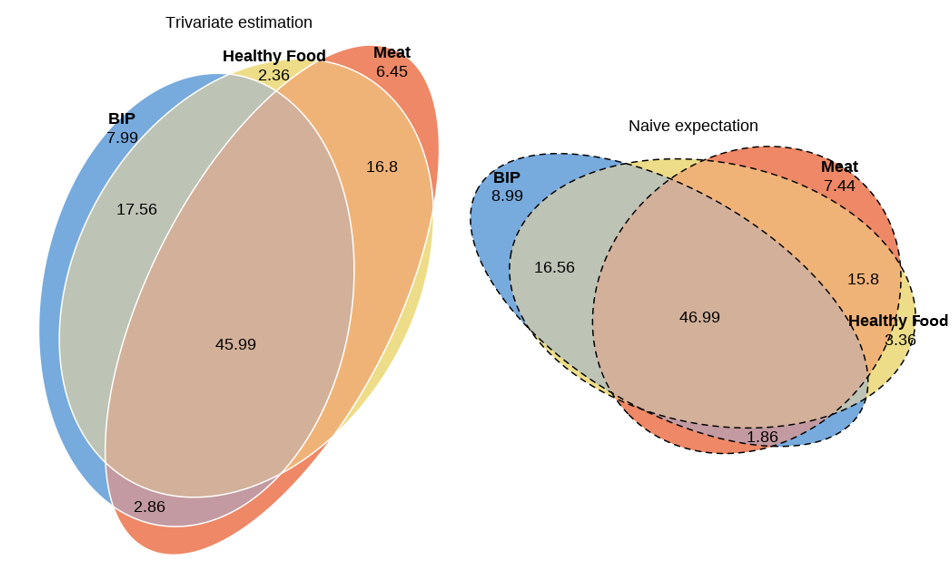


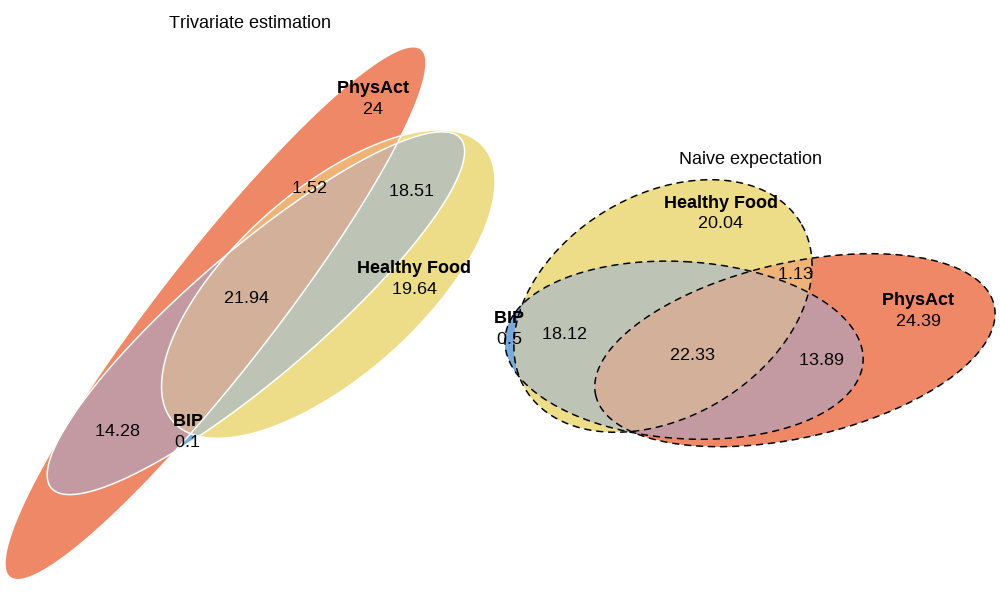


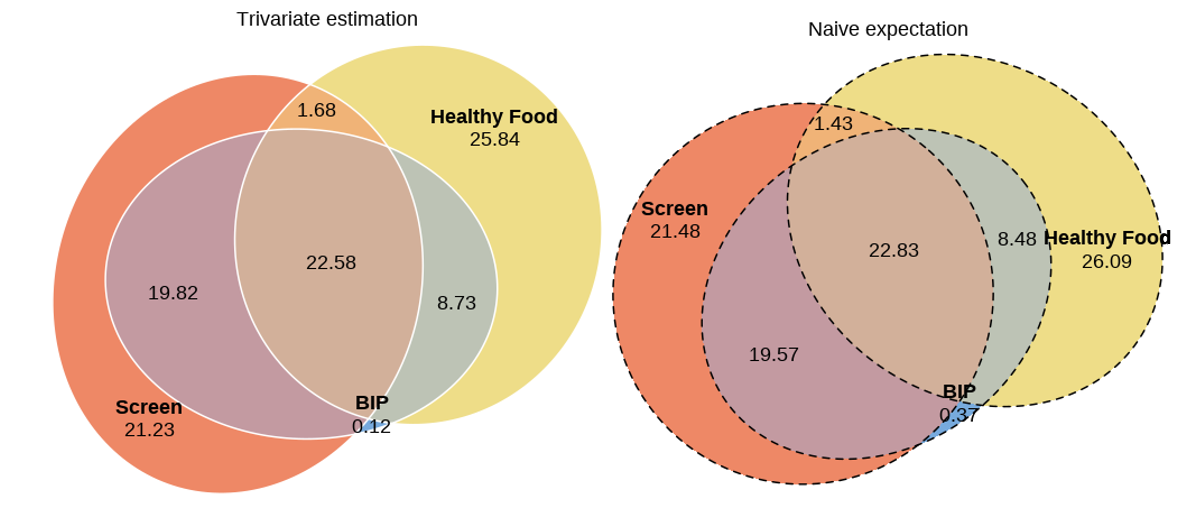

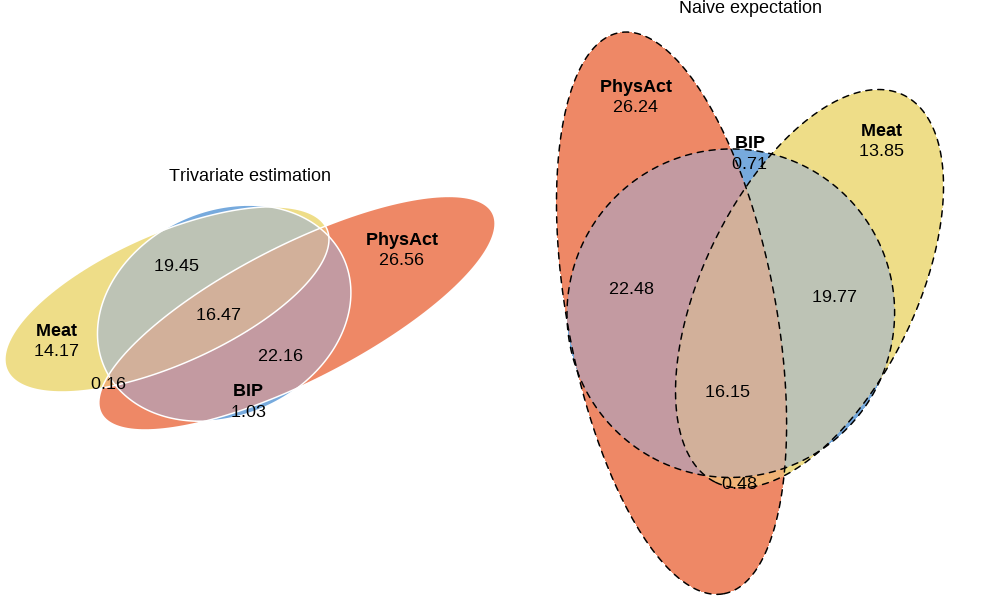

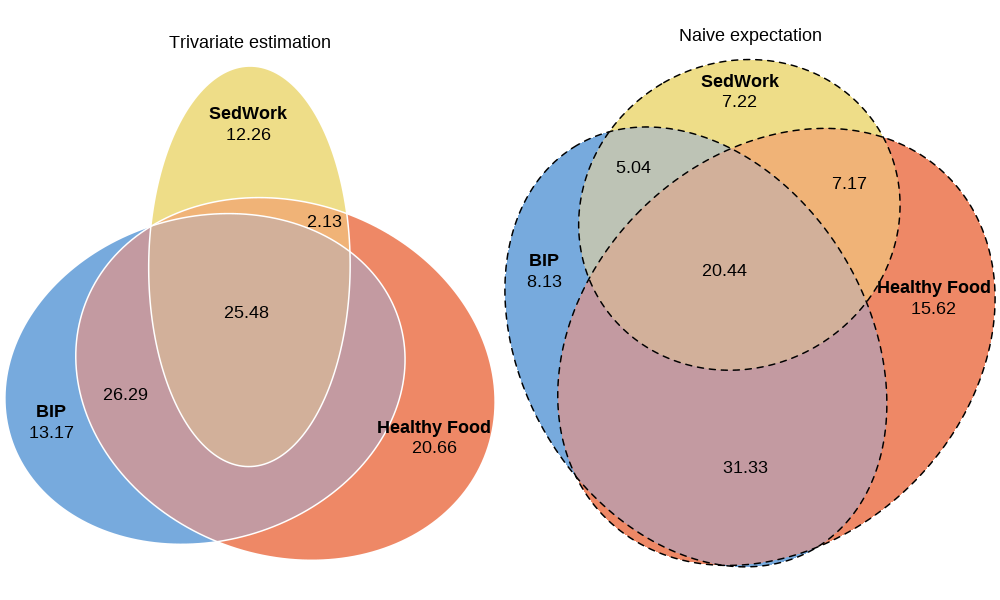


**A)**

**B)**


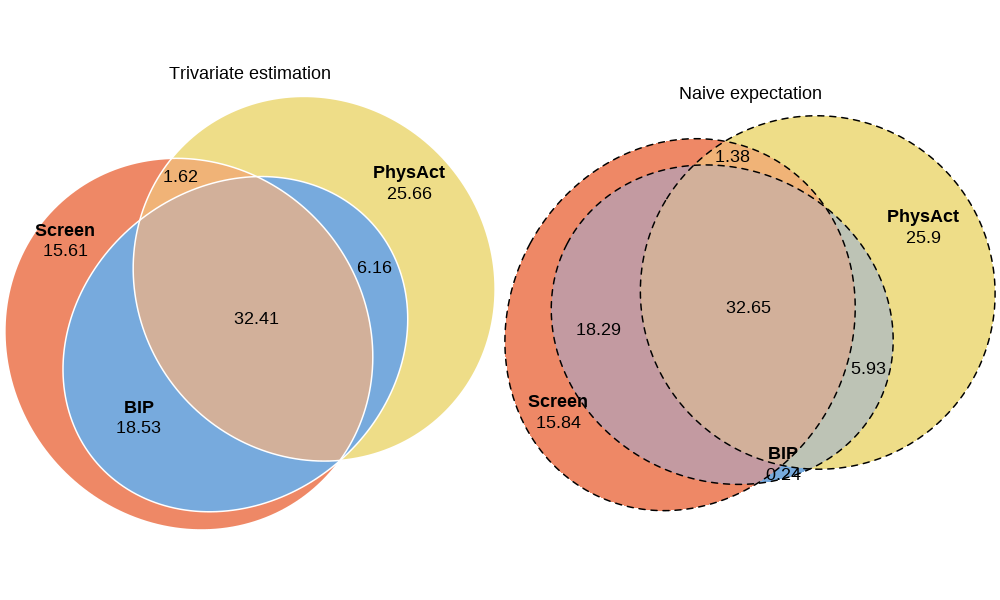

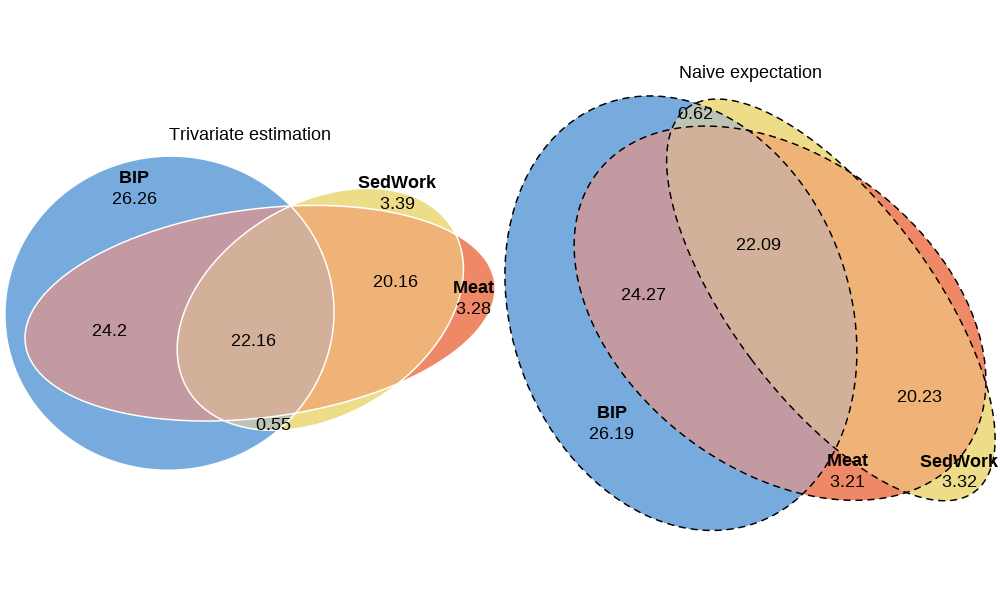

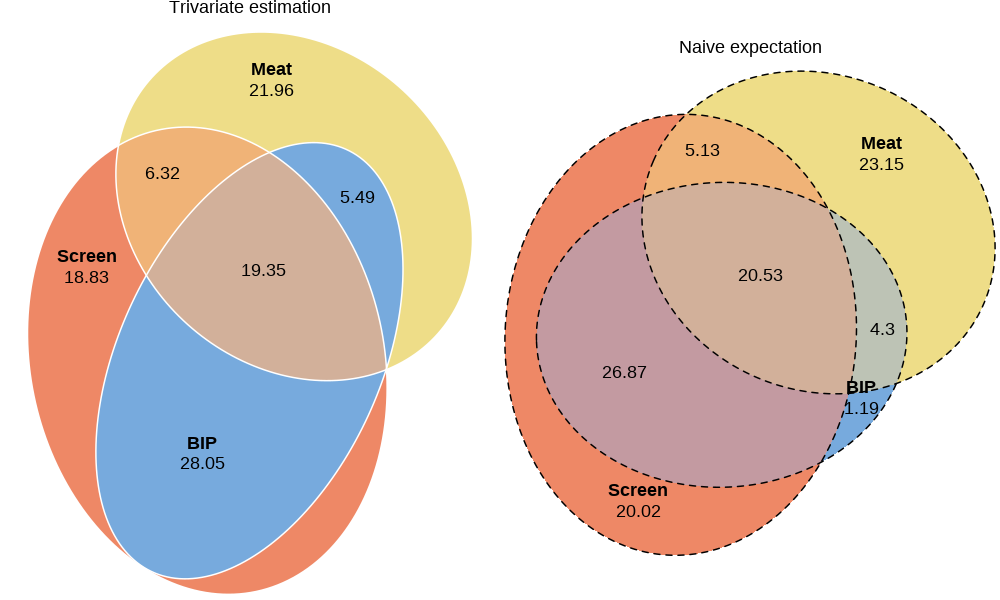


**A)**

**B)**

**A)**

**B)**


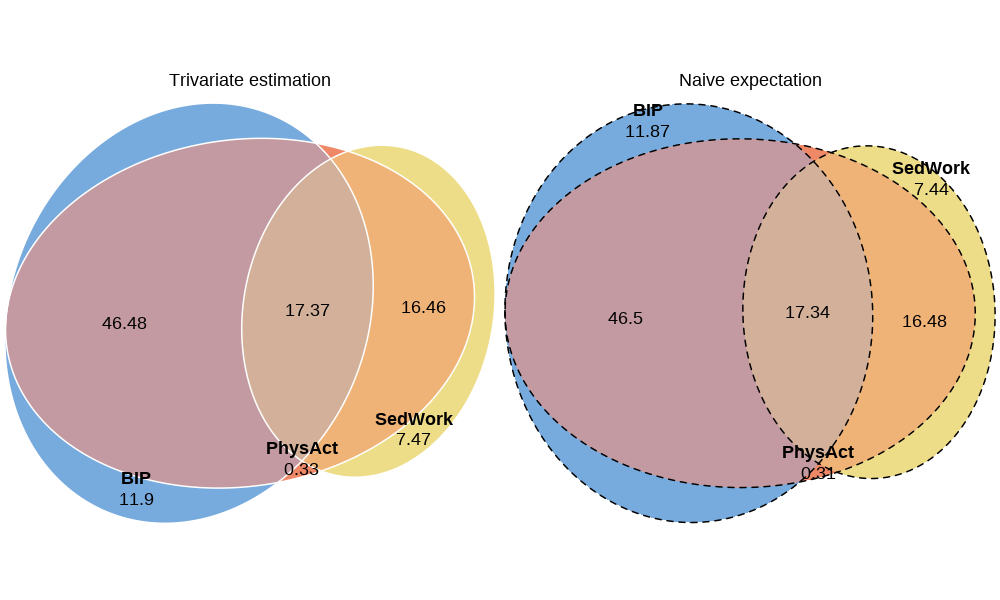


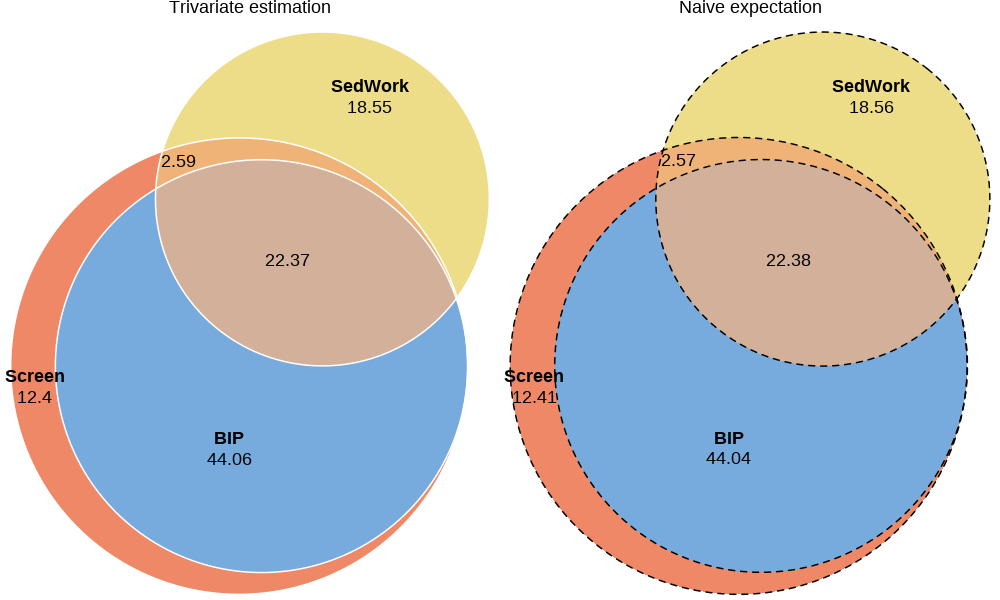


Estimates by *trivariate* *MiXeR* (column A) compared to naïve expectation following the principle of maximum entropy, i.e., deduced from *three bivariate analyses*) (column B). The pattern of genetic overlap between each triad of phenotypes demonstrates the differences between trivariate MiXeR estimates and naïve expectation from bivariate MiXeR. For each triad of phenotypes (within each row), for every area of each diagram, its percentage with respect to the combined total area of all three phenotypes in the estimated diagram (column A) is shown, i.e., percentages within each diagram in column A add up to 100 and percentages within each row are directly comparable. Since percentages in column B are also given with respect to the combined total area of the corresponding diagram in column A, the sum of percentages in column B is not necessarily equal to 100. MD, major depression; SCZ, schizophrenia; BIP, bipolar disorder; Healthy Food, healthy food intake; Meat, meat consumption; PhysAct, Moderate-to-vigorous intensity physical activity; Screen, leisure screen time; SedWork, sedentary behaviour at work.


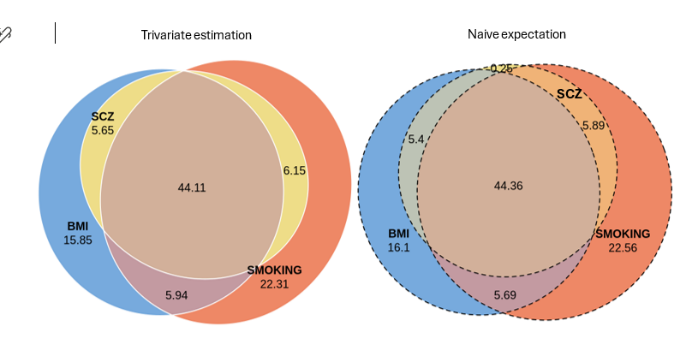

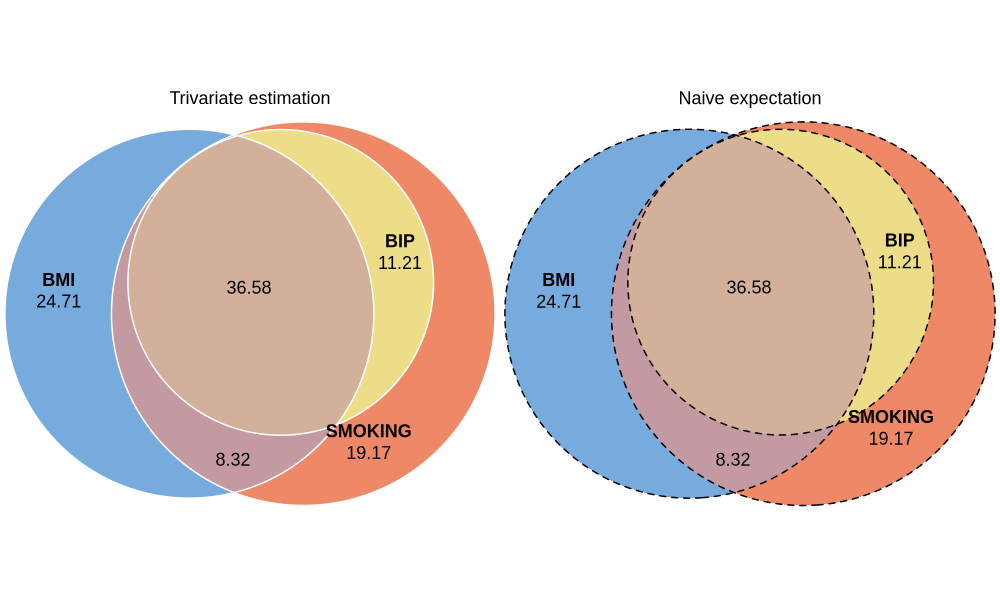


Estimates by *trivariate* *MiXeR* (column A) compared to naïve expectation following the principle of maximum entropy, i.e., deduced from *three bivariate analyses*) (column B). SCZ, schizophrenia; BMI, body mass index.

**A)**

**B)**

**Fig. S7. Trivariate MiXeR: Genetic overlap between schizophrenia, body mass index and smoking**

**A)**

**B)**


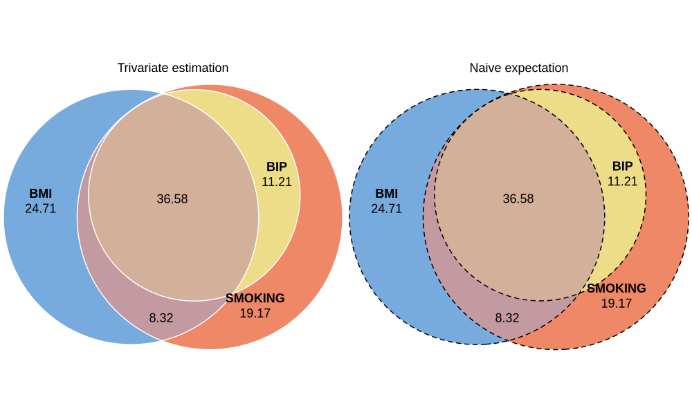


Estimates by *trivariate* *MiXeR* (column A) compared to naïve expectation following the principle of maximum entropy, i.e., deduced from *three bivariate analyses*) (column B). BIP, bipolar disorder; BMI, body mass index.

**Fig. S8. Trivariate MiXeR: Genetic overlap between bipolar disorder, body mass index and smoking.**

**Fig. S9. The conditional Q-Q plots for severe mental disorders conditional on lifestyle**


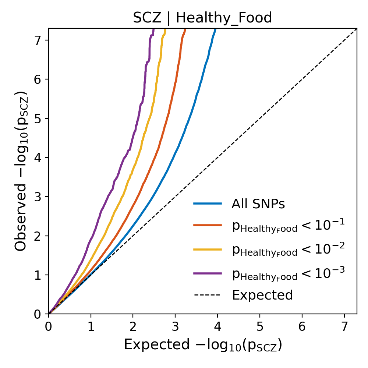

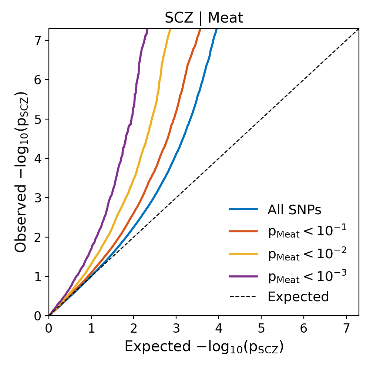

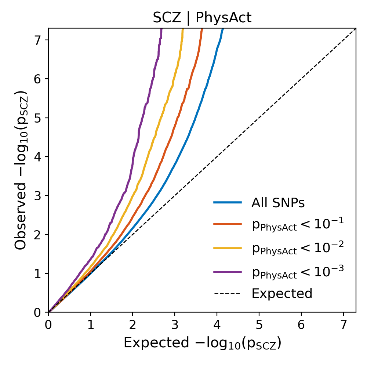

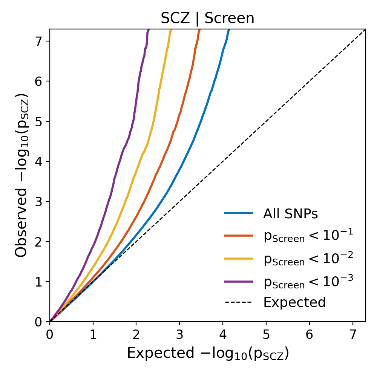

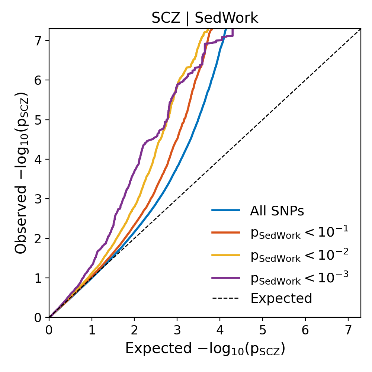

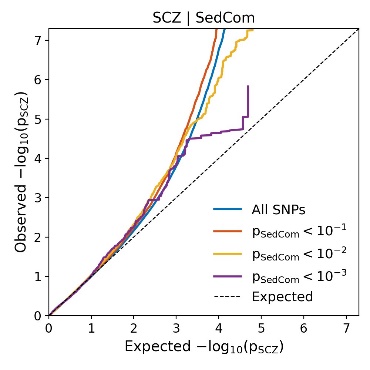

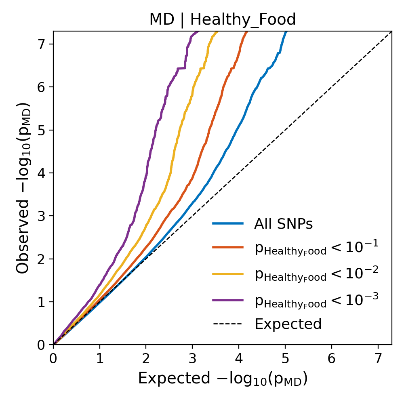

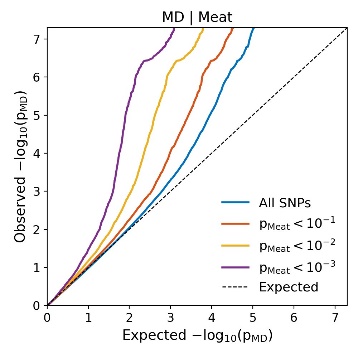

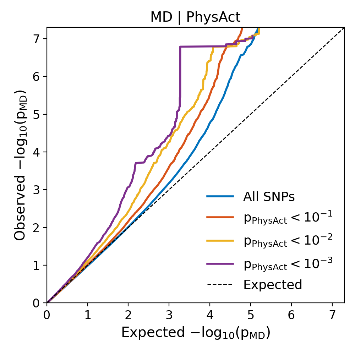

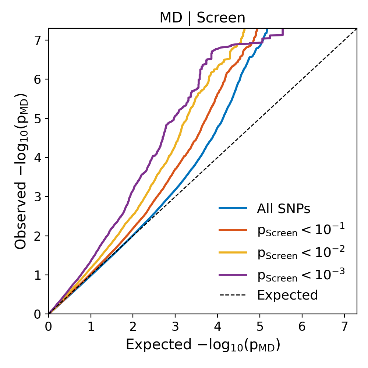

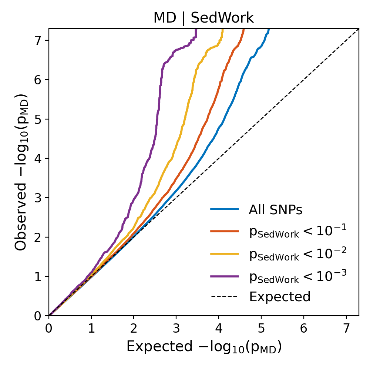

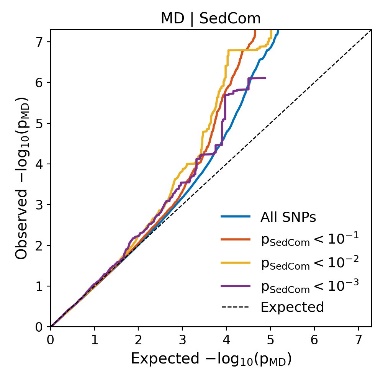

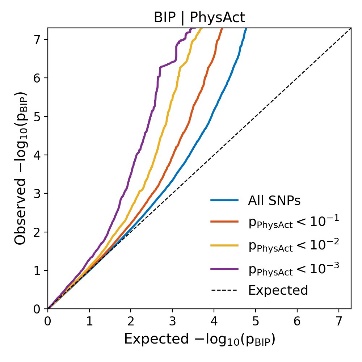

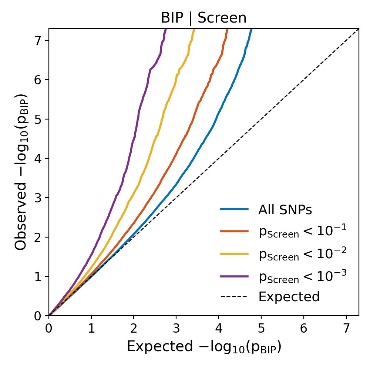

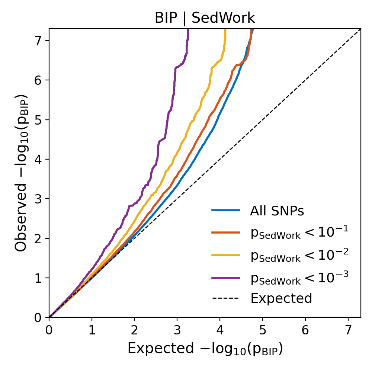

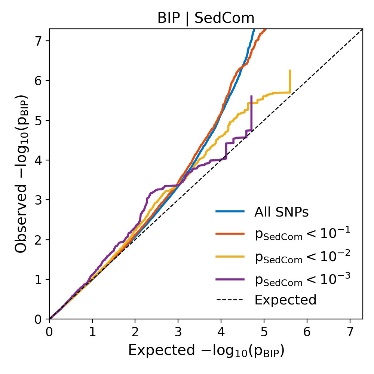

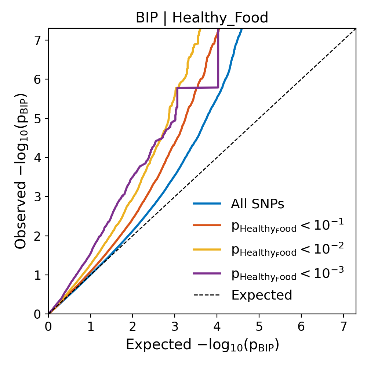

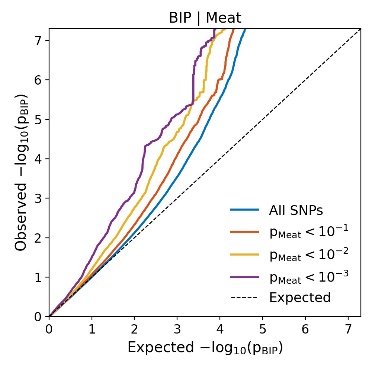


Conditional Q-Q plots of nominal versus empirical −log_10_ p values (corrected for inflation) in severe mental disorders below the standard GWAS threshold of p < 5 × 10^−8^ as a function of significance of association with lifestyle factors, at the level of p < 0.1, p < 0.01, p < 0.001, respectively. The blue lines indicate all SNPs. The dashed lines indicate the null hypothesis. MD, major depression; SCZ, schizophrenia; BIP, bipolar disorder; Healthy Food, healthy food intake; Meat, meat consumption; PhysAct, Moderate-to-vigorous intensity physical activity; Screen, leisure screen time; SedWork, sedentary behaviour at work; SedCom, sedentary commuting.

Conditional Q-Q plots of nominal versus empirical −log_10_ p values (corrected for inflation) in lifestyle factors below the standard GWAS threshold of p < 5 × 10^−8^ as a function of significance of association with severe mental disorders, at the level of p < 0.1, p < 0.01, p < 0.001, respectively. The blue lines indicate all SNPs. The dashed lines indicate the null hypothesis. MD, major depression; SCZ, schizophrenia; BIP, bipolar disorder; Healthy Food, healthy food intake; Meat, meat consumption; PhysAct, Moderate-to-vigorous intensity physical activity; Screen, leisure screen time; SedWork, sedentary behaviour at work; SedCom, sedentary commuting.


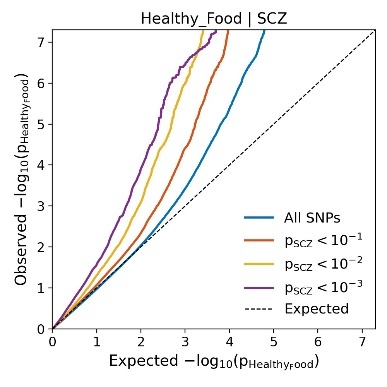

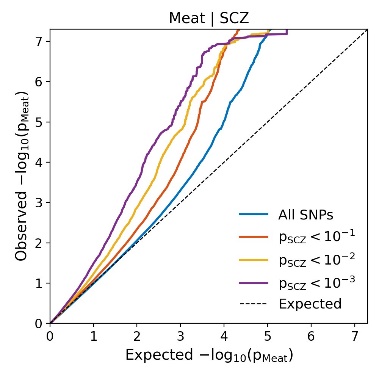

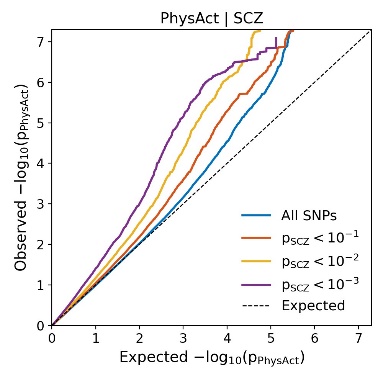

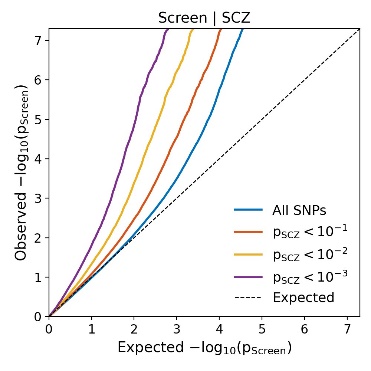

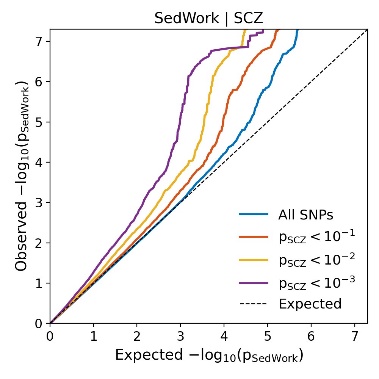

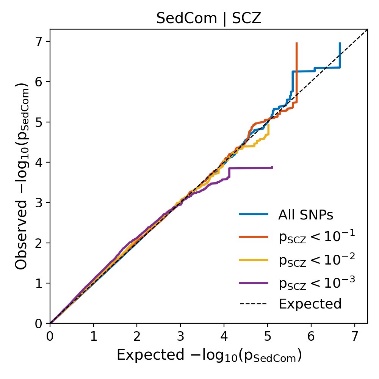

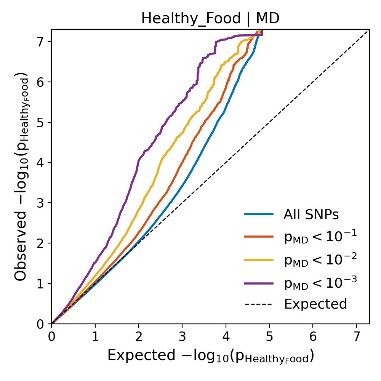

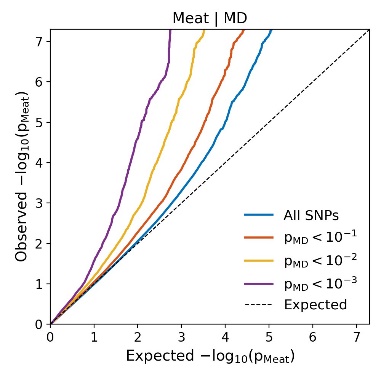

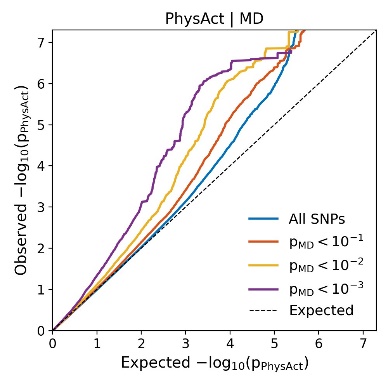

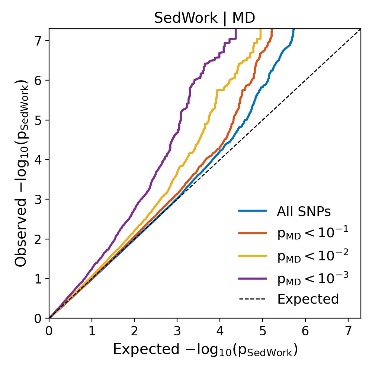

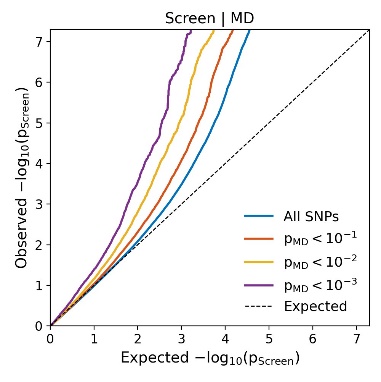

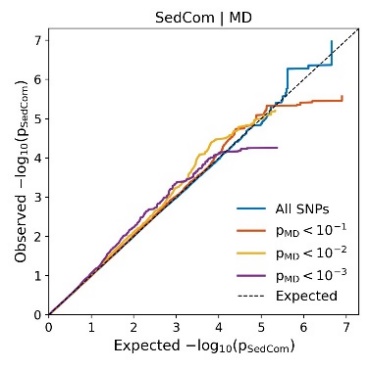

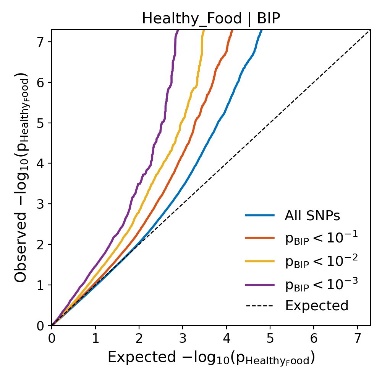

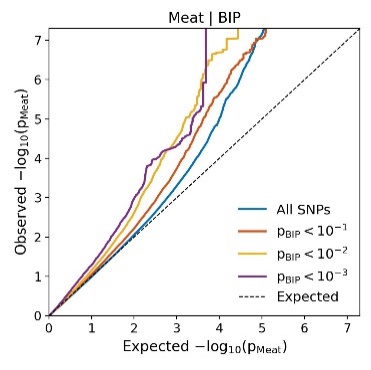

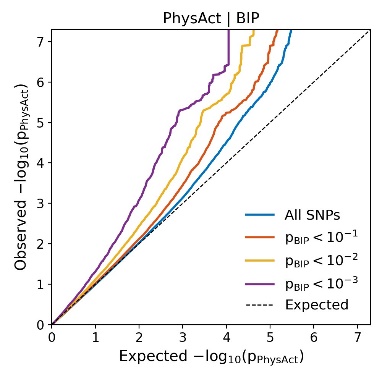

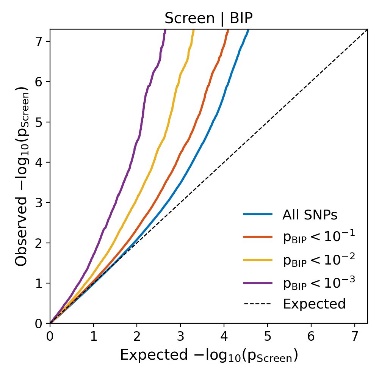

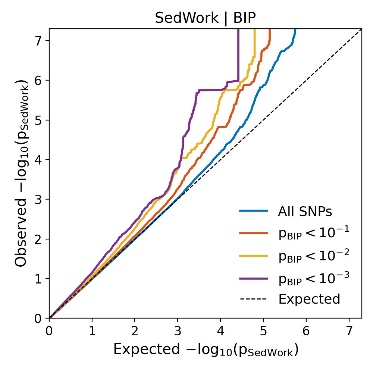

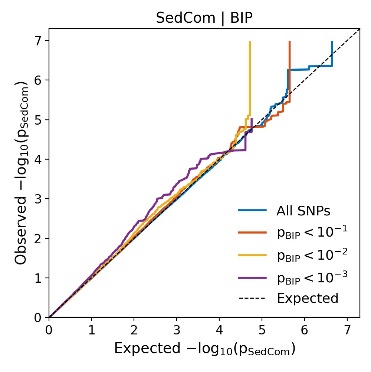


**Fig. S10. The conditional Q-Q plots for lifestyle conditional on severe mental disorders**


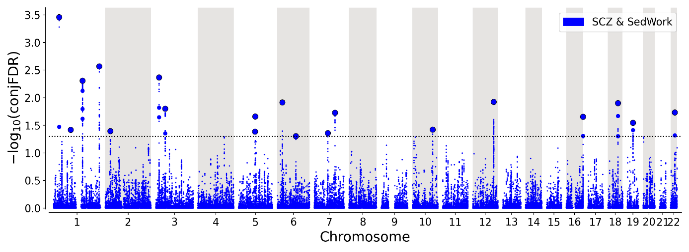

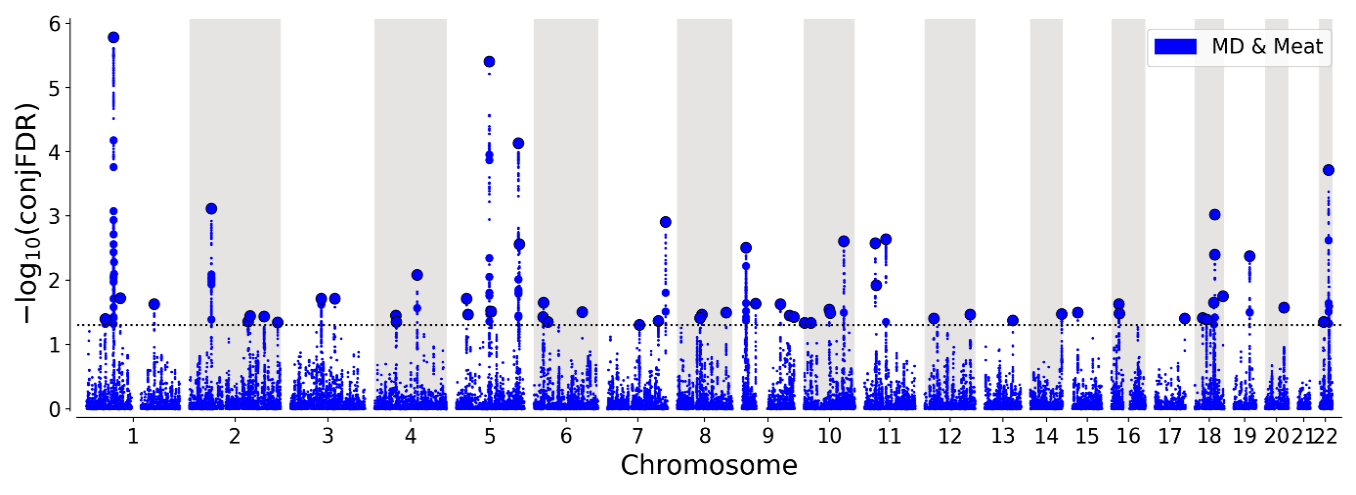


A)


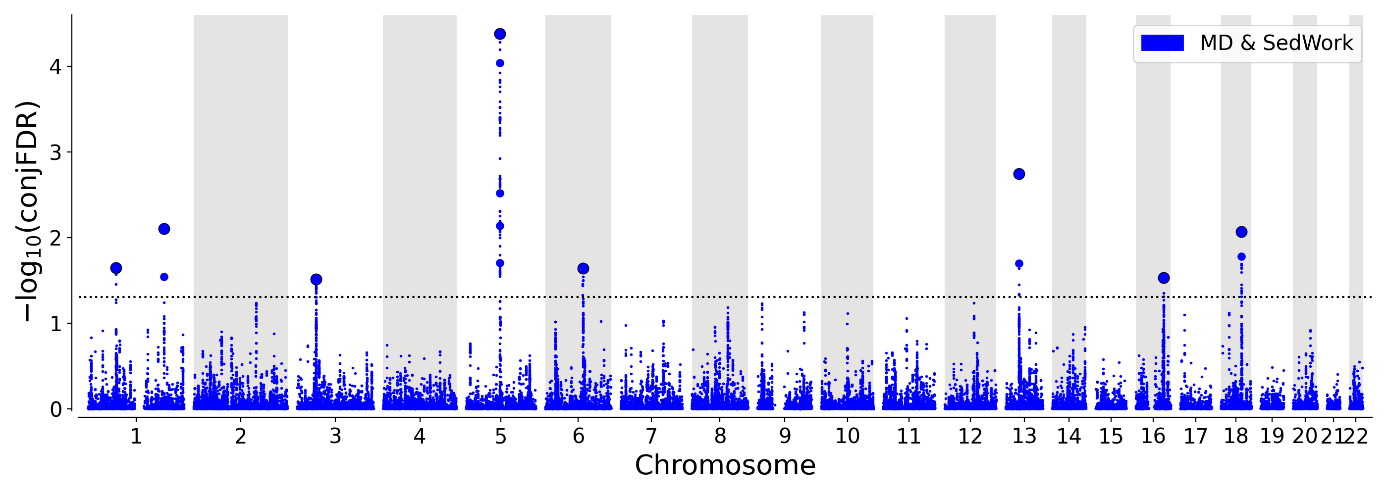


B)


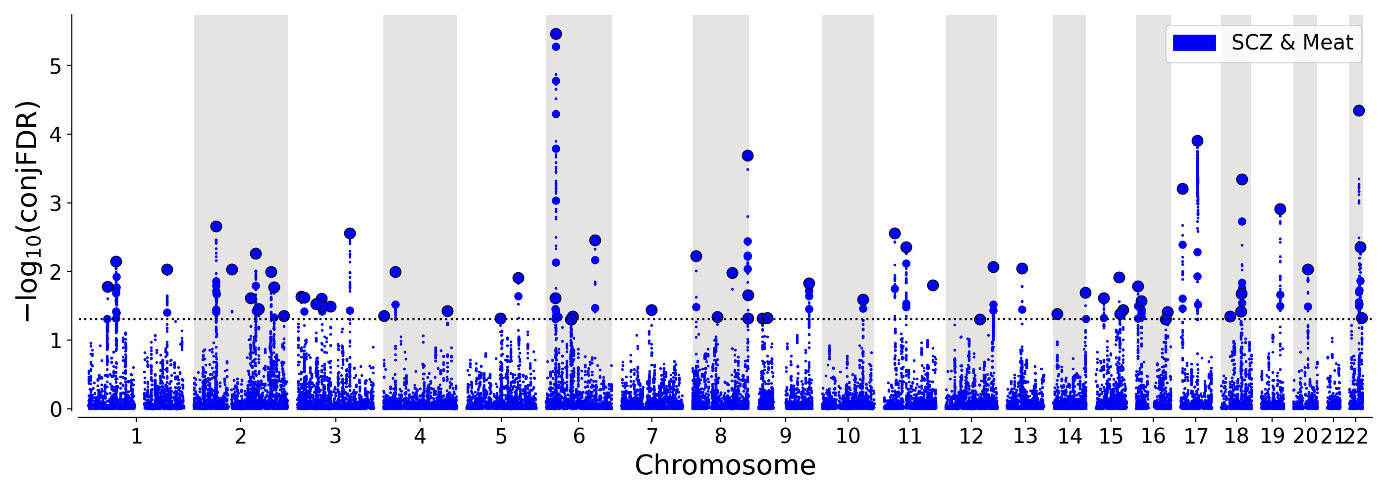


D)

C)

**Fig. S11. Common genetic variants jointly associated with severe mental disorders and lifestyle factors** **at conjFDR < 0.05**

The Manhattan plots show the common genetic variants jointly associated with major depression (MD) and A) meat consumption (Meat) and B) sedentary behaviour at work (SedWork) at conjunctional false discovery rate (conjFDR) <0.05. The Manhattan plots also show common genetic variants for schizophrenia (SCZ) and C) Meat and D) SedWork. Additionally, we present the common genetic variants for bipolar disorder (BIP) and E) Meat and F) SedWork. The plots show the –log_10_ transformed conjFDR values for each single-nucleotide polymorphism (SNP) on the y-axis and chromosomal positions along the x-axis. SNPs with conjFDR<0.05 (i.e., −log_10_ FDR >1.3) are shown with enlarged data points. A black circle around the enlarged data points indicates the most significant SNP in each LD block. The plots show the localization of the “conjunctional loci”.


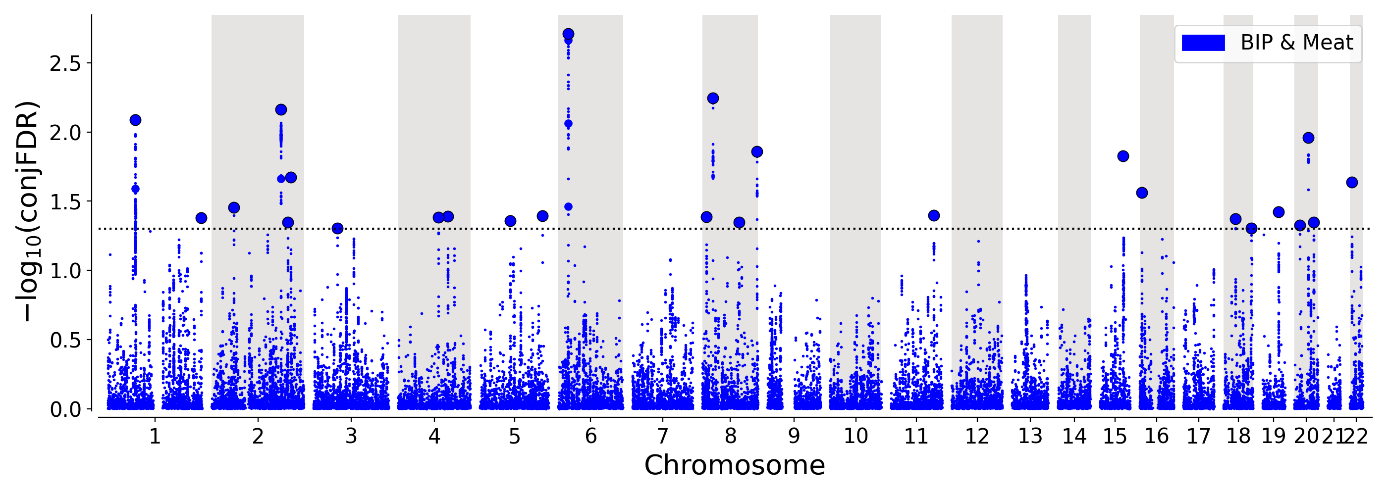


E)


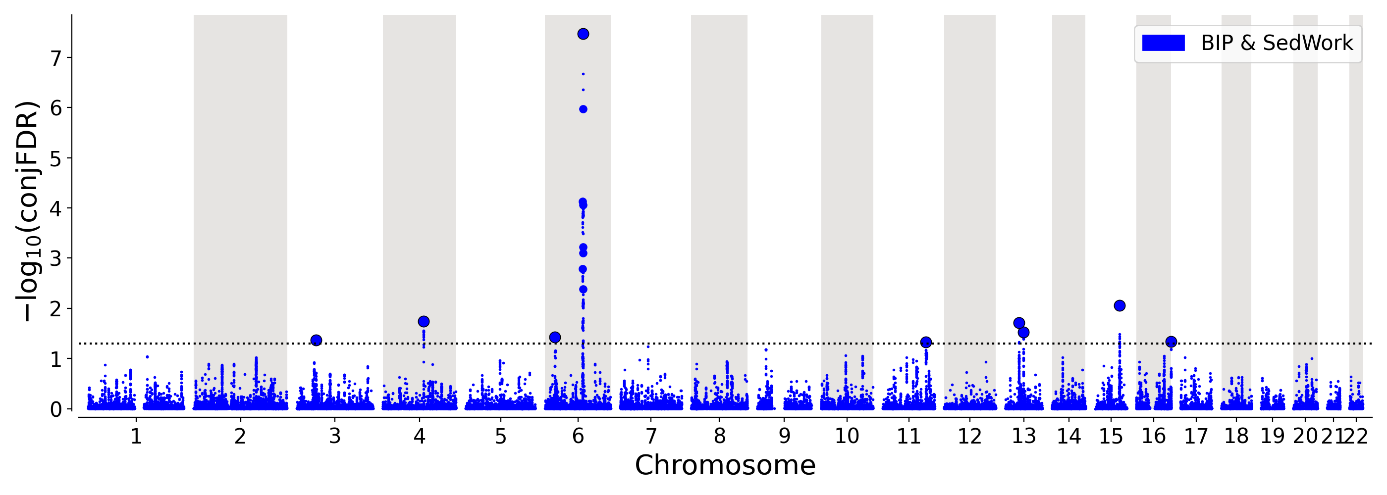


F)

**Fig. S12.** **Marginal and bivariate density distributions for models with *BMI***

**A. The distribution of residuals from SEM with *MD PRS* as a predictor**

**B. The distribution of residuals from SEM with *SCZ PRS* as a predictor**

**C. The distribution of residuals from SEM with *BIP PRS* as a predictor**

The figures show the distribution for the dependent variables’ (one mediator and one outcome) residuals in each SEM by PRS: Panel A, major depression (MD) PRS; Panel B, schizophrenia (SCZ) PRS; Panel C, bipolar disorder (BIP) PRS. The first two columns display the marginal density for the lifestyle mediator and the outcome variable (BMI), respectively. The third column presents the bivariate density (joint distribution of the two variables’ residuals). BMI, body mass index; Healthy Food, healthy food intake; Meat; meat consumption; Screen, leisure screen time.

**Fig. S13.** **Marginal and bivariate density distributions for models with HDL**

**A. The distribution of residuals from SEM with *MD PRS* as a predictor** **C. The distribution of residuals from SEM with *BIP PRS* as a predictor**

**B. The distribution of residuals from SEM with *SCZ PRS* as a predictor**

The figures show the distribution for the dependent variables’ (one mediator and one outcome) residuals in each SEM by PRS: Panel A, major depression (MD) PRS; Panel B, schizophrenia (SCZ) PRS; Panel C, bipolar disorder (BIP) PRS. The first two columns display the marginal density for the covariate (i.e., lifestyle factor as a mediator) and the outcome variable (HDL), respectively. The third column presents the bivariate density (joint distribution of the two variables’ residuals). HDL, high-density lipoprotein; Healthy Food, healthy food intake; Meat; meat consumption; Screen, leisure screen time.

**Fig. S14.** **Marginal and bivariate density distributions for models with TG**

**A. The distribution of residuals from SEM with *MD PRS* as a predictor**

**B. The distribution of residuals from SEM with *SCZ PRS* as a predictor**

**C. The distribution of residuals from SEM with *BIP PRS* as a predictor**

The figures show the distribution for the dependent variables’ (one mediator and one outcome) residuals in each SEM by PRS: Panel A, major depression (MD) PRS; Panel B, schizophrenia (SCZ) PRS; Panel C, bipolar disorder (BIP) PRS. The first two columns display the marginal density for the covariate (i.e., lifestyle factor as a mediator) and the outcome variable (TG), respectively. The third column presents the bivariate density (joint distribution of the two variables’ residuals). TG, triglycerides; Healthy Food, healthy food intake; Meat; meat consumption; Screen, leisure screen time.

The figure presents the standardized beta coefficients for each path (95% CI) from structural equation models, representing the association between polygenic risk score (PRS) for major depression (MD), schizophrenia (SCZ) and bipolar disorder (BIP), healthy food intake (Healthy Food) and body mass index (BMI). Separate structural equation models for each PRS are combined in the figure.

**Fig. S15. The direct and indirect associations between polygenic risk score for severe mental disorders and body mass index through healthy food intake**

The figure presents the standardized beta coefficients for each path (95% CI) from structural equation models, representing the association between polygenic risk score (PRS) for major depression (MD), schizophrenia (SCZ) and bipolar disorder (BIP), meat consumption (Meat) and body mass index (BMI). Separate structural equation models for each PRS are combined in the figure.

**Fig. S16. The direct and indirect associations between polygenic risk score for severe mental disorders and body mass index through meat consumption**

The figure presents the standardized beta coefficients for each path (95% CI) from structural equation models, representing the association between polygenic risk score (PRS) for major depression (MD), schizophrenia (SCZ) and bipolar disorder (BIP), moderate-to-vigorous physical activity (PhysAct) and body mass index (BMI). Separate structural equation models for each PRS are combined in the figure.

**Fig. S17. The direct and indirect associations between polygenic risk score for severe mental disorders and body mass index through moderate-to-vigorous physical activity**

The figure presents the standardized beta coefficients for each path (95% CI) from structural equation models, representing the association between polygenic risk score (PRS) for major depression (MD), schizophrenia (SCZ) and bipolar disorder (BIP), leisure screen time (Screen) and body mass index (BMI). Separate structural equation models for each PRS are combined in the figure.

**Fig. S18. The direct and indirect associations between polygenic risk score for severe mental disorders and body mass index through leisure screen time**

Univariate Q-Q plots for distribution of expected p values under a null model (no SNPs associated with the phenotype) (x axis) versus observed p values (y axis). Univariate Q-Q plots demonstrate that MiXeR-based predictions provide accurate estimates of the data Q-Q plots except for SedCom. Blue lines indicate p values of SNPs observed in GWAS summary statistics with grey shading indicating 95% confidence interval. Orange lines indicate model predictions. The dashed line is the expected Q-Q plot under null (no SNPs associated with the phenotype). The vertical axes are limited to the genome-wide significance threshold of p < 5×10^−8^, to highlight behaviour of polygenic component. Points on the Q-Q plot are weighted according to LD structure, using n = 64 iterations of random pruning at LD threshold r^2^ = 0.1. AccPhys, accelerometer-assessed physical activity; AccSed, accelerometer-assessed sedentary behaviour.

AccPhysAct

AccSed

**Fig. S19. Univariate Q-Q plots from MiXeR of accelerometer-assessed physical activity and sedentary behaviour**

Venn Diagrams, conditional Q-Q plots, and negative log-likelihood plot, respectively. Venn diagrams of shared and unique trait-influencing variants, showing polygenic overlap (gray) between major depression (blue) and accelerometer phenotypes (orange). The numbers in the Venn diagram indicate the estimated quantity of trait-influencing variants (in thousands), followed by standard error. Appearance of the Q-Q plot and negative log-likelihood plot is described below Fig. S2. MD, major depression; AccPhysAct, accelerometer-assessed physical activity; AccSed, accelerometer-assessed sedentary behaviour.

**Fig. S20. Bivariate MiXeR predictions for major depression and accelerometer-assessed physical activity and sedentary behaviour**

Venn Diagrams, conditional Q-Q plots, and negative log-likelihood plot, respectively. Venn diagrams of shared and unique trait-influencing variants, showing polygenic overlap (gray) between schizophrenia (blue) and accelerometer phenotypes (orange). The numbers in the Venn diagram indicate the estimated quantity of trait-influencing variants (in thousands), followed by standard error. Appearance of the Q-Q plot and negative log-likelihood plot is described below Fig. S2. SCZ, schizophrenia; AccPhysAct, accelerometer-assessed physical activity; AccSed, accelerometer-assessed sedentary behaviour.

**Fig. S21. Bivariate MiXeR predictions for schizophrenia and accelerometer-assessed physical activity and sedentary behaviour**

Venn Diagrams, conditional Q-Q plots, and negative log-likelihood plot, respectively. Venn diagrams of shared and unique trait-influencing variants, showing polygenic overlap (gray) between bipolar disorder (blue) and accelerometer phenotypes (orange). The numbers in the Venn diagram indicate the estimated quantity of trait-influencing variants (in thousands), followed by standard error. Appearance of the Q-Q plot and negative log-likelihood plot is described below Fig. S2. BIP, bipolar disorder; AccPhysAct, accelerometer-assessed physical activity; AccSed, accelerometer-assessed sedentary behaviour.

**Fig. S22. Bivariate MiXeR predictions for bipolar disorder and accelerometer-assessed physical activity and sedentary behaviour**

**References**

1. Hindley G, Frei O, Shadrin AA, et al. Charting the Landscape of Genetic Overlap Between Mental Disorders and Related Traits Beyond Genetic Correlation. Am J Psychiatry. 2022;179(11):833–43.

2. Frei O, Holland D, Smeland OB, et al. Bivariate causal mixture model quantifies polygenic overlap between complex traits beyond genetic correlation. Nat Commun. 2019;10(1):2417–.

3. Holland D, Frei O, Desikan R, et al. Beyond SNP heritability: Polygenicity and discoverability of phenotypes estimated with a univariate Gaussian mixture model. PLoS Genet. 2020;16(5):e1008612.

4. Bulik-Sullivan BK, Loh P-R, Finucane HK, et al. LD Score regression distinguishes confounding from polygenicity in genome-wide association studies. Nat Genet. 2015;47(3):291–5.

5. Shadrin AA, Hindley G, Hagen E, et al. Distinct patterns of genetic overlap among multimorbidities revealed with trivariate MiXeR. Genome Med. 2025;17(1):106.

6. Shadrin AA, Frei O, Smeland OB, et al. Phenotype-specific differences in polygenicity and effect size distribution across functional annotation categories revealed by AI-MiXeR. Bioinformatics. 2020;36(18):4749–56.

7. Schweder T, Spjotvoll E. Plots of P-Values to Evaluate Many Tests Simultaneously. Biometrika. 1982;69(3):493–502.

8. Efron B. Size, power and false discovery rates. The Annals of Statistics. 2007;35(4):1351–77.

9. Benjamini Y, Hochberg Y. Controlling the False Discovery Rate: A Practical and Powerful Approach to Multiple Testing. Journal of the Royal Statistical Society Series B (Methodological). 57: Blackwell Publishing; 1995. p. 289–300.

10. Purcell S, Neale B, Todd-Brown K, et al. PLINK: a tool set for whole-genome association and population-based linkage analyses. Am J Hum Genet. 2007;81(3):559–75.

11. Andreassen OA, Djurovic S, Thompson WK, et al. Improved detection of common variants associated with schizophrenia by leveraging pleiotropy with cardiovascular-disease risk factors. Am J Hum Genet. 2013;92(2):197–209.

12. Andreassen OA, Thompson WK, Schork AJ, et al. Improved detection of common variants associated with schizophrenia and bipolar disorder using pleiotropy-informed conditional false discovery rate. PLoS Genet. 2013;9(4):e1003455.

13. Andreassen OA, Harbo HF, Wang Y, et al. Genetic pleiotropy between multiple sclerosis and schizophrenia but not bipolar disorder: differential involvement of immune-related gene loci. Mol Psychiatry. 2015;20(2):207–14.

14. Andreassen OA, Thompson WK, Dale AM. Boosting the power of schizophrenia genetics by leveraging new statistical tools. Schizophr Bull. 2014;40(1):13–7.

15. Andreassen OA, Desikan RS, Wang Y, et al. Abundant genetic overlap between blood lipids and immune-mediated diseases indicates shared molecular genetic mechanisms. PLoS One. 2015;10(4):e0123057.

16. Nichols T, Brett M, Andersson J, Wager T, Poline JB. Valid conjunction inference with the minimum statistic. Neuroimage. 2005;25(3):653–60.

17. Schwartzman A, Lin X. The effect of correlation in false discovery rate estimation. Biometrika. 2011;98(1):199–214.

18. Smeland, Frei O, Shadrin A, et al. Discovery of shared genomic loci using the conditional false discovery rate approach. Hum Genet. 2020;139(1):85–94.

19. Rodevand L, Bahrami S, Frei O, et al. Extensive bidirectional genetic overlap between bipolar disorder and cardiovascular disease phenotypes. Transl Psychiatry. 2021;11(1):407.

20. Bahrami S, Steen NE, Shadrin A, et al. Shared Genetic Loci Between Body Mass Index and Major Psychiatric Disorders: A Genome-wide Association Study. JAMA Psychiatry. 2020;77(5):503–12.

21. Watanabe K, Taskesen E, van Bochoven A, Posthuma D. Functional mapping and annotation of genetic associations with FUMA. Nat Commun. 2017;8(1):1826.

22. The 1000 Genomes Project Consortium. A global reference for human genetic variation. Nature. 2015;526(7571):68–74.

23. Trubetskoy V, Pardiñas AF, Qi T, et al. Mapping genomic loci implicates genes and synaptic biology in schizophrenia. Nature. 2022.

24. Mullins N, Forstner AJ, O’Connell KS, et al. Genome-wide association study of more than 40,000 bipolar disorder cases provides new insights into the underlying biology. Nat Genet. 2021;53(6):817–29.

25. Wray NR, Ripke S, Mattheisen M, et al. Genome-wide association analyses identify 44 risk variants and refine the genetic architecture of major depression. Nat Genet. 2018;50(5):668–81.

26. Pirastu N, McDonnell C, Grzeszkowiak EJ, et al. Using genetic variation to disentangle the complex relationship between food intake and health outcomes. PLoS Genet. 2022;18(6):e1010162.

27. Wang Z, Emmerich A, Pillon NJ, et al. Genome-wide association analyses of physical activity and sedentary behavior provide insights into underlying mechanisms and roles in disease prevention. Nat Genet. 2022;54(9):1332–44.

28. Bulik-Sullivan B, Finucane HK, Anttila V, et al. An atlas of genetic correlations across human diseases and traits. Nat Genet. 2015;47(11):1236–41.

29. Ghoussaini M, Mountjoy E, Carmona M, et al. Open Targets Genetics: systematic identification of trait-associated genes using large-scale genetics and functional genomics. Nucleic Acids Res. 2020;49(D1):D1311–D20.

30. Morris JA, Daniloski Z, Domingo J, et al. Discovery of target genes and pathways of blood trait loci using pooled CRISPR screens and single cell RNA sequencing. bioRxiv. 2021:2021.04.07.438882.

31. Ashburner M, Ball CA, Blake JA, et al. Gene ontology: tool for the unification of biology. The Gene Ontology Consortium. Nat Genet. 2000;25(1):25–9.

32. Rødevand L, Rahman Z, Hindley GF, et al. Characterizing the shared genetic underpinnings of schizophrenia and cardiovascular disease risk factors. Am J Psychiatry. 2023;180(11):815–26.

33. Reponen EJ, Ueland T, Rokicki J, et al. Polygenic risk for schizophrenia and bipolar disorder in relation to cardiovascular biomarkers. Eur Arch Psychiatry Clin Neurosci 2024;274(5):1223–30.

34. Rødevand L, Bahrami S, Frei O, et al. Extensive bidirectional genetic overlap between bipolar disorder and cardiovascular disease phenotypes. Transl Psychiatry. 2021;11(1):407.

35. Bergstedt J, Pasman JA, Ma Z, et al. Distinct biological signature and modifiable risk factors underlie the comorbidity between major depressive disorder and cardiovascular disease. Nat Cardiovasc Res. 2024;3(6):754–69.

36. Choi SW, O'Reilly PF. PRSice-2: Polygenic Risk Score software for biobank-scale data. GigaScience. 2019;8(7).

37. Rosseel Y. lavaan: An R Package for Structural Equation Modeling. Journal of Statistical Software. 2012;48(2):1 – 36.

38. Moshagen M, Bader M. semPower: General power analysis for structural equation models. Behav Res Methods. 2024;56(4):2901–22.

39. Knief U, Forstmeier W. Violating the normality assumption may be the lesser of two evils. Behav Res Methods. 2021;53(6):2576–90.

40. Beasley TM, Erickson S, Allison DB. Rank-based inverse normal transformations are increasingly used, but are they merited? Behav Genet. 2009;39(5):580–95.

41. Sanderson E, Glymour MM, Holmes MV, et al. Mendelian randomization. Nature Reviews Methods Primers. 2022;2(1):6.

42. Burgess S, Butterworth A, Thompson SG. Mendelian randomization analysis with multiple genetic variants using summarized data. Genet Epidemiol. 2013;37(7):658–65.

43. Bowden J, Davey Smith G, Burgess S. Mendelian randomization with invalid instruments: effect estimation and bias detection through Egger regression. Int J Epidemiol. 2015;44(2):512–25.

44. Bowden J, Davey Smith G, Haycock PC, Burgess S. Consistent Estimation in Mendelian Randomization with Some Invalid Instruments Using a Weighted Median Estimator. Genet Epidemiol. 2016;40(4):304–14.

45. Hemani G, Zheng J, Elsworth B, et al. The MR-Base platform supports systematic causal inference across the human phenome. Elife. 2018;7.

46. Verbanck M, Chen CY, Neale B, Do R. Detection of widespread horizontal pleiotropy in causal relationships inferred from Mendelian randomization between complex traits and diseases. Nat Genet. 2018;50(5):693–8.

47. How the All of Us Genomic data are organized (Archived C2022Q4R13 CDRv7) (cited 2026, March 13) 2025. [Available from: <https://support.researchallofus.org/hc/en-us/articles/4614687617556-How-the-All-of-Us-Genomic-data-are-organized-Archived-C2022Q4R13-CDRv7#h_01GY7QVJ6QZ41B4SMW00NYPX27>].

48. Bick AG, Metcalf GA, Mayo KR, et al. Genomic data in the All of Us Research Program. Nature. 2024;627(8003):340–6.

49. Chang CC, Chow CC, Tellier LC, Vattikuti S, Purcell SM, Lee JJ. Second-generation PLINK: rising to the challenge of larger and richer datasets. Gigascience. 2015;4:7.

50. Gadin JR, Zetterberg R, Meijsen J, Schork AJ. Cleansumstats: Converting GWAS sumstats to a common format to facilitate downstream applications. 2022.

51. Willer CJ, Li Y, Abecasis GR. METAL: fast and efficient meta-analysis of genomewide association scans. Bioinformatics. 2010;26(17):2190–1.

52. Doherty A, Smith-Byrne K, Ferreira T, et al. GWAS identifies 14 loci for device-measured physical activity and sleep duration. Nat Commun. 2018;9(1):5257.
